# A highly contiguous genome assembly of the bat hawkmoth *Hyles vespertilio* (Lepidoptera: Sphingidae)

**DOI:** 10.1093/gigascience/giaa001

**Published:** 2020-01-23

**Authors:** Martin Pippel, David Jebb, Franziska Patzold, Sylke Winkler, Heiko Vogel, Gene Myers, Michael Hiller, Anna K Hundsdoerfer

**Affiliations:** Max Planck Institute of Molecular Cell Biology and Genetics, Pfotenhauerstr. 108, 01307 Dresden, Germany; Center for Systems Biology Dresden, Pfotenhauerstr. 108, 01307 Dresden, Germany; Max Planck Institute of Molecular Cell Biology and Genetics, Pfotenhauerstr. 108, 01307 Dresden, Germany; Center for Systems Biology Dresden, Pfotenhauerstr. 108, 01307 Dresden, Germany; Max Planck Institute for the Physics of Complex Systems, Nöthnitzer Str. 38, 01187 Dresden, Germany; Senckenberg Natural History Collections Dresden, Königsbrücker Landstr. 159, 01109 Dresden, Germany; Max Planck Institute of Molecular Cell Biology and Genetics, Pfotenhauerstr. 108, 01307 Dresden, Germany; Department of Entomology, Max Planck Institute for Chemical Ecology, Hans-Knoell-Str. 8, 07745 Jena, Germany; Max Planck Institute of Molecular Cell Biology and Genetics, Pfotenhauerstr. 108, 01307 Dresden, Germany; Center for Systems Biology Dresden, Pfotenhauerstr. 108, 01307 Dresden, Germany; Max Planck Institute of Molecular Cell Biology and Genetics, Pfotenhauerstr. 108, 01307 Dresden, Germany; Center for Systems Biology Dresden, Pfotenhauerstr. 108, 01307 Dresden, Germany; Max Planck Institute for the Physics of Complex Systems, Nöthnitzer Str. 38, 01187 Dresden, Germany; Senckenberg Natural History Collections Dresden, Königsbrücker Landstr. 159, 01109 Dresden, Germany

**Keywords:** genome assembly, PacBio long reads, hawkmoth–silk moth comparison, gene annotation

## Abstract

**Background:**

Adapted to different ecological niches, moth species belonging to the *Hyles* genus exhibit a spectacular diversity of larval color patterns. These species diverged ~7.5 million years ago, making this rather young genus an interesting system to study a wide range of questions including the process of speciation, ecological adaptation, and adaptive radiation.

**Results:**

Here we present a high-quality genome assembly of the bat hawkmoth *Hyles vespertilio*, the first reference genome of a member of the *Hyles* genus. We generated 51× Pacific Biosciences long reads with an average read length of 8.9 kb. Pacific Biosciences reads longer than 4 kb were assembled into contigs, resulting in a 651.4-Mb assembly consisting of 530 contigs with an N50 value of 7.5 Mb. The circular mitochondrial contig has a length of 15,303 bp. The *H. vespertilio* genome is very repeat-rich and exhibits a higher repeat content (50.3%) than other Bombycoidea species such as *Bombyx mori* (45.7%) and *Manduca sexta* (27.5%). We developed a comprehensive gene annotation workflow to obtain consensus gene models from different evidence including gene projections, protein homology, transcriptome data, and *ab initio* predictions. The resulting gene annotation is highly complete with 94.5% of BUSCO genes being completely present, which is higher than the BUSCO completeness of the *B. mori* (92.2%) and *M. sexta* (90%) annotations.

**Conclusions:**

Our gene annotation strategy has general applicability to other genomes, and the *H. vespertilio* genome provides a valuable molecular resource to study a range of questions in this genus, including phylogeny, incomplete lineage sorting, speciation, and hybridization. A genome browser displaying the genome, alignments, and annotations is available at https://genome-public.pks.mpg.de/cgi-bin/hgTracks?db=HLhylVes1.

## Introduction

Bombycoidea are a speciose superfamily of moths, comprising 10 families, >500 genera [1], and 6,092 species that are mostly diversified in the intertropical region of the globe [2]. This superfamily includes the 2 well-known macrolepidopteran families, Saturniidae and Sphingidae. The larvae of ≥8 Saturniid species are eaten as an important source of proteins in rural Africa [3]. With wingspans of 4–10 cm, sphingids are large pollinators with excellent flying abilities. They are important prey for bats, and some species can produce ultrasound to divert attacks by echolocating bats [4]. Furthermore, Bombycoidea not only comprises some of the largest moth species, exemplified by the giant silk moth *Attacus atlas* with a wingspan measuring 25–30 cm, but also includes several model organisms, such as the domestic silkmoth *Bombyx mori*, a bombycid of great economic importance for silk production, and the tobacco hornworm *Manduca sexta*, which is a common pest sphingid species causing considerable damage to tobacco, tomato, pepper, eggplant, and plantations of other crops [5]. Because these species have been extensively studied, they play a leading role in the fields of Lepidoptera genetics and physiology. To date, genomes of only 4 Bombycoidea species have been published (*B. mori* [6] together with its 2 closely related congeners *Bombyx huttoni* and *Bombyx mandarina* of the Bombycidae, and *M. sexta* [7] of the Sphingidae), which represents a tiny fraction of the diversity of Bombycoidea.

Sphingidae include the hawkmoth genus *Hyles*. This genus originated in South America and comprises 32 recognized species [8–10], with representatives native to all continents and major islands (except Antarctica). As a rather young genus, estimated to have diverged ~7.5 million years ago [4], species from different continents are still able to hybridize. This makes *Hyles* an interesting genus to study a wide range of questions including the process of speciation, ecological adaptation, adaptive radiation, genetics of reproduction, and evolution in action. However, such studies are hampered by the lack of suitable molecular resources. In particular, a well-assembled reference genome for any *Hyles* species is currently missing.

**Figure 1:**
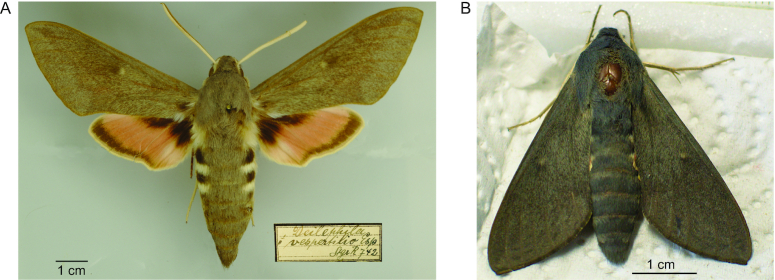
*Hyles vespertilio*. (A) The lectotype specimen, originally described as *Sphinx vespertilio* by Esper in 1779, was collected in the “area of Verona” (Italy) and deposited in the Landesmuseum für Kunst und Natur (Wiesbaden, Germany). (B) The specimen collected in Vallonina, Italy, in 2018. Nearly all tissue was used to sequence the genome. The wings are deposited in the Museum of Zoology (Senckenberg Natural History Collections Dresden, Germany). Scale bars: 1 cm.

Here we present a high-quality nuclear and mitochondrial genome assembly of the bat hawkmoth *Hyles vespertilio* (NCBI:txid283848) (Fig. 1), the first reference genome assembly of a member of the *Hyles* genus. Compared with other members of the *Hyles* genus that have broad species distributions, *H. vespertilio* is restricted to mountainous river valleys in the Western Palearctic. This rather restricted distribution is likely associated with a lower degree of hybridization, which makes *H. vespertilio* a good target for assembly of a reference genome. In the following, we report the assembly of the *H. vespertilio* genome from using Pacific Biosciences (PacBio) long sequencing reads, the annotation of this genome, and a comparative analysis to *B. mori* and *M. sexta*. All data can be visualized and downloaded from a genome browser instance at [11].

## Results

### Assembly of *Hyles vespertilio* from long sequencing reads

**Figure 2:**
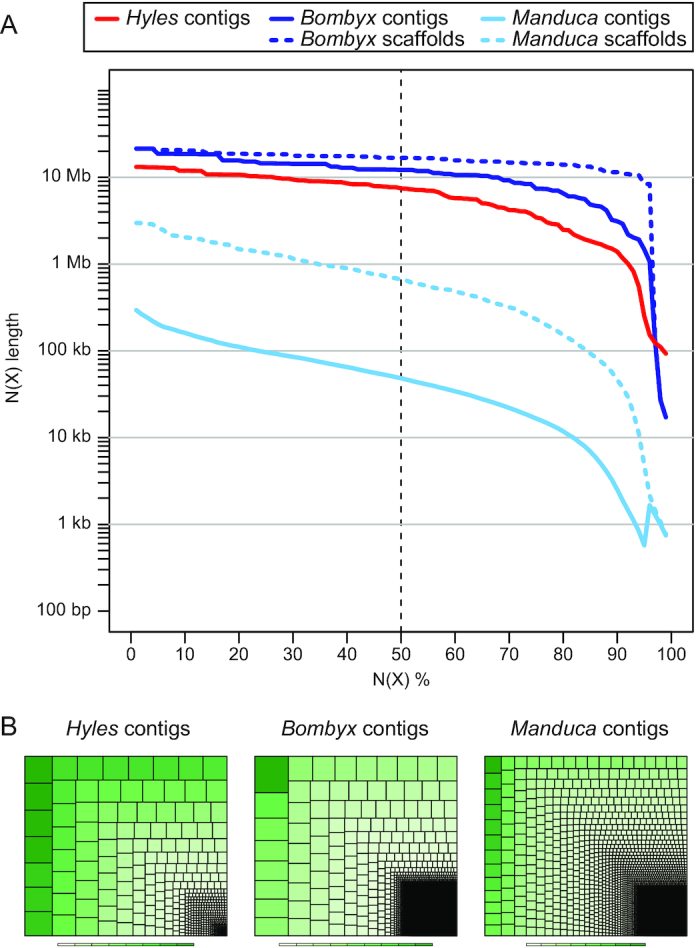
Assembly contiguity. (A) The N(x)% graph shows the contig or scaffold sizes on the y-axis, where x% of the genome assembly consists of contigs or scaffolds of at least that size. Contigs are shown as solid curves, scaffolds (only for *B. mori* and *M. sexta*) as dashed curves. The N50 value is marked as a vertical dashed line. (B) Treemap comparison between the DAmar *H. vespertilio* assembly and the assemblies of *B. mori* [6] and *M. sexta* [7]. Squares encode the relative contributions of individual scaffolds or contigs to assembly size.

We generated 51× PacBio long reads with an average read length of 8.9 kb and an N50 read length of 16.5 kb. PacBio reads longer than 4 kb were assembled into contigs with a customized assembler called DAmar. DAmar is a hybrid of our MARVEL approach, which was used to assemble the Axolotl [12] and *Schmidtea* [13] genomes, and the Dazzler method (Supplementary Text 1). This resulted in a 651.4-Mb assembly consisting of 530 contigs. The contig N50 value is 7.5 Mb (Fig. 2A).

Compared with the assembly of *B. mori* (460.3 Mb) and *M. sexta* (419.4 Mb), the *H. vespertilio* assembly is substantially larger by 191 and 232 Mb, respectively. As shown in Fig. 2, our contigs are >100 times longer than the contigs of the *M. sexta* assembly (N50 value, 52 kb) but shorter than the contigs of the *B. mori* assembly (N50 value, 12.2 Mb), which was based on a combination of different sequencing technologies including 80× PacBio long reads, 60× Illumina reads, and complete sequences of bacterial artificial chromosome and Fosmid clones. To assess and compare genome completeness, we used BUSCO [14] and the set of 2,442 conserved, single-copy Endopterygota genes. As shown in Supplementary Table 1, the *H. vespertilio* assembly (95.58% complete genes) shows a level of completeness similar to that of *B. mori* (96.48%) and *M. sexta* (95.21%).

We also assembled the mitochondrial genome of *H. vespertilio* using PacBio long reads and the DAmar assembler. The resulting circular mitochondrial contig has a length of 15,303 bases.

### Repeat content

**Figure 3:**
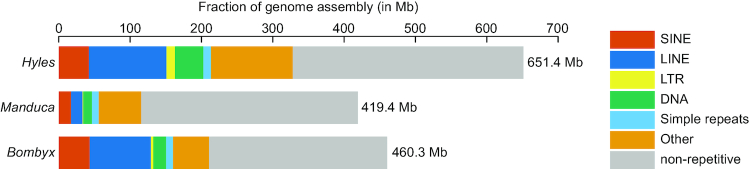
Comparison of genomic repeat content. Stacked bar charts represent the portion of the genome assembly covered by major classes of repetitive elements. Grey indicates non-repetitive genomic regions. Simple repeats comprise tandem repeats, low-complexity regions, and satellite repeats. The total assembly size is provided to the right of the bars.

To assess to which extent the *H. vespertilio* assembly consists of repetitive sequences, we modelled and masked repeats using RepeatModeler and RepeatMasker. To compare repeat content with *B. mori* and *M. sexta*, we applied the same procedure to these genomes as well. We found that the *H. vespertilio* genome has a high repeat content of 50.3% (Fig. 3, Supplementary Table 2). *B. mori* also has a repeat-rich genome (45.7%), while the *M. sexta* assembly is less repeat-rich (27.5%), which may be an underestimation because similar repeat copies may not be properly assembled for this species. This analysis also suggests that expansions of transposons and other repeats contributed to the larger genome size of *H. vespertilio* because the *H. vespertilio* assembly comprises 117 Mb more in repetitive elements compared to *B. mori* (328 vs 210.5 Mb). Long interspersed nuclear elements (LINEs) comprise the largest repeat class in the *H. vespertilio* assembly, followed by short interspersed nuclear elements (SINEs) and DNA transposons (Fig. 3). This is similar to *B. mori* and *M. sexta*, except that the latter exhibits fewer LINEs.

### Gene annotation

**Figure 4:**
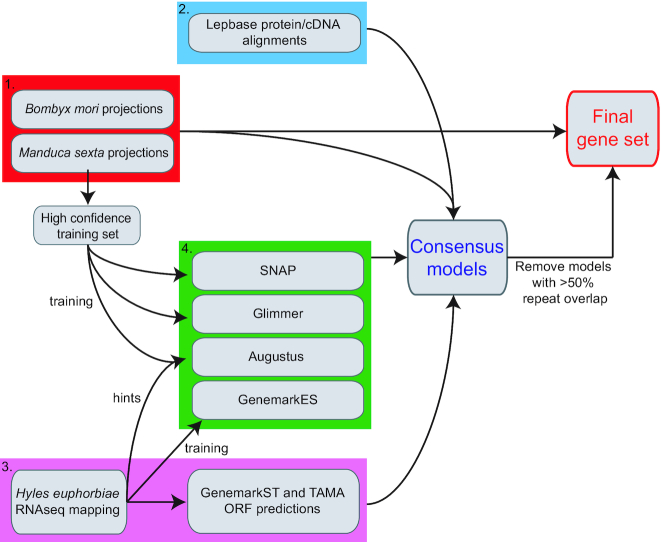
Gene annotation strategy. First, genome alignments were used to project coding gene annotation from *B. mori* and *M. sexta* to *H. vespertilio* with CESAR 2.0 (red box). Second, protein and coding sequences from 22 Lepidopteran species were downloaded from LepBase and aligned to the *H. vespertilio* genome (blue box). Third, RNA-seq reads from a related *Hyles* species were aligned to the genome and used to assemble transcripts, followed by predicting open reading frames (ORFs) with 2 methods (purple box). Fourth, high-quality gene projections and/or RNA-seq evidence were used to train 4 *ab initio* gene prediction tools (green box). All evidence was combined into a consensus set using EVM and filtered for models that overlap genomic repeats. Finally, these filtered gene models were combined with full-length gene projections that did not overlap the consensus gene model to produce the final gene set. cDNA: complementary DNA.

To annotate genes in the *H. vespertilio* assembly, we used Evidence Modeller (EVM) [15] to produce consensus gene models from multiple sources of predictions. A flowchart visualizing the gene annotation strategy is shown in Fig. 4. First we generated pairwise genome alignments between the *H. vespertilio* assembly and *B. mori* and *M. sexta*, and used these alignments to project annotated genes from *B. mori* and *M. sexta* to the *H. vespertilio* genome using CESAR [16, 17]. This resulted in 11,721 and 19,760 gene predictions that were projected from *B. mori* and *M. sexta*, respectively. Second, coding sequences (CDS) from 22 available lepidopteran species were downloaded from LepBase [18]. CDS and translated protein sequences were aligned to the *H. vespertilio* assembly using GenomeThreader [19]. The number of significant alignments is listed in Supplementary Table 3. Third, RNA sequencing (RNA-seq) data from *H. euphorbiae*, a closely related species, were mapped to the *H. vespertilio* assembly and assembled into transcripts using StringTie [20]. TAMA and GenemarkS-T [21] were used to predict open reading frames (ORFs) in assembled transcripts, predicting 45,488 and 19,776 coding transcripts, respectively. Fourth, 4 *ab initio* gene finders, SNAP, GlimmerHMM, Genemark-ES, and Augustus [22–25], predicted 67,351, 108,570, 92,757, and 61,225 gene models, respectively. All evidence was passed to EVM to produce a consensus gene set. Likely missing genes were recovered by keeping the *M. sexta* and *B. mori* CESAR projections, generating a set of 50,612 loci. Finally, models for which >50% of the CDS was contained within repeat sequence were removed. This produced a final set of 23,768 genes.

To assess the completeness of our *H. vespertilio* gene annotation, we used BUSCO [14] and the set of 2,442 conserved, single-copy Endopterygota genes. We found that our annotation is highly complete, with 94.4% (2,306 of 2,442) of these BUSCO genes being completely present (91.97% single copy, 2.46% duplicated genes). Importantly, this completeness is higher than the gene annotations of both *B. mori* (92.2% complete BUSCO genes) and *M. sexta* (90% complete BUSCO genes).

## Discussion

Here we present a high-quality genome assembly for the bat hawkmoth *Hyles vespertilio*. PacBio long reads have been instrumental to assemble long contigs, in particular because the *H. vespertilio* genome is longer and more repeat-rich than other Bombycoidea species. With a contig N50 value of 7.5 Mb, our assembly is the second-most contiguous Bombycoidea genome to date.

To annotate coding genes, we developed a strategy that integrates multiple different pieces of gene evidence. First, we used genome alignments to project genes annotated in related species. Gene projection generally produces very accurate annotations but is by definition limited by the completeness of the gene sets of related species. Evolutionary distance is another factor influencing the completeness of results obtained from gene projections, with closely related species generally allowing for more complete projections [17], consistent with our result that substantially more genes were projected using *M. sexta* as a reference. Nevertheless, we were able to project a large number of genes (>11,000) also from the more distantly related *B. mori*. To supplement genes predicted by homology-based approaches, we additionally aligned proteins and CDS from LepBase [18]. In the absence of available RNA-seq data of *H. vespertilio*, we used RNA-seq data from the related *H. euphorbiae* species to obtain transcriptomic evidence for gene models. Finally, because homology and transcriptomic evidence may miss lineage-specific or low-expression genes, we aimed at increasing gene annotation completeness by using 4 *ab initio* gene prediction methods, aided by a large training set available from our high-quality gene projections. After integrating and filtering all evidence, this strategy produced a gene annotation with a higher BUSCO gene completeness than for other Bombycoidea species. Because all employed methods are usable for other species, our integrative gene annotation strategy likely has general applicability to many other genomes.

**Figure 5:**
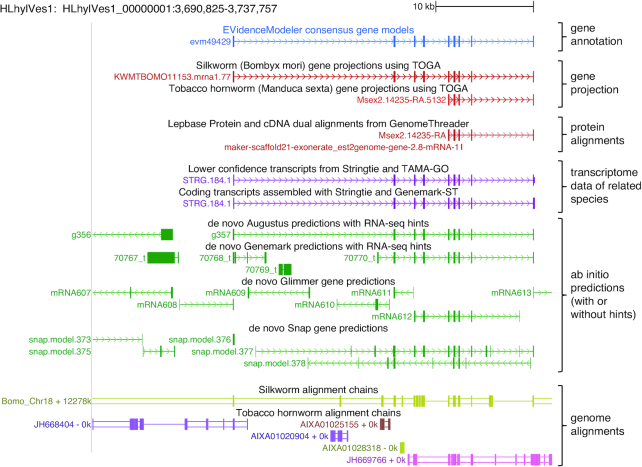
Genome browser visualization of annotations generated for the *H. vespertilio* genome. A University of California Santa Cruz (UCSC) genome browser instance visualizes the final gene annotation (blue), together with the underlying gene evidence, and pairwise genome alignment chains to *B. mori* and *M. sexta*.

To make our data accessible to the community and enable efficient use of the *H. vespertilio* genome for future studies, we provide a freely available genome browser instance [11] for data visualization and exploration. The genome browser visualization of the alignments to *B. mori* and *M. sexta*, the gene annotation, and the underlying gene evidence is shown in Fig. 5 for an exemplary genomic locus.

The *H. vespertilio* genome provides a valuable molecular resource to study speciation and hybridization processes in this genus. In particular, together with newly generated molecular data, the genome will help to infer the phylogeny of the 32 recognized species, which is not yet resolved owing to a high degree of hybridization between species and incomplete lineage sorting [26]. Furthermore, *Hyles* species exhibit a spectacular diversity of color patterns, exemplified by many different colorations of larva and adult wings (e.g., [27]) and are adapted to different ecological niches. Thus, the genome of *H. vespertilio* will facilitate a multitude of studies ranging from the genetic basis of morphological evolution and ecological adaptation to fundamental evolutionary processes such as speciation and hybridization.

## Methods

### Ethics, consent, and permissions

The DNA sample was derived from a single male individual of *Hyles vespertilio*, collected by Alberto Zilli in Latium (Province Rieti), Mt. Terminillo, Vallonina (950 m), Italy, on 31 May 2018 (specimen accession number LG2117), in accordance with the EU's environmental and scientific legislation. A second co-captured male was deposited as a voucher in the Museum of Zoology (Senckenberg Natural History Collections Dresden; with the DNA/tissue voucher number MTD-TW-12562).

### DNA/RNA extraction, library preparation, and sequencing

High molecular weight genomic DNA for the PacBio library was isolated after lysis of the liquid N_2_–ground abdomen in home-made lysis buffer (400 mM NaCl, 20 mM Tris base pH 8.0, 30 mM EDTA pH 8.0, 0.5% sodium dodecyl sulfate, 100 ug/mL Proteinase K) and standard phenol-chloroform extraction. High molecular weight genomic DNA was precipitated by centrifugation after adding ice-cold ethanol and dissolved in Tris-EDTA, pH 8.0. RNA was removed by RNase A treatment. Pulsed-field gel electrophoresis (SAGE Pippin Pulse, Sage Science Inc., Berverly, MA, USA) showed that the resulting DNA molecules were ~50–150 kb long. The genomic DNA concentration was 413 ng/μL (Qubit ds BR assay kit, Thermo Fisher Scientific, Waltham, MA, USA).

PacBio continuous long-read libraries were prepared as described in the "Guidelines for preparing size-selected 20 kb SMRTbell^TM^ templates" [28]. In brief, long genomic DNA was sheared to 60 kb by the Megaruptor^TM^ device (Diagenode). PacBio SMRTbell^TM^ libraries were size selected for fragments >20 kb with the SAGE BluePippin^TM^ device. Single-molecule real-time (SMRT) sequencing was performed on the SEQUEL system making use of sequencing chemistry 2.1; movie time was 10 hours for all SMRT cells. A total of 6 SMRT cells were sequenced with an average unique molecular yield of 5.3 Gb.

Total RNA was isolated from *H. euphorbiae* larval tissue samples (from Thalmässing-Waizenhofen, Germany) using the Direct-Zol RNA kit according to the manufacturer's instructions (Zymo Research, Irvine, CA, USA). RNA quantity was determined using an Implen Nanophotometer and integrity of RNA samples was verified using an Agilent 2100 Bioanalyzer and the RNA 6000 Nano Kit (Agilent Technologies, Santa Clara, CA, USA). Transcriptome sequencing was carried out by GATC Biotech on an Illumina HiSeq2500 Genome Analyzer using paired-end (2 x 125 bp) read technology. Sequencing libraries were generated from mRNA fragmented to an average of 180 bp using the TruSeq RNA library preparation kit (Illumina, San Diego, CA, USA) yielding ~12-20 million read pairs for each sample.

### Genome assembly

*De novo* genome assembly was performed with DAmar [29]. This assembler is based on an improved MARVEL assembler [12, 13] and integrates parts from the Dazzler suite (Supplementary Text 1) and DACCORD [30, 31].

To assemble the genome, we performed the following 4 steps: set-up, read patching, assembly, and error polishing. In the set-up phase, PacBio reads were filtered by choosing only the longest read of each zero-mode waveguide and requiring subsequently a minimum read length of 4 kb. The resulting 2.2 million reads (45× coverage) were stored in a Dazzler database [32].

The patch phase detects and corrects read artefacts including missed adapters, polymerase strand jumps, chimeric reads, and long low-quality read segments that are the primary impediments to long contiguous assemblies. To this end, we first computed local alignments of all raw reads. Because local alignment computation is by far the most time- and storage-consuming part of the pipeline, we reduced runtime and storage by masking repeats in the reads as follows. First, low-complexity intervals, such as microsatellites or homopolymers, were masked with DBdust [32]. Second, tandem repeats were masked by using datander and TANmask [33]. Third, we used a read alignment step to detect repeats (Supplementary Text 2). To this end, we first split all reads into groups representing 1× read coverage. For each group, we then aligned all reads against all others in the same group with daligner [34, 35] and masked all local regions in each read where ≥10 other reads aligned. The repeat masks were subsequently used to prevent *k*-mer seeding in repetitive regions when computing all local alignments between all reads. Because masking repeats can lead to missing low-quality or noisy regions within PacBio reads, we used LAseparate to find proper alignment chains that prematurely end in repeat regions. For those alignment chains, we recomputed local alignments with the repcomp tool without using the repeat mask. Then we applied LAfix to detect and correct read artefacts.

Manual inspection of the overlap graph (Supplementary Fig. 1) revealed that chimeric reads were passed on to the assembly phase because chimeric breaks within large repeat regions were missed. Therefore, we improved the detection of chimeric reads by re-analysing repetitive regions up to a length of 8 kb for chimers. Any subread that includes a repetitive region that could not be spanned by ≥3 proper alignment chains was excluded. This additional step led to a final overlap graph that was much cleaner, which made manual validation easier (Supplementary Fig. 2).

In the assembly phase, we first calculated all overlaps between patched reads using the same alignment strategy of the patch phase. The subsequent steps of (i) computing a quality track for all reads, (ii) computing a detailed repeat mask, (iii) filtering overlap piles, (iv) computing the overlap graph, and (v) touring the overlap graph to obtain primary contigs follow the steps of the original MARVEL assembly pipeline [12, 13].

In the error-polishing phase, we polished all contigs using the raw PacBio reads and 2 rounds of Arrow [36]. All commands and parameters of all steps in the assembly are provided in Supplementary Data Files 1–3.

To assess completeness of the genome assembly, we used BUSCO v3 [14] and the Endopterygota dataset comprising 2,442 genes. To assess potential contamination, we used BlobTools [37] with default parameters except “max_target_seqs 10” in the blastn step and the NCBI nt database (31 October 2019) for the taxonomy classification step. As shown in Supplementary Table 4, BlobTools classified only 2.17 Mb (0.33% of the 651.4-Mb assembly) as contamination, showing that contamination is not a major issue that could explain the genome size expansion compared to other Bombycoidea.

To investigate whether some contigs may represent alternative haplotypes, we determined and plotted the per base read coverage. As shown in Supplementary Fig. 3, this revealed a large peak at ~40×, which is consistent with our sequencing coverage, and a small hump at ~20×, indicating that some contigs may be alternative haplotypes. Therefore, we used purge_dups [38] to detect alternative haplotypes based on read coverage. This tool assigned 622.7 Mb (95.6% of the 651.4 Mb assembly) as the purged primary assembly and assigned 28.7 Mb (4.4% of the assembly) as alternative haplotypes (Supplementary Table 5). Haplotig contigs are mostly small and often repeat-rich, which complicates accurate read coverage determination because unique read mappings are harder to obtain. Furthermore, repeating the BUSCO analysis on the purged primary assembly resulted in a smaller percentage of complete but duplicated genes (0.3% vs 2.8%) but also a 0.5% decrease in the total number of complete BUSCO genes. This suggests that while some contigs are indeed alternative haplotypes, others contain unique genes and should not be classified as alternative haplotypes. Overall, this analysis shows that alternative haplotypes cannot explain the larger genome size of *H. vespertilio* compared to other Bombycoidea.

To assemble the mitochondrial genome, the corresponding PacBio reads were extracted by mapping them with daligner to the mitochondrial reference sequence of the related *Ampelophaga rubiginosa* (NCBI Accession No. NC_035431.1; Liu, Q.-N.). The resulting overlaps were filtered for proper circular alignment chains (chain lengths 4–14 kb, maximum unaligned bases 1,500) with the tool LAfilterMito. The filtered reads were then processed according to the general assembly pipeline (read patching, assembly, error polishing). After read patching, the reads were split into shorter reads with a 1,500-bp overlap to ensure that the assembly creates a circular contig that consist of >1 read. Error polishing was done by running Arrow [36] with filtered PacBio reads. We used Circlator [39] to circularize the mitochondrial contig, map it to itself, and trim back the overlapping part.

### Repeat annotation

We first used RepeatModeler [40] (with parameters “*-*engine ncbi”) to identify repeat families in genomes of *H. vespertilio, B. mori*, and *M. sexta*. We used RepeatMasker with default parameters to soft-mask the 3 genomes with their respective repeat library. Tandem Repeat Finder [41] was used to detect simple and tandem repeats.

### Genome alignment

The *H. vespertilio* genome was aligned to the genomes of *M. sexta* (Sphingidae) and *B. mori* (Saturniidae; sequence data were downloaded from LepBase [18, 42]). Pairwise genome alignments were produced using lastz [43] with parameters K = 2,400, L = 3,000 and the default scoring matrix, axtChain [44], chainCleaner [45], and RepeatFiller [46] (all with default parameters).

### Gene annotation

Consensus gene models were produced from gene projections, protein homology, transcriptome data, and *ab initio* predictions. Evidence was ranked and weighted following the guidelines of the EVM manual [47]. The *ab initio* predictors were given the lowest rank, followed by the spliced alignments. Because transcript assembly was performed using data from another species, this was ranked second after the gene projections.

As the first piece of evidence, we used TOGA (last commit: 2 May 2019) to project annotations of coding genes from multiple reference genomes to a query genome. Briefly, TOGA takes as input pairwise genome alignment chains between a designated reference (here *B. mori* or *M. sexta*) and query genome (here *H. vespertilio*), coding transcript annotations for the reference species and a file linking gene and transcripts isoforms. For each gene, TOGA identifies the chain(s) that aligns the putative ortholog in the query using synteny and the amount of aligning exonic and intronic sequence. To obtain the locations of coding exons of this gene, TOGA then extracts the genomic region corresponding to the gene on this chain from the query assembly and uses CESAR 2.0 [17] in multi-exon mode. *B. mori* gene models from SilkBase [48] and *M. sexta* models from LepBase [18] were projected to *H. vespertilio*. Using a 10% overlap, 15,169 (77%) of the 19,760 *M. sexta* projections overlap a *B. mori* projection and 9,996 (85%) of the 11,721 *B. mori* projections overlap an *M. sexta* projection. A total of 3,336 of the *M. sexta* and *B. mori* projections are identical. Projected genes were assigned a weight of 8 and classed as “Other prediction” within EVM.

As the second piece of evidence, CDS from 22 available lepidopteran species were downloaded from LepBase [18]. CDS were translated to corresponding peptide sequence using Prank (v.170427) [49]. CDS and peptide sequences were co-aligned to the assembly of *H. vespertilio* using GenomeThreader [50] with the parameters “-gcmincoverage 70 -paralogs -species drosophila**.”** Species used and number of significant alignments are detailed in Supplementary Table 3. Alignments were passed to EVM as “Protein” alignments and assigned a weight of 4.

As a third piece of evidence, we used short-read RNA-seq data that were generated from larval tissue of the closely related *H. euphorbiae*, because RNA-seq data of *H. vespertilio* were not available. Reads were mapped to the *H. vespertilio* assembly using hisat2 (v 2.0.0) with parameters “–dta –no-unal –mp 4,1 –score-min L,0,-0.125,” which resulted in mapping 65.82% of the reads. Transcripts were assembled with StringTie [20] with default parameters. Fasta sequences for each transcript were extracted using bedtools. ORFs were predicted for each transcript using GenemarkS-T (v5.1) [21] with parameters “–strand direct.” GenemarkS-T transcripts were given a weight of 7 and classed as “Other prediction” within EVM. TAMA [51] was also used to identify ORFs within assembled transcripts. Briefly, ORFs are predicted from all forward frames of a transcript. Predicted peptide sequences are then queried using Blastp [52] against a BLAST database of the downloaded LepBase proteins and further classified as full-length or partial hits. The highest-scoring ORF for each transcript is mapped back to the transcript, and putative nonsense-mediated decay (NMD) targets were determined and excluded. Full-length and non-NMD target transcripts were provided to EVM as class “Other prediction” with a weight of 7. The remaining transcripts were provided to EVM as “Transcript” alignments with a weight of 4.

As a fourth piece of evidence, 4 *ab initio* gene prediction tools were used to predict genes in the *H. vespertilio* genome. As training data, we used a set of non-overlapping, full-length and intact genes that were projected from *B. mori* and *M. sexta* and resulted in an identical gene model in *H. vespertilio* (2,504 genes). This set was randomly divided into 80% training data and 20% test data. SNAP [22] and GlimmerHMM [23] were trained in accordance with the available manuals, and genes were predicted. To run Augustus [24], we first mapped the RNA-seq data again to the genome with using hisat2 and strict mapping parameters (–no-mixed –no-discordant –dta –no-unal –n-ceil L,0,0.05; read mapping rate of 46.79%) to generate hints. Intron positions as predicted by spliced alignments were extracted using the bam2hints module from Augustus (v3.3.1). The heliconius_melpomene1 model provided with Augustus was optimized for *H. vespertilio* using optimize_augustus.pl (–cpus = 12 –kfold = 12) and the training gene set, and Augustus was further trained using these parameters with the etraining tool. Genes were then predicted with Augustus, providing the intron positions as extrinsic hints. Finally, intron hints were provided to Genemark-ES [25] for self-training and gene prediction. Gene predictions were evaluated against the test gene set using ParsEval [53]. SNAP and GlimmerHMM were subsequently given a weight of 1, while Augustus and Genemark-ES predictions were given a weight of 2. All were provided as type “*ab initio* prediction.”

EVM [15] was then run using the above evidence and described weights. Full-length, functional TOGA projections from *M. sexta* and *B. mori* with no CDS overlap to any consensus model were included into the consensus set. Consensus gene models with >50% CDS overlap within a single repeat region, as annotated by RepeatMasker, were removed. To assess completeness of the gene annotation, we applied BUSCO v3 [14] in protein mode to our final *H. vespertilio* protein set and the annotated *B. mori* and *M. sexta* proteins, using the Endopterygota dataset comprising 2,442 genes.

## Availability of Supporting Data and Materials

All raw sequencing data and the genome assembly of *H. vespertilio* are available at the NCBI under the Bioproject ID PRJNA574010. *H. euphorbiae* RNA-seq data have been submitted to the EBI SRA (accession numbers: ERS4198286–ERS4198293). The genome, our gene annotations including the gene evidences, and genome alignments to *B. mori* and *M. sexta* are available for download [54] and for genome browser visualization and exploration [55]. Other data supporting this work are openly available in the *GigaScience* repository, GigaDB [56].

## Additional Files

Supplementary Table 1: BUSCO statistics for 3 Lepidoptera genomes

Supplementary Table 2: Repeat content

Supplementary Table 3: Number of alignments produced using GenomeThreader from 22 Lepbase species

Supplementary Table 4: Contamination analysis

Supplementary Table 5: purge_dups classification of contigs in the *Hyles* assembly

Supplementary Figure 1: Final overlap graph without the additional chimeric read removal step

Supplementary Figure 2: Final overlap graph after applying the additional chimeric read removal step

Supplementary Figure 3: Per base read coverage histogram

Supplementary Text 1: DALIGNER - Fast and sensitive detection of all pairwise local alignments

Supplementary Text 2: Detecting and masking repeats

Supplementary Data File 1: Instructions to *Hyles* assembly

Supplementary Data File 2: DAmar coverage estimation configuration file

Supplementary Data File 3: DAmar assembly configuration file

giaa001_GIGA-D-19-00361_Original_Submission

giaa001_GIGA-D-19-00361_Revision_1

giaa001_Response_to_Reviewer_Comments_Original_Submission

giaa001_Reviewer_1_Report_Original_Submission Reuben William Nowell, Ph.D. -- 11/8/2019 Reviewed

giaa001_Reviewer_2_Report_Original_Submission Simon Baxter -- 11/23/2019 Reviewed

giaa001_Supplemental_Files

## Abbreviations

BLAST: Basic Local Alignment Search Tool; bp: base pairs; BUSCO: Benchmarking Universal Single-Copy Orthologs; CESAR: Coding Exon-Structure Aware Realigner; CDS: coding sequence; EDTA: ethylenediaminetetraacetic acid; EVM: Evidence Modeler; Gb: gibabase pairs; HMM: hidden Markov model; kb: kilobase pairs; LINE: long interspersed nuclear element; Mb: megabase pairs; NCBI: National Center for Biotechnology Information; NMD: nonsense-mediated decay; ORF: open reading frame; PacBio: Pacific Biosciences; RNA-seq: RNA sequencing; SINE: short interspersed nuclear element; SMRT: single-molecule real-time; SNAP: Semi-HMM-based Nucleic Acid Parser; SRA: Sequence Read Archive; TAMA: Transcriptome Annotation by Modular Algorithms; TOGA: Tool to infer Orthologs from Genome Alignments.

## Competing Interests

The authors declare that they have no competing interests.

## Funding

This work was funded by the Max Planck Gesellschaft (M.H., G.M.), the Federal Ministry of Education and Research (grant 01IS18026C), and the German Research Foundation (grants HI 1423/3-1, HU 1561/5-1, and RE 603/25-1). It benefitted from the sharing of expertise within the DFG priority program SPP 1991 Taxon-Omics.

**Author contributions:**

GM, AKH and MH conceived the study. MP assembled the genome and mitogenome. DJ aligned genomes, analysed transposons and annotated genes. FP contributed to material sampling and data analysis. SW extracted gDNA and sequenced the genome. HV sequenced and provided RNA-seq data. GM designed the sequencing and assembly strategy. MH contributed to data analysis and set up the genome browser. MP, DJ, AKH and MH made figures. GM and AKH provided funding for DNA sequencing. AKH and MH wrote the manuscript with input from all authors.
