# Supplementary material for: A highly contiguous genome assembly of the bat hawkmoth Hyles vespertilio (Lepidoptera: Sphingidae)
Source: Gigascience. 2020 Jan 23;9(1):giaa001. doi: 10.1093/gigascience/giaa001 (PMC6977585; doi:10.1093/gigascience/giaa001)
Supplement: giaa001_GIGA-D-19-00361_Original_Submission [file giaa001_giga-d-19-00361_original_submission.pdf]

GigaScience

Data Note A highly contiguous genome assembly of the bat hawkmoth Hyles vespertilio (Lepidoptera: Sphingidae)

--Manuscript Draft--

**Manuscript Number:**	GIGA-D-19-00361
**Full Title:**	Data Note A highly contiguous genome assembly of the bat hawkmoth Hyles vespertilio (Lepidoptera: Sphingidae)
**Article Type:**	Data Note
**Funding Information:**	Max-Planck-Gesellschaft (-) — Dr. Gene Myers<br>Deutsche Forschungsgemeinschaft (HU 1561/5-1 & RE 603/25-1) — Dr. Anna K. Hundsdoerfer
**Abstract:**	Adapted to different ecological niches, moth species belonging to the Hyles genus exhibit a spectacular diversity of larval color patterns. These species diverged about 7.5 Mya, making this rather young genus an interesting system to study a wide range of questions including the process of speciation, ecological adaptation and adaptive radiation. Here we present a high-quality genome assembly of the bat hawkmoth Hyles vespertilio , the first reference genome of a member of the Hyles genus. We generated 51X PacBio long reads with an average read length of 8.9 kb. PacBio reads longer than 4 kb were assembled into contigs, resulting in a 651.4 Mb assembly consisting of 530 contigs with an N50 value of 7.5 Mb. The circular mitochondrial contig has a length of 15,303 bases. The H. vespertilio genome is very repeat-rich and exhibits a higher repeat content (50.3%) than other Bombycoidea species such as Bombyx mori (45.7%) and Manduca sexta (27.5%). We developed a comprehensive gene annotation workflow to obtain consensus gene models from different evidences including gene projections, protein homology, transcriptome data, and ab initio predictions. The resulting gene annotation is highly complete with 94.5% of BUSCO genes being completely present, which is higher than the BUSCO completeness of B. mori (92.2%) and M. sexta (90%). Our gene annotation strategy has general applicability to other genomes and the H. vespertilio genome provides a valuable molecular resource to study a range of questions in this genus, including phylogeny, incomplete lineage sorting, speciation and hybridization. A genome browser displaying the genome, alignments and annotations is available at https://genome-public.pks.mpg.de/cgi-bin/hgTracks?db=HLhylVes1 .
**Corresponding Author:**	Michael Hiller<br><br>GERMANY
**Corresponding Author Secondary Information:**	
**Corresponding Author's Institution:**	
**Corresponding Author's Secondary Institution:**	
**First Author:**	Martin Pippel
**First Author Secondary Information:**	
**Order of Authors:**	Martin Pippel<br>David Jebb<br>Franziska Patzold<br>Sylke Winkler<br>Gene Myers<br>Michael Hiller<br>Anna K. Hundsdoerfer

Powered by Editorial Manager® and ProduXion Manager® from Aries Systems Corporation

Order of Authors Secondary Information:	
Additional Information:	
Question	Response
Are you submitting this manuscript to a special series or article collection?	No
**Experimental design and statistics**<br><br>Full details of the experimental design and statistical methods used should be given in the Methods section, as detailed in our Minimum Standards Reporting Checklist. Information essential to interpreting the data presented should be made available in the figure legends.<br><br>Have you included all the information requested in your manuscript?	No
If not, please give reasons for any omissions below.<br><br>as follow-up to "**Experimental design and statistics**<br><br>Full details of the experimental design and statistical methods used should be given in the Methods section, as detailed in our Minimum Standards Reporting Checklist. Information essential to interpreting the data presented should be made available in the figure legends.<br><br>Have you included all the information requested in your manuscript?<br><br>"	not applicable as no experiments were performed
**Resources**<br><br>A description of all resources used, including antibodies, cell lines, animals and software tools, with enough information to allow them to be uniquely identified, should be included in the Methods section. Authors are strongly	Yes

Powered by Editorial Manager® and ProduXion Manager® from Aries Systems Corporation

encouraged to cite Research Resource Identifiers (RRIDs) for antibodies, model organisms and tools, where possible.<br><br>Have you included the information requested as detailed in our Minimum Standards Reporting Checklist?	
**Availability of data and materials**<br><br>All datasets and code on which the conclusions of the paper rely must be either included in your submission or deposited in publicly available repositories (where available and ethically appropriate), referencing such data using a unique identifier in the references and in the “Availability of Data and Materials” section of your manuscript.<br><br>Have you have met the above requirement as detailed in our Minimum Standards Reporting Checklist?	Yes

Powered by Editorial Manager® and ProduXion Manager® from Aries Systems Corporation

Manuscript Click here to download Manuscript Manuscript.docx

DATA NOTE

A highly contiguous genome assembly of the bat hawkmoth *Hyles vespertilio* (Lepidoptera: Sphingidae)

Martin Pippel [1, 2, a], David Jebb [1, 2, 3, a], Franziska Patzold [4], Sylke Winkler [1], Gene Myers [1], Michael Hiller [1, 2, 3, #] Anna K. Hundsdoerfer [4, #]

[1] Max Planck Institute of Molecular Cell Biology and Genetics, Pfotenhauerstraße 108, 01307 Dresden, Germany

[2] Center for Systems Biology Dresden, Pfotenhauerstr. 108, 01307, Dresden, Germany

[3] Max Planck Institute for the Physics of Complex Systems, Nöthnitzer Str. 38, 01187, Dresden, Germany

[4] Senckenberg Natural History Collections Dresden, Königsbrücker Landstr. 159, 01109 Dresden, Germany

[a] Joint first authorship

[#] Corresponding authors:
hiller@mpi-cbg.de & Anna.Hundsdoerfer@senckenberg.de

Abstract

Adapted to different ecological niches, moth species belonging to the *Hyles* genus exhibit a spectacular diversity of larval color patterns. These species diverged about 7.5 Mya, making this rather young genus an interesting system to study a wide range of questions including the process of speciation, ecological adaptation and adaptive radiation. Here we present a high-quality genome assembly of the bat hawkmoth *Hyles vespertilio*, the first reference genome of a member of the *Hyles* genus. We generated 51X PacBio long reads with an average read length of 8.9 kb. PacBio reads longer than 4 kb were assembled into contigs, resulting in a 651.4 Mb assembly consisting of 530 contigs with an N50 value of 7.5 Mb. The circular mitochondrial contig has a length of 15,303 bases. The *H. vespertilio* genome is very repeat-rich and exhibits a higher repeat content (50.3%) than other Bombycoidea species such as *Bombyx mori* (45.7%) and *Manduca sexta* (27.5%). We developed a comprehensive gene annotation workflow to obtain consensus gene models from different evidences including gene projections, protein homology, transcriptome data, and *ab initio* predictions. The resulting gene annotation is highly complete with 94.5% of BUSCO genes being completely present, which is higher than the BUSCO completeness of *B. mori* (92.2%) and *M. sexta* (90%). Our gene annotation strategy has general applicability to other genomes and the *H. vespertilio* genome provides a valuable molecular resource to study a range of questions in this genus, including phylogeny, incomplete lineage sorting, speciation and hybridization. A genome browser displaying the genome, alignments and annotations is available at https://genome-public.pks.mpg.de/cgi-bin/hgTracks?db=HLhylVes1.

Keywords

Genome assembly, PacBio long reads, hawkmoth - silk moth comparison, comprehensive gene annotation

INTRODUCTION

Bombycoidea are a speciose superfamily of moths, comprising ten families, more than 500 genera [1] and 6,092 species that are mostly diversified in the intertropical region of the globe [2]. This superfamily includes the two well-known macrolepidoptera families Saturniidae and Sphingidae. The larvae of at least eight Saturniid species are eaten as an important source of proteins in rural Africa [3]. Sphingids are large (4 to over 10 cm wingspan) and thus strong insects with excellent flying abilities and thereby pollinators that are important prey for bats and some species can produce ultrasound to divert attacks by echolocating bats [4]. Furthermore, Bombycoidea not only comprises some of the largest moth species, exemplified by the giant silk moth *Attacus atlas* with a wingspan measuring 25-30 cm, but also includes several model organisms, such as the domestic silkmoth *Bombyx mori*, a saturniid of great economic importance for silk production, and the tobacco hornworm *Manduca sexta*, which is a common pest sphingid species causing considerable damage to tobacco, tomato, pepper, eggplant and plantations of other crops [5]. Since these species have been extensively studied, they take a leading role in the fields of Lepidoptera genetics and physiology. To date, genomes of only four Bombycoidea species have been published (*Bombyx mori* [6] together with its two closely-related congeners *B. huttoni* and *B. mandarina* of the Saturniidae, and *Manduca sexta* [7] of the Sphingidae), which represents a tiny fraction of the diversity of Bombycoidea.

Sphingidae include the hawkmoth genus *Hyles*. This genus originated in South America and comprises 32 recognized species [8-10] with representatives native to all continents and major islands (except Antarctica). As a rather young genus, estimated to have diverged about 7.5 Mya [4], species from different continents are still able to hybridize. This makes *Hyles* an interesting genus to study a wide range of questions including the process of speciation, ecological adaptation, adaptive radiation, genetics of reproduction and evolution in action. However, such studies are hampered by the lack of suitable molecular resources. In particular, a well-assembled reference genome of any *Hyles* species is currently missing.

Here we present a high-quality nuclear and mitochondrial genome assembly of the bat hawkmoth *Hyles vespertilio* (Figure 1), the first reference genome assembly of a member of the *Hyles* genus. Compared with other members of the *Hyles* genus that have broad species distributions, *H. vespertilio* is restricted to mountainous river valleys in the Western Palearctic. This rather restricted distribution is likely associated with a lower degree of hybridization, which makes *H. vespertilio* a good target for assembly of a reference genome. In the following, we report the assembly of the *H. vespertilio* genome from using PacBio long sequencing reads, the annotation of this genome and a comparative analysis to *B. mori* and *M. sexta*. All data can be visualized and downloaded from a genome browser instance at https://genome-public.pks.mpg.de/cgi-bin/hgTracks?db=HLhylVes1.

RESULTS

Assembly of Hyles vespertilio from long sequencing reads

We generated 51X PacBio long reads with an average read length of 8.9 kb and an N50 read length of 16.5 kb. PacBio reads longer than 4 kb were assembled into contigs with a customized assembler called DAmar. DAmar is a hybrid of our MARVEL approach, which was used to assemble the Axolotl [11] and *Schmidtea* [12] genomes, and the Dazzler method (https://dazzlerblog.wordpress.com/). This resulted in a 651.4 Mb assembly consisting of 530 contigs. The contig N50 value of 7.5 Mb (Figure 2A).

Compared with the assembly of *B. mori* (460.3 Mb) and *M. sexta* (419.4 Mb), the *H. vespertilio* assembly is substantially larger by 191 and 232 Mb, respectively. As shown in Figure 2, our contigs are >100 times longer than the contigs of the *M. sexta* assembly (N50 value 52 kb) but shorter than the contigs of the *B. mori* assembly (N50 value 12.2 Mb), which was based on a

combination of different sequencing technologies including 80X PacBio long reads, 60X Illumina reads and complete sequences of BAC and Fosmid clones.

We also assembled the mitochondrial genome of *H. vespertilio* using PacBio long reads and the DAmar assembler. The resulting circular mitochondrial contig has a length of 15,303 bases.

Repeat content

To assess to which extent the *H. vespertilio* assembly consist of repetitive sequences, we modelled and masked repeats using RepeatModeler and RepeatMasker. To compare repeat content with *B. mori* and *M. sexta*, we applied the same procedure to these genomes as well. This analysis shows that the *H. vespertilio* genome has a high repeat content of 50.3% (Figure 3, Supplementary Table 1). *B. mori* also has a repeat-rich genome (45.7%), while the *M. sexta* assembly is less repeat-rich (27.5%), which may be an underestimation as similar repeat copies may not be properly assembled for this species. Long Interspersed Nuclear Elements (LINEs) comprise the largest repeat class in the *H. vespertilio* assembly, followed by Short Interspersed Nuclear Elements (SINEs) and DNA transposons (Figure 3). This is similar to *B. mori* and *M. sexta*, except that the latter exhibits much fewer LINEs.

Gene annotation

To annotate genes in the *H. vespertilio* assembly, we used Evidence Modeler [13] to produce consensus gene models from multiple sources of predictions. A flowchart visualizing the gene annotation strategy is shown in Figure 4. First we generated pairwise genome alignments between the *H. vespertilio* assembly and *B. mori* and *M. sexta*, and used these alignments to project annotated genes from *B. mori* and *M. sexta* to the *H. vespertilio* genome using CESAR 2.0 [14]. This resulted in 11,721 and 19,760 gene predictions that were projected from *B. mori* and *M. sexta*, respectively. Second, RNA-seq data from *H. euphorbiae* was mapped to the *H. vespertilio* assembly and assembled into

transcripts using Stringtie [15]. TAMA and GenemarkS-T [16] were used to predict open reading frames in assembled transcripts, predicting 45,488 and 19,776 coding transcripts respectively. Third, coding sequences (CDS) from 22 available Lepidopteran species were downloaded from Lepbase. CDS and translated protein sequences were aligned to the *H. vespertilio* assembly using GenomeThreader [17]. The number of significant alignments varied markedly between species, ranging from 2,207 to 154,299 (Supplementary Table 2). Finally, four *ab initio* gene finders, SNAP, glimmerHMM, Genemark-ES and Augustus [18-21], predicted 67,351, 108,570, 92,757 and 61,225 gene models respectively. All evidences were passed to Evidence Modeler to produce a consensus gene set. Likely missing genes were recovered by keeping the *M. sexta* and *B. mori* CESAR 2.0 projections, generating a set of 50612 loci. Finally, models for which greater than 50% of the coding sequence was contained within repeat sequence were removed. This produced a final set of 23,768 genes.

To assess the completeness of our *H. vespertilio* gene annotation, we used BUSCO [22] and the set of conserved, single-copy Endopterygota genes. We found that our annotation is highly complete with 94.5% of these BUSCO genes being completely present. Importantly, this completeness is higher than the gene annotations of both *B. mori* (92.2% complete BUSCO genes) and *M. sexta* (90% complete BUSCO genes).

DISCUSSION

Here we present a high-quality genome assembly for the bat hawkmoth *Hyles vespertilio*, the fifth Bombycoidea species (and third genus of the superfamily) with a sequenced genome. Our assembly is based on PacBio long reads, which have been instrumental to assemble long contigs, in particular since the *H. vespertilio* genome is substantially longer and more repeat-rich than other Bombycoidea species. Our final assembly comprises 530 contigs with an N50 value of 7.5 Mb, making it the second-most contiguous genome of any Bombycoidea species to date.

To annotate coding genes, we developed a strategy that integrates multiple different gene evidences. First, we used genome alignments to project genes annotated in related species. Gene projection generally produces very accurate annotations, but is by definition limited by the completeness of the gene sets of related species. Evolutionary distance is another factor influencing the completeness of results obtained from gene projections, with closely-related species generally allowing for more complete projections, consistent with our result that substantially more genes were projected using *M. sexta* as a reference. Nevertheless, we were able to project a large number of genes (>11000) also from the more distantly-related *B. mori*. To supplement genes predicted by homology-based approaches, we additionally aligning proteins and CDS from Lepbase. In the absence of available RNA-seq data of *H. vespertilio*, we used RNA-seq data from the related *Hyles euphorbiae* species to obtain transcriptomic evidence for gene models. Finally, since homology and transcriptomic evidence may miss lineage-specific or lowly-expressed genes, we aimed at increasing gene annotation completeness by employing four *ab initio* gene prediction methods, aided by a large training set available from our high-quality gene projections. After integrating and filtering all evidences, this strategy produced a gene annotation with a higher BUSCO gene completeness than for other Bombycoidea species. Since all employed methods are useable for other species, our integrative gene annotation strategy likely has general applicability to many other genomes.

The *H. vespertilio* genome provides a valuable molecular resource to study speciation and hybridization processes in this genus. In particular, together with newly generated molecular data, the genome will help to infer the phylogeny of the 32 recognized species, which is not yet resolved due to a high degree of hybridization between species and incomplete lineage sorting [23]. Furthermore, *Hyles* species exhibit a spectacular diversity of color patterns, exemplified by many different colorations of larva and adult wings, e.g. [24], and are adapted to different ecological niches. Thus, the genome of *H. vespertilio* will facilitate a multitude of studies ranging from the genetic basis of morphological evolution, ecological adaptation to fundamental evolutionary processes such as speciation and hybridization.

To make our data accessible to the community and enable efficient use of the *H. vespertilio* genome for future studies, we provide a freely-available genome browser instance (https://genome-public.pks.mpg.de/cgi-bin/hgTracks?db=HLhylVes1) for data visualization and exploration. The genome browser visualization of the alignments to *B. mori* and *M. sexta*, the gene annotation, and the underlying gene evidences is shown in Figure 5 for an exemplary genomic locus.

METHODS

Ethics, consent, and permissions

The DNA sample was derived from a single male individual of *Hyles vespertilio*, collected by Alberto Zilli in Latium (Province Rieti), Mt. Terminillo, Vallonina (950m), Italy, on 31.V.2018 (specimen accession number LG2117), in accordance with the EU's environmental and scientific legislation. A second co-captured male was deposited as a voucher in the Museum of Zoology (Senckenberg Natural History Collections Dresden; with the DNA/tissue voucher number MTD-TW-12562).

DNA extraction, library preparation and sequencing

High molecular weight (HMW) genomic DNA for the PacBio library was isolated after lysis of the liquid N_2-ground abdomen in home-made lysis buffer (400 mM NaCl, 20 mM Tris base pH 8.0. 30 mM EDTA pH 8.0, 0,5% SDS, 100 ug/ml Proteinase K) and standard phenol-chloroform extraction. HMW genomic DNA was precipitated by centrifugation after adding ice-cold ethanol and dissolved in Tris-EDTA, pH 8.0. RNA was removed by RNase A treatment. Pulse field gel electrophoresis (SAGE Pippin Pulse) showed that the resulting DNA molecules were around 50 -150 Kb long. The gDNA concentration was 413 ng/µ (Qubit ds BR assay kit, Thermo Fisher Scientific).

Pacific Bioscience continuous long read (CLR) libraries have been prepared as described in the 'Guidelines for preparing size-selected 20 kb SMRTbell™ templates. In brief, long gDNA has been shared to 60 kb by the Megaruptor™ device (Diagenode). PacBio SMRTbell™ libraries have been size selected for fragments larger than 20 kb with the SAGE BluePippin™ devise. SMRT sequencing has been performed on the SEQUEL system making use of sequencing chemistry 2.1; movie time was 10 hours for all SMRT cells. A total of 6 SMRT cells have been sequenced with an average unique molecular yield of 5.3 Gb.

Genome assembly

De novo genome assembly was performed with DAmar (https://github.com/MartinPippel/DAmar). This assembler is based on an improved MARVEL assembler [11, 12] and integrates parts from the DAZZLER (https://dazzlerblog.wordpress.com/) and DACCORD [25, 26].

To assemble the genome, we performed the following four steps: setup, read patching, assembly and error polishing. In the setup phase, PacBio reads were filtered by choosing only the longest read of each zero-mode waveguide and requiring subsequently a minimum read length of 4 kb. The resulting 2.2 million reads (45X coverage) were stored in a DAZZLER database.

The patch phase detects and corrects read artefacts including missed adapters, polymerase strand jumps, chimeric reads and long low-quality read segments that are the primary impediments to long contiguous assemblies. To this end, we first computed local alignments of all raw reads. Since local alignment computation is by far the most time and storage consuming part of the pipeline, we reduced runtime and storage by masking repeats in the reads as follows. First, low complexity intervals, such as microsatellites or homopolymers, were masked with DBdust. Second, tandem repeats were masked by using datander and TANmask. Third, as described on https://dazzlerblog.wordpress.com/2016/04/01/detecting-and-soft-masking-

repeats/, we split all reads into groups representing 1X read coverage. For each group, we then aligned all reads against all others with daligner and masked all local regions in each read where at least 10 other reads aligned. The repeat masks were subsequently used to prevent k-mer seeding in repetitive regions when computing all local alignments between all reads. Since masking repeats can lead to missing low quality or noisy regions within PacBio reads, we used LAseparate to find proper alignment chains that prematurely end in repeat regions. For those alignment chains, we recomputed local alignments with the repcomp tool without using the repeat mask. Then we applied LAfix to detect and correct read artefacts.

Manual inspection of the overlap graph (Supplementary Figure 1) revealed that chimeric reads were passed on to the assembly phase because chimeric breaks within large repeat regions were missed. Therefore, we improved the detection of chimeric reads by re-analyzing repetitive regions up to a length of 8 kb for chimers. Any subread which includes a repetitive region that could not be spanned by at least three proper alignment chains was excluded. This additional step lead to a final overlap graph that was much cleaner, which made manual validation easier (Supplementary Figure 2).

In the assembly phase, we first calculated all overlaps between patched reads using the same alignment strategy of the patch phase. The subsequent steps of (i) computing a quality track for all reads, (ii) computing a detailed repeat mask, (iii) filtering overlap piles, (iv) computing the overlap graph, and (v) touring the overlap graph to obtain primary and alternative contigs follow the steps of the original MARVEL assembly pipeline [11, 12].

In the error polishing phase, we polished all contigs using the raw PacBio reads and two rounds of Arrow (https://github.com/PacificBiosciences/GenomicConsensus.git). All commands and parameters of all steps in the assembly are provided in Supplementary Data files 1-3.

To assemble the mitochondrial genome, the corresponding PacBio reads were extracted by mapping them with daligner to the mitochondrial reference sequence of the related *Ampelophaga rubiginosa* (NCBI Acc. No. NC_035431.1; unpublished). The resulting overlaps were filtered for proper circular alignment chains (chain lengths 4-14 kb, max unaligned bases 1500) with the tool LAfilterMito. The filtered reads were then processed according to the general assembly pipeline (read patching, assembly, error polishing). After read patching, the reads were split into shorter reads with a 1500 bp overlap to ensure that the assembly creates a circular contig that consist of more than a single read. Error polishing was done by running Arrow (https://github.com/PacificBiosciences/GenomicConsensus.git) with filtered PacBio reads. The mitochondrial contig was circularized, by mapping it to itself and trimming back the overlapping part.

Repeat annotation

We first used RepeatModeler (http://www.repeatmasker.org/) with parameters '*-engine ncbi*') to identify repeat families in genomes of *H. vespertilio*, *B. mori* and *M. sexta*. We used RepeatMasker with default parameters to soft-mask the three genomes with their respective repeat library. Tandem Repeat Finder (https://tandem.bu.edu/trf/trf.html) to detect simple and tandem repeats.

Genome alignment

The *H. vespertilio* genome was aligned to the genomes of *M. sexta* (Sphingidae) and *Bombyx mori* (Saturnidae; sequence data was downloaded from LepBase at http://ensembl.lepbase.org/index.html [27]). Pairwise genome alignments were produced using lastz [28] with parameters K = 2400, L = 3000 and the default scoring matrix, axtChain [29], chainCleaner [30] and chainGapFiller[31] (all with default parameters).

Gene annotation

Consensus gene models were produced from four types of gene predictions, gene projections, protein homology, transcriptome data, and *ab initio*

predictions. As the first evidence, we used TOGA (Tool to find Orthologs from Genome Alignments, last commit: 02/05/2019) to project annotations of coding genes from multiple reference genomes to a query genome. Briefly, TOGA takes as input pairwise genome alignment chains between a designated reference (here *B. mori* or *M. sexta*) and query genome (here *H. vespertilio*), coding transcript annotations for the reference species and a file linking gene and transcripts isoforms. For each gene, TOGA identifies the chain(s) that aligns the putative ortholog in the query using synteny and the amount of aligning exonic and intronic sequence. To obtain the locations of coding exons of this gene, TOGA then extracts the genomic region corresponding to the gene on this chain from the query assembly and uses CESAR 2.0 (Coding Exon Structure Aware Realigner) [14] in multi-exon mode. *Bombyx mori* gene models from http://silkbase.ab.a.u-tokyo.ac.jp/ and *Manduca sexta* models from Lepbase were projected to *H. vespertilio*. Projected genes were assigned a weight of 8 and classed as “Other prediction” within Evidence Modeler.

As second evidence, coding sequences (CDS) from 22 available lepidopteran species were downloaded from Lepbase. CDS were translated to corresponding peptide sequence using Prank (v.170427) [32]. CDS and peptide sequences were co-aligned to the assembly of *H. vespertilio* using GenomeThreader [33] with the parameters “-gcmincoverage 70 -paralogs -species drosophila**”**. Species used and number of significant alignments are detailed in Supplementary Table 2. Alignments were passed to Evidence Modeler as “Protein” alignments and assigned a weight of 4.

As a third evidence, short read RNA sequencing data for the closely related *H. euphorbiae* was mapped to the *H. vespertilio* assembly using hisat2 (v 2.0.0), with parameters “--dta --no-unal --mp 4,1 --score-min L,0,-0.125”. Transcripts were assembled with Stringtie [15], with default parameters. Fasta sequences for each transcript were extracted using bedtools. Open reading frames were predicted for each transcript using GenemarkS-T (v5.1) [16] with parameters “--strand direct”. GenemarkS-T transcripts were given a weight of 7 and classed as “Other prediction” within Evidence Modeler. TAMA

(https://github.com/GenomeRIK/tama.git) was also used to identify ORFs within assembled transcripts. Briefly, ORFs are predicted from all forward frames of a transcript. Predicted peptide sequences are then queried using Blastp [34] against a blast database of the downloaded Lepbase proteins and further classified as full length or partial hits. The highest scoring ORF for each transcript is mapped back to the transcript, and putative nonsense mediated decay (NMD) targets were determined and excluded. Full length and non-NMD target transcripts were provided to Evidence Modeler as class “Other prediction” with a weight of 7. The remaining transcripts were provided to Evidence Modeler as “Transcript” alignments with a weight of 4.

As a fourth evidence, four *ab initio* gene prediction tools were used to predict genes in the *H. vespertilio* genome. As training data, we used a set of non-overlapping, full length and intact genes that were projected from *B. mori* and *M. sexta* and resulted in an identical gene model in *H. vespertilio* (2504 genes). This set was randomly divided into 80% training data and 20% test data. SNAP [18] and GlimmerHMM [19] were trained as per the available manuals, and genes were predicted. To run Augustus [20], we first mapped the RNAseq data again to the genome with using hisat2 and strict parameters (--no-mixed --no-discordant --dta --no-unal --n-ceil L,0,0.05) to generated hints. Intron positions as predicted by spliced alignments were extracted using the bam2hints module from Augustus (v3.3.1). The heliconius_melpomene1 model provided with Augustus was optimized for *H. vespetilio* using optimize_augustus.pl (--cpus=12 --kfold=12) and the training gene set, and Augustus was further trained using these parameters with the etraining tool. Genes were then predicted with Augustus, providing the intron positions as extrinsic hints. Finally, intron hints were provided to Genemark-ES [21] for self-training and gene prediction. Gene predictions were evaluated against the test gene set using Parseval [35]. SNAP and GlimmerHMM were subsequently given a weight of 1, while Augustus and Genemark-ES predictions were given a weight of 2. All were provided as type “*ab initio* prediction”.

Evidence Modeler [13] was then run using the above evidences and described weights. Full length, functional TOGA projections from *M. sexta* and *B. mori* with

no CDS overlap to any consensus model were included into the consensus set. Consensus gene models with greater than 50% CDS overlap within a single repeat region, as annotated by RepeatMasker, were removed. To assess completeness of the gene annotation, we applied BUSCO v3 [22] in protein mode to our final *H. vespertilio* protein set and the annotated *B. mori* and *M. sexta* proteins, using the Endopterygota dataset.

AVAILABILITY OF SUPPORTING DATA

All raw sequencing data and genome assemblies are available at the National Center for Biotechnology Information under the BioProject ID PRJNA574010 (uploaded, Acc. No. is pending). The genome, our gene annotations including the gene evidences, and genome alignments to *B. mori* and *M. sexta* are available for download at https://bds.mpi-cbg.de/hillerlab/HylesGenomeData/ and for genome browser visualization and exploration at https://genome-public.pks.mpg.de/cgi-bin/hgTracks?db=HLhylVes1.

ADDITIONAL FILES

Supplementary Tables 1-2, Supplementary Figures 1-2, Supplementary Data files 1-3

COMPETING INTERESTS

The authors declare no competing interests.

FUNDING

This work was funded by the Max Planck Gesellschaft (Michael Hiller, Gene Myers) and DFG HU 1561/5-1 & RE 603/25-1. It benefitted from the sharing of expertise within the DFG priority program SPP 1991 Taxon-Omics.

ACKNOWLEDGEMENTS

We thank Alberto Zilli (London) for the collection of the two *Hyles vespertilio* males and the Long Read platform of the DRESDEN-concept Genome Center, DFG NGS Competence Center, c/o Center for Molecular and Cellular Bioengineering (CMCB), Technische Universität Dresden, Dresden, Germany for DNA isolation and Pacbio long read sequencing.

REFERENCES

1. van Nieukerken EJ, Kaila L, Kitching IJ, Kristensen NP, Lees DC, Minet J, et al. Order Lepidoptera Linnaeus, 1758. Zootaxa. 2011;3148:212-21.
2. Kitching IJ, Rougerie R, Zwick A, Hamilton CA, St Laurent RA, Naumann S, et al. A global checklist of the Bombycoidea (Insecta: Lepidoptera). Biodiversity Data Journal. 2018; 6.
3. Lautenschläger T, Neinhuis C, Monizi M, Mandombe JL, Förster A, Henle T, et al. Edible insects of Northern Angola. African Invertebrates. 2017;58:55.
4. Kawahara AY and Barber JR. Tempo and mode of antibat ultrasound production and sonar jamming in the diverse hawkmoth radiation. Proceedings of the National Academy of Sciences. 2015;112 20:6407-12.
5. del Campo C ML and Renwick JAA. Dependence on host constituents controlling food acceptance by *Manduca sexta* larvae. Entomologia Experimentalis et Applicata. 1999;93 2:209-15.
6. International Silkworm Genome C. The genome of a lepidopteran model insect, the silkworm *Bombyx mori*. Insect Biochemistry and Molecular Biology. 2008;38 12:1036-45.
7. Kanost MR, Arrese EL, Cao X, Chen Y-R, Chellapilla S, Goldsmith MR, et al. Multifaceted biological insights from a draft genome sequence of the tobacco hornworm moth, *Manduca sexta*. Insect Biochemistry and Molecular Biology. 2016;76:118-47.
8. Hundsdoerfer AK, Päckert M, Kehlmaier C, Strutzenberger P and Kitching IJ. Museum archives revisited: Central Asiatic hawkmoths reveal exceptionally high late Pliocene species diversification (Lepidoptera, Sphingidae). Zoologica Scripta. 2017;46 5:552-70.
9. Hundsdoerfer AK, Rubinoff D, Attié M, Kitching IJ and Wink M. A revised molecular phylogeny of the globally distributed hawkmoth genus *Hyles* (Lepidoptera: Sphingidae), based on mitochondrial and nuclear DNA sequences. Molecular Phylogenetics and Evolution. 2009;52:852–65.
10. *Hyles*. Sphingidae taxonomic inventory. Scratchpads. Biodiversity online, 2019. http://sphingidae.myspecies.info/taxonomy/term/1276. Accessed 5.7.2019.

11. Nowoshilow S, Schloissnig S, Fei JF, Dahl A, Pang AWC, Pippel M, et al. The axolotl genome and the evolution of key tissue formation regulators. Nature. 2018;554 7690:50-5.
12. Grohme MA, Schloissnig S, Rozanski A, Pippel M, Young GR, Winkler S, et al. The genome of *Schmidtea mediterranea* and the evolution of core cellular mechanisms. Nature. 2018;554 7690:56-61.
13. Haas BJ, Salzberg SL, Zhu W, Pertea M, Allen JE, Orvis J, et al. Automated eukaryotic gene structure annotation using EVidenceModeler and the Program to Assemble Spliced Alignments. Genome Biology. 2008;9 1:R7.
14. Sharma V, Schwede P and Hiller M. CESAR 2.0 substantially improves speed and accuracy of comparative gene annotation. Bioinformatics. 2017;33 24:3985-7.
15. Pertea M, Pertea GM, Antonescu CM, Chang TC, Mendell JT and Salzberg SL. StringTie enables improved reconstruction of a transcriptome from RNA-seq reads. Nature Biotechnology. 2015;33 3:290-5.
16. Tang S, Lomsadze A and Borodovsky M. Identification of protein coding regions in RNA transcripts. Nucleic Acids Research. 2015;43 12:e78.
17. Jung S, Pausch H, Langenmayer MC, Schwarzenbacher H, Majzoub-Altweck M, Gollnick NS, et al. A nonsense mutation in PLD4 is associated with a zinc deficiency-like syndrome in Fleckvieh cattle. BMC Genomics. 2014;15:623.
18. Korf I. Gene finding in novel genomes. BMC Bioinformatics. 2004;5:59.
19. Majoros WH, Pertea M and Salzberg SL. TigrScan and GlimmerHMM: two open source ab initio eukaryotic gene-finders. Bioinformatics. 2004;20 16:2878-9.
20. Stanke M, Schoffmann O, Morgenstern B and Waack S. Gene prediction in eukaryotes with a generalized hidden Markov model that uses hints from external sources. BMC Bioinformatics. 2006;7:62.
21. Ter-Hovhannisyan V, Lomsadze A, Chernoff YO and Borodovsky M. Gene prediction in novel fungal genomes using an ab initio algorithm with unsupervised training. Genome Research. 2008;18 12:1979-90.
22. Waterhouse RM, Seppey M, Simao FA, Manni M, Ioannidis P, Klioutchnikov G, et al. BUSCO applications from quality assessments to gene prediction and phylogenomics. Molecular Biology and Evolution. 2017.
23. Mende MB and Hundsdoerfer AK. Mitochondrial lineage sorting in action - historical biogeography of the *Hyles euphorbiae* complex (Sphingidae, Lepidoptera) in Italy. BMC Evolutionary Biology. 2013;13 1:83.
24. Hundsdoerfer AK, Mende MB, Harbich H, Pittaway AR and Kitching IJ. Larval pattern morphotypes in the Western Palaearctic *Hyles euphorbiae* complex (Lepidoptera: Sphingidae: Macroglossinae). Insect Systematics and Phylogeny. 2011;42:41-86.
25. Tischler G. Haplotype and repeat separation in long reads. bioRxiv. 2017:145474.
26. Tischler G and Myers EW. Non hybrid long read consensus using local de Bruijn graph assembly. bioRxiv. 2017:106252.
27. Challis RJ, Kumar S, Dasmahapatra KK, Jiggins CD and Blaxter M. Lepbase: the Lepidopteran genome database. BioRxiv. 2016:056994.
28. Harris RS. *Improved pairwise alignmnet of genomic DNA*. The Pennsylvania State University, Pennsylvania 2007.
29. Kent WJ, Baertsch R, Hinrichs A, Miller W and Haussler D. Evolution's cauldron: duplication, deletion, and rearrangement in the mouse and human

genomes. Proceedings of the National Academy of Sciences of the United States of America. 2003;100 20:11484-9.
30. Suarez HG, Langer BE, Ladde P and Hiller M. chainCleaner improves genome alignment specificity and sensitivity. Bioinformatics. 2017;33 11:1596-603.
31. Osipova E, Hecker N and Hiller M. RepeatFiller newly identifies megabases of aligning repetitive sequences and improves annotations of conserved non-exonic elements. bioRxiv. 2019:696922.
32. Loytynoja A. Phylogeny-aware alignment with PRANK. Methods in Molecular Biology. 2014;1079:155-70.
33. Gremme G, Brendel V, Sparks ME and Kurtz S. Engineering a software tool for gene structure prediction in higher organisms. Information and Software Technology. 2005;47 15:965-78.
34. Camacho C, Coulouris G, Avagyan V, Ma N, Papadopoulos J, Bealer K, et al. BLAST+: architecture and applications. BMC Bioinformatics. 2009;10 1:421.
35. Standage DS and Brendel VP. ParsEval: parallel comparison and analysis of gene structure annotations. BMC Bioinformatics. 2012;13 1:187.

FIGURES

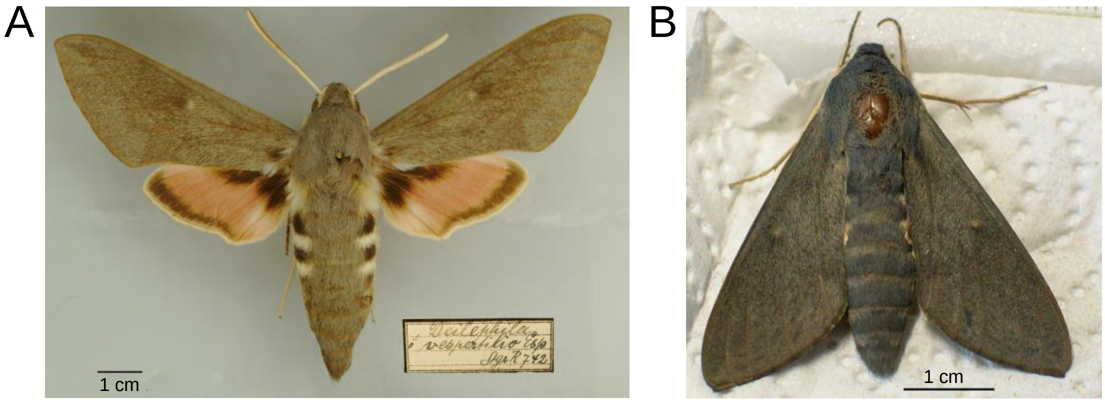

Figure 1: *Hyles vespertilio*.

(A) The lectotype specimen, originally described as *Sphinx vespertilio* by Esper in 1779, was collected in the "area of Verona" (Italy) and deposited in the "Landesmuseum für Kunst und Natur" (Wiesbaden, Germany).

(B) The specimen collected in Vallonina (Italy) in 2018. Nearly all tissue was used to sequence the genome. The wings are deposited in the Museum of Zoology (Senckenberg Natural History Collections Dresden, Germany). Scale bars: 1 cm.

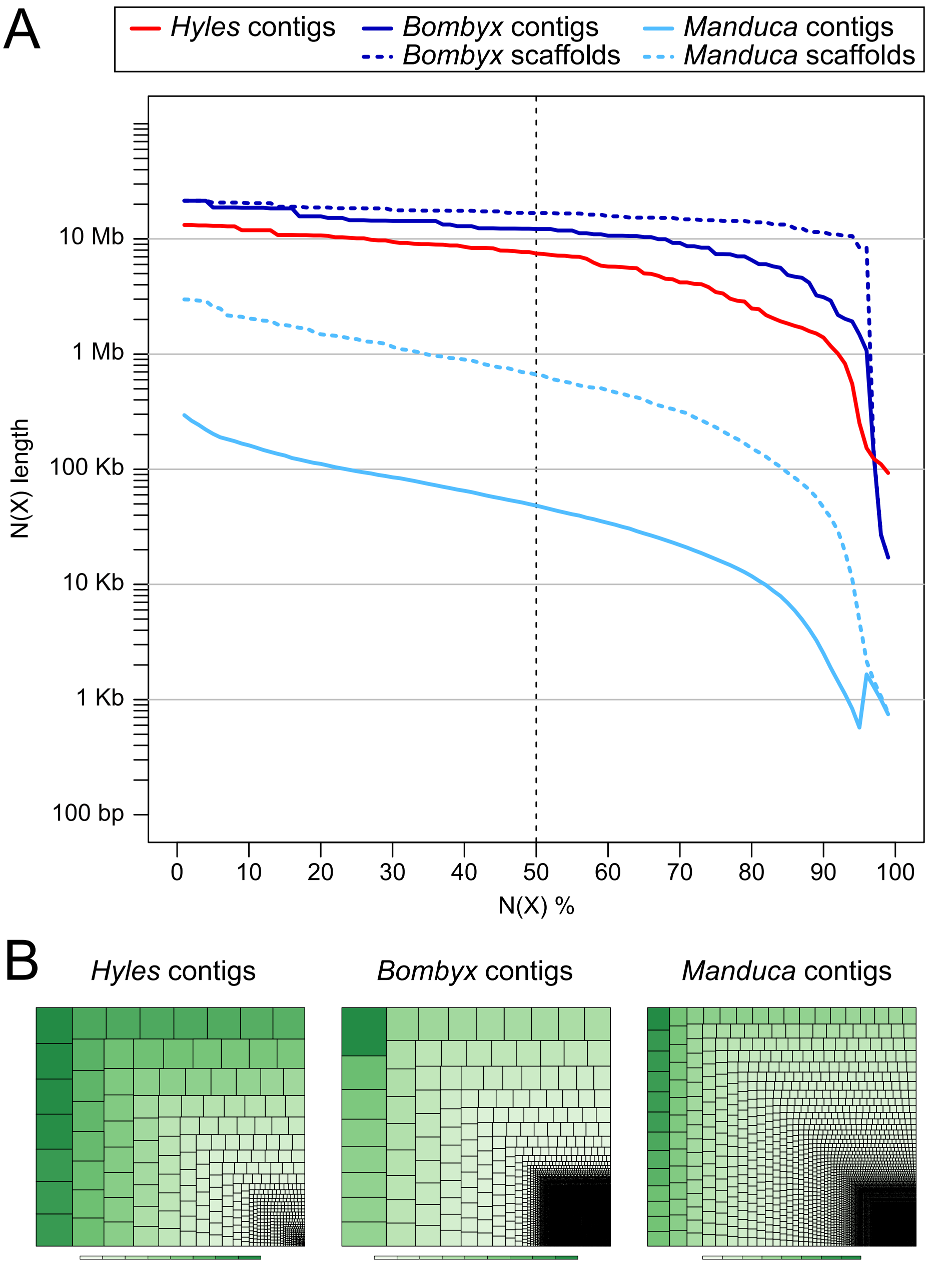

Figure 2: Assembly contiguity.

(A) The N(x)% graph shows the contig or scaffold sizes on the y-axis, where x% of the genome assembly consists of contigs or scaffolds of at least that size. Contigs are shown as solid curves, scaffolds (only for *B. mori* and *M. sexta*) as dashed curves. The N50 value is marked as a vertical dashed line.

(B) Treemap comparison between the MARVEL *H. vespertilio* assembly and the assemblies of *B. mori* [6] and *M. sexta* [7]. Squares encode the relative contributions of individual scaffolds or contigs to assembly size.

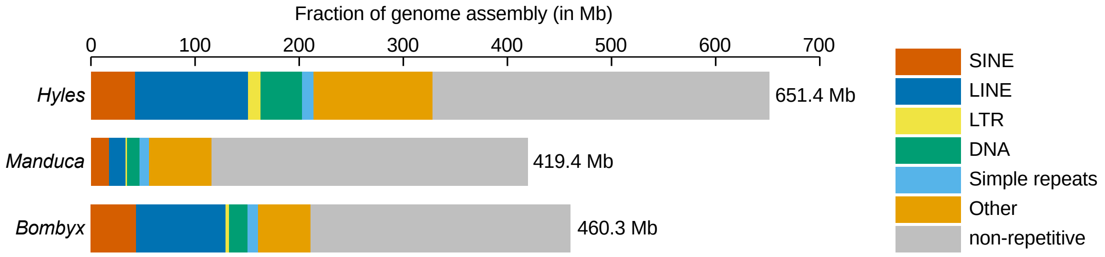

Figure 3: Comparison of genomic repeat content.

Stacked bar charts represent the portion of the genome assembly covered by major classes of repetitive elements. Grey indicates non-repetitive genomic regions. Simple repeats comprise tandem repeats, low complexity regions and satellite repeats. The total assembly size is provided right of the bars.

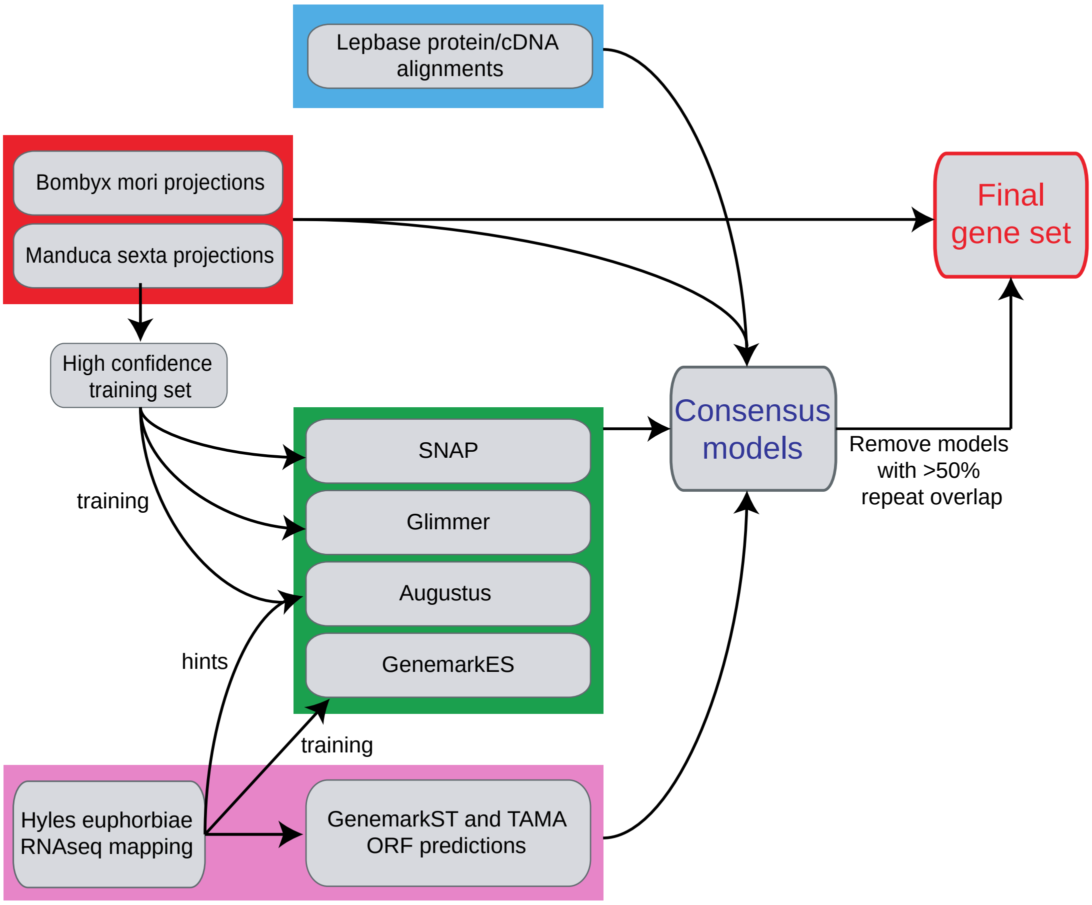

Figure 4: Gene annotation strategy.

Protein and coding sequences from 22 Lepidopteran species were downloaded from Lepbase and aligned to the *H. vespertilio* genome (blue box). Genome alignments were used to project coding gene annotation from *B. mori* and *M. sexta* to *H. verspertilio* with CESAR 2.0 (red box). RNA-seq reads from a related *Hyles* species were aligned to the genome and used to assemble transcripts, followed by predicting open reading frames with two methods (purple box). High quality gene projections and/or RNA-seq evidence were used to train four *ab initio* gene prediction tools (green box). All evidences were combined into a consensus set using EVidenceModeler and filtered for models that overlap genomic repeats. Finally, these filtered gene models were combined with full length gene projections that did not overlap consensus genes model to produce the final gene set.

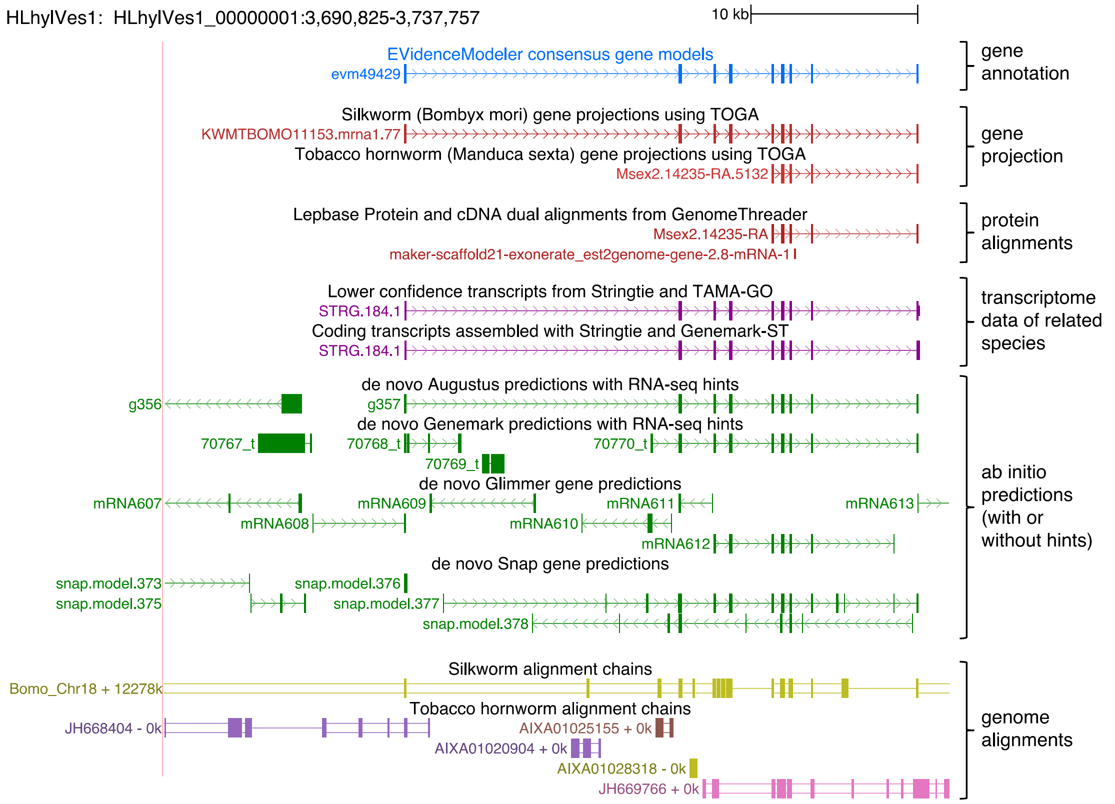

Figure 5: Genome browser visualization of annotations generated for the *H. vespertilio* genome.

A UCSC genome browser instance visualizes the final gene annotation (blue), together with the underlying gene evidences, and pairwise genome alignment chains to *B. mori* and *M. sexta*.

Supplementary Figures

Click here to access/download
Supplementary Material
Supplementary_Figures.pdf

Click here to access/download
Supplementary Material
Supplementary_Tables.xlsx

Assembly instructions

Click here to access/download
Supplementary Material
Supplementary_DataFile1.txt

Assembly instructions

Click here to access/download
Supplementary Material
iHylVes.assembly.config.sh

Assembly instructions

Click here to access/download
Supplementary Material
iHylVes.coverage.config.sh
