# Supplementary material for: A highly contiguous genome assembly of the bat hawkmoth Hyles vespertilio (Lepidoptera: Sphingidae)
Source: Gigascience. 2020 Jan 23;9(1):giaa001. doi: 10.1093/gigascience/giaa001 (PMC6977585; doi:10.1093/gigascience/giaa001)
Supplement: giaa001_Supplemental_Files [file giaa001_supplemental_files.zip › Supplement.pdf]

Supplementary Information for

A highly contiguous genome assembly of the bat hawkmoth *Hyles vespertilio* (Lepidoptera: Sphingidae)

Martin Pippel [1, 2, a] , David Jebb [1, 2, 3, a], Franziska Patzold [4], Sylke Winkler [1], Gene Myers [1], Heiko Vogel [5], Michael Hiller [1, 2, 3, #] Anna K. Hundsdoerfer [4, #]

[1] Max Planck Institute of Molecular Cell Biology and Genetics, Pfotenhauerstraße 108, 01307 Dresden, Germany
[2] Center for Systems Biology Dresden, Pfotenhauerstr. 108, 01307, Dresden, Germany
[3] Max Planck Institute for the Physics of Complex Systems, Nöthnitzer Str. 38, 01187, Dresden, Germany
[4] Senckenberg Natural History Collections Dresden, Königsbrücker Landstr. 159, 01109 Dresden, Germany
[5] Department of Entomology, Max Planck Institute for Chemical Ecology, Hans-Knoell-Strasse 8, 07745, Jena, Germany

[a] Joint first authorship

[#] Corresponding authors:
hiller@mpi-cbg.de & Anna.Hundsdoerfer@senckenberg.de

The Supplementary Material contains
- Figures 1-3
- Text 1-2

Supplementary Tables 1-5 and Supplementary Data files 1-3 are given as separate files.

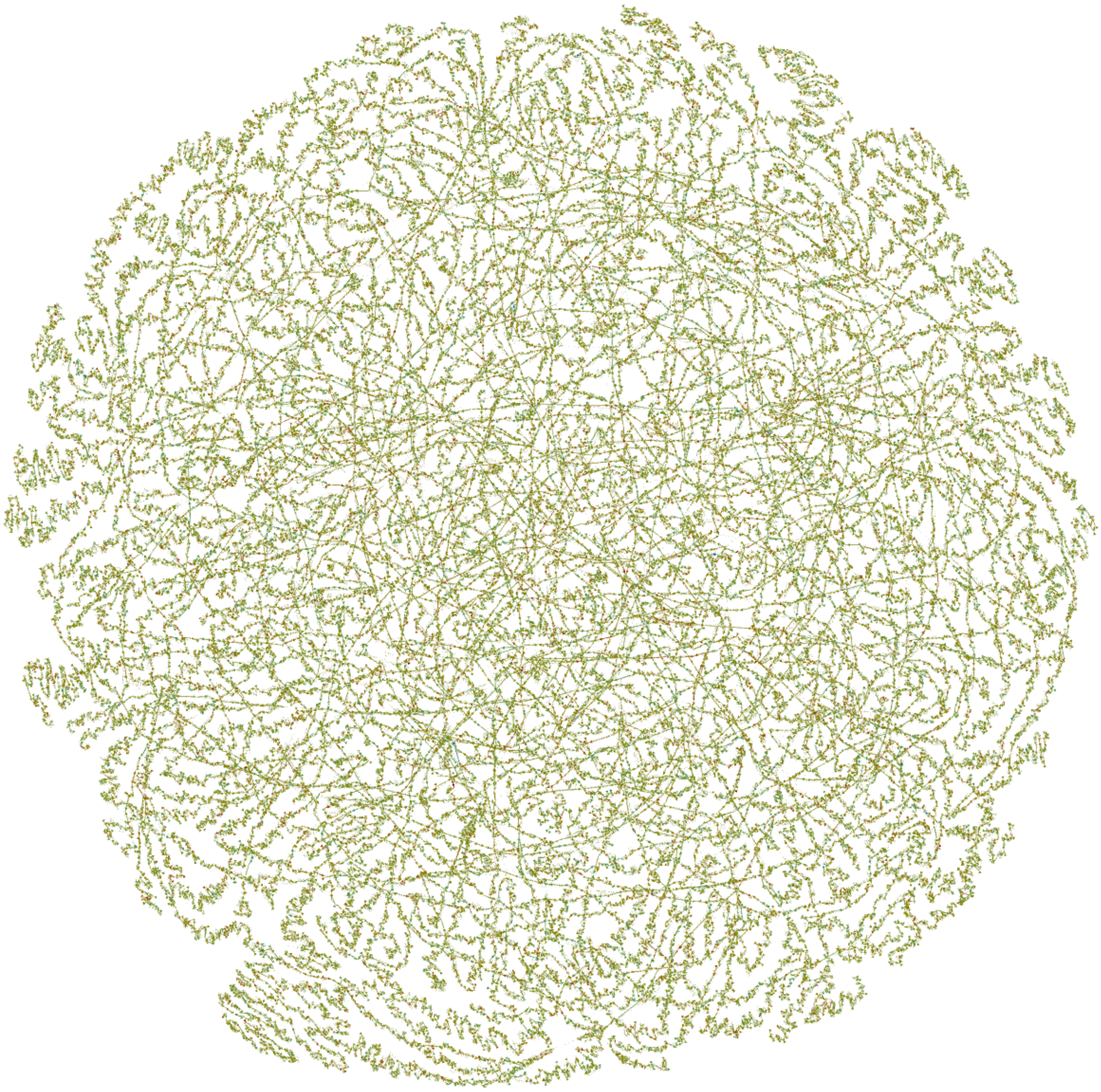

Supplementary Figure 1: Final overlap graph without the additional chimeric read removal step.

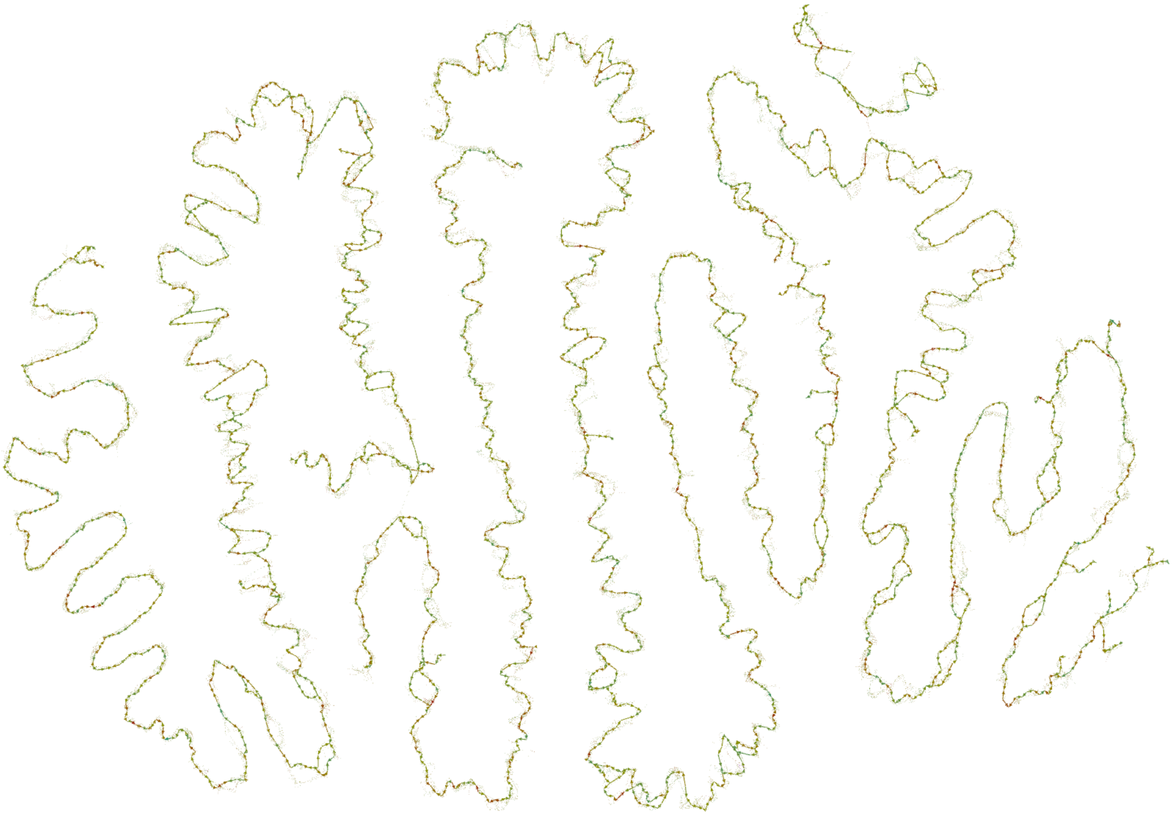

Supplementary Figure 2: Final overlap graph after applying the additional chimeric read removal step.

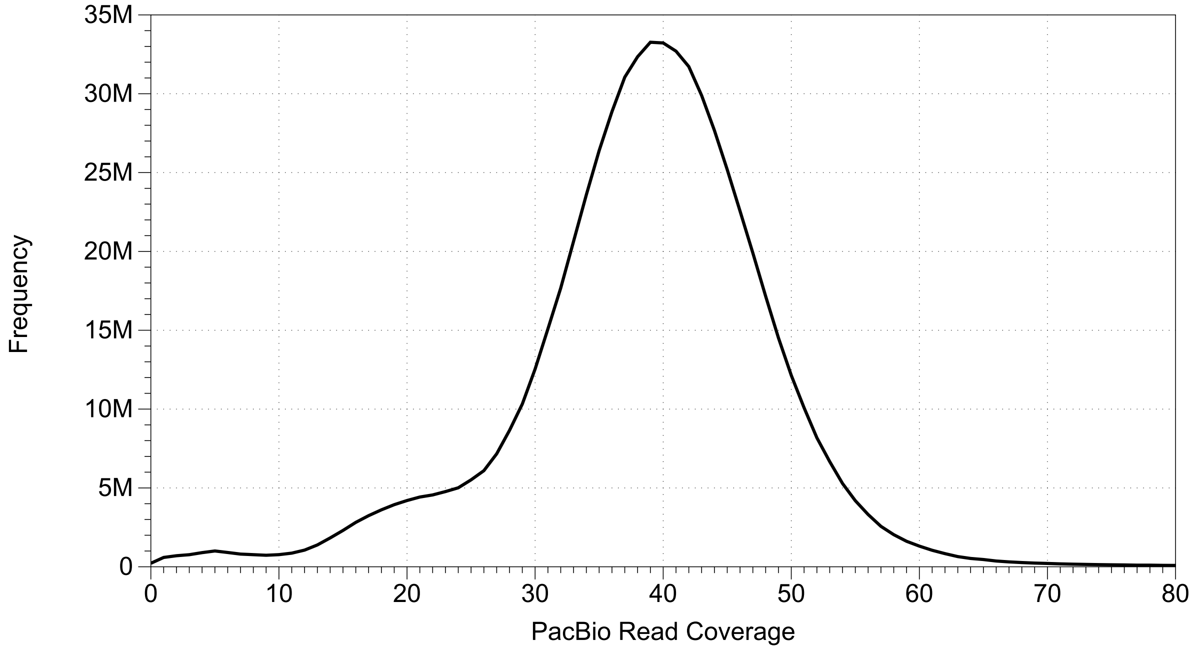

Supplementary Figure 3: Per base read coverage histogram.
The peak around 40X coincides with the raw sequencing coverage of 45X (a small drop due to mapping issues and noisy reads is expected). The small hump around 20X indicates that some contigs or contig parts are alternative haplotypes. These putative contigs are listed in Supplementary Table 4.

Supplementary Text 1

The Dazzler suite of tools is described in the dazzlerblog (https://dazzlerblog.wordpress.com/). For the potential case that this page becomes unavailable in future, we copied the relevant parts in this Supplementary Text 1.

DALIGNER: Fast and sensitive detection of all pairwise local alignments

Posted on July 10, 2014 by Gene Myers

The first phase of the Dazzler assembler compares every read in the trimmed Dazzler database (DB) against every other read in the trimmed DB, seeking significant *local alignments* between them. In an old-fashioned OLC assembler, one would look, more restrictively, for *overlaps* between reads. But one can use local alignments to detect artifacts such as chimeric reads and unclipped adapter sequence by looking for consistent alignment terminations within a read, and one can also detect and annotate repetitive elements of the genome covered within a read by looking for "pile-ups" of alignments. Removing the artifacts and knowing the repetitive regions covered by a read is a huge advantage prior to assembling the reads. Indeed, the dazzler assembler employes several downstream phases specifically to remove artifacts, correct reads, and annotate repetitive elements prior to building a string graph which is the modern analog of the layout phase for an OLC architecture.

You can get the source code for the DALIGNER module on Github here. Grabbing the code and uttering "make" should produce 9 programs: **daligner**, **LAsort**, **LAmerge**, **LAshow**, **LAcat**, **LAsplit**, **LAcheck**, **HPCdaligner**, and **HPCmapper**.

Ideally one would like to call the alignment finder, **daligner**, with a DB, e.g. "`daligner MyDB`", and after some computing it would output a file encoding all the found local alignments. Unfortunately for big projects the amount of memory and CPU time required make this impossible. For example, on the Pacbio 54X human data set, **daligner** took 15,600 CPU hours on our modest 480 core HPC cluster, and as a monolithic run would have required at least 12Tb of memory! I could start apologizing, but consider that the only other program that can compare reads at a 12-15% error rate, BLASR, took 404,000 CPU hours, and only delivered overlaps. Indeed, a primary reason for releasing **daligner** now, and not waiting to release a complete end-to-end assembler is precisely to provide the bioinformatics community with a feasible way to compute alignments for Pacbio data at this scale. While we've embedded **daligner** within our database framework, it is easy to grab **daligner**'s output in an ASCII format and then use that in an HGAP pipeline (which is the only current end to end assembler publicly available).

So given the scale of the required computation we have to "bite the bullet" and take a job parallel, HPC approach to effect an all-against-all comparison of the trimmed DB. So as a running example, let's suppose you've set up a DB that is partitioned and optionally has a "dust" track (see this blog post) with a command sequence like:

```
fasta2DB DB MyData1.fasta MyData2.fasta ...
DBdust DB
DBsplit -x1000 DB
```

Suppose for simplicity that *DB* splits into 3 blocks. Then calling **HPCdaligner** on the DB will produce a UNIX shell script that contains all the commands needed to effect the all against all comparison. For example, "`HPCdaligner DB`" produces the script:

```
# Daligner jobs (3)
daligner DB.1 DB.1
daligner DB.2 DB.1 DB.2
daligner DB.3 DB.1 DB.2 DB.3
# Initial sort jobs (9)
LAsort DB.1.DB.1.*.las && LAmerge L1.1.1 DB.1.DB.1.*.S.las && rm DB.1.DB.1.*.las
LAsort DB.1.DB.2.*.las && LAmerge L1.1.2 DB.1.DB.2.*.S.las && rm DB.1.DB.2.*.las
LAsort DB.1.DB.3.*.las && LAmerge L1.1.3 DB.1.DB.3.*.S.las && rm DB.1.DB.3.*.las
LAsort DB.2.DB.1.*.las && LAmerge L1.2.1 DB.2.DB.1.*.S.las && rm DB.2.DB.1.*.las
…
# Level 1 merge jobs (3)
LAmerge DB.1 L1.1.*.las && rm L1.1.*.las
LAmerge DB.2 L1.2.*.las && rm L1.2.*.las
LAmerge DB.3 L1.3.*.las && rm L1.3.*.las
```

Each non-comment line of the script should be submitted to your cluster as a job, and all the jobs following one of the 3 comment lines can be run in parallel, but these 3 batches of job groups need to be run in sequence. That is, the 3 "Daligner" jobs can be run in parallel as a group, and once they have all completed, one can then run the 9 "Initial sort" jobs in parallel as a group, and finally the "Level 1 merge" jobs as a group. And of course, for a small script like this one, you could just invoke the shell on the script and let the jobs run sequentially on your desktop, e.g. "`HPCdaligner DB | csh`".

When all the jobs of our sample script have completed there will be 3 sorted **alignment files** *DB.1.las*, *DB.2.las*, *DB.3.las* that encode the alignments found for the reads in each of the 3 trimmed DB blocks, respectively. To describe the contents of these 3 files precisely, one must first know that for each alignment, a *.las*-file records:

$a[ab,ae] \times b^{comp}[bb,be]$

where *a* and *b* are the indices (in the trimmed DB) of the reads that overlap, *comp* indicates whether the *b*-read is from the same or opposite strand, and *[ab,ae]* and *[bb,be]* are the intervals of *a* and b^{comp}, respectively, that align. It is further the case that an alignment between reads *a* and *b* results in two alignment records, one for *a* versus *b* and another for *b* versus *a*. In addition, a series of **trace points** is retained with each alignment record to facilitate the rapid delivery of an optimal alignment between the relevant intervals of the reads. We can now explain that the output file *DB.n.las* contains all the overlap records for which the first read *a* is in the trimmed block *DB.n* and these records further occur in sorted order of *a*, then *b*, then *comp*, and finally *ab*. What this means is that all the alignments involving a given *a*-read occur in a contiguous interval of the output files permitting easy and job parallel

computation in the downstream phases for chimer detection, read correction, repeat analysis, etc..

The **HPCdaligner** UNIX shell script employes the three commands **daligner**, **LAsort**, and **LAmerge** to produce the sorted and partitioned *.las* files for each of the *N* blocks of a hypothetical database *D* as follows:

- "Daligner" step: Every block is compared against every other block by calling **daligner** on every pair *D.x* and *D.y* such that *y≤x*. A call such as "`daligner D.x D.y`$_1$ `.. D.y`$_k$" performs *k* comparisons of block *D.x* versus each block *D.y*$_i$ and does so with a compiled number of threads *T* (default 4). Each of these pairwise block comparisons produces *2T* unsorted *.las* files *D.x.D.y.[C|N]t.las* and if *x > y*, an additional *2T* unsorted *.las* files *D.y.D.x.[C|N]t.las*. The file *D.x.D.y.Ot.las* contains every alignment produced by thread *t* where the *a*-read is in *D.x and* the *b*-read is in *D.y* and is in orientation *O*. In total *(N+1)N/2* blocks are compared and $2N^2T$ *.las* files result.
- "Initial sort" step: The *2T .las* files for each block pair *x,y*, are sorted and merged into a single sorted *.las* file. The *2T* files are first sorted with **LAsort**, where for each unsorted file *U.las* the command produces the sorted overlap file *U.S.las*. These sorted *.S.las* files are then merged into a single sorted file, *L1.x.y.las*, with **LAmerge** and afterwords removed. At the end of this step there are exactly N^2 sorted *.las* files, one for each block pair.
- "Level k merge" steps: In a sequence of *O(log N)* levels the *N* files *L1.x.*.las* for a given block *x* are merged by **LAmerge** into a single *.las* file, *D.x.las*. At level 1, the *N* sorted files for a block are merged in groups of up to 25 resulting in ⌊*N/25*⌋ files, that in level 2 are merged in groups of up to 25, resulting in ⌊*N/625*⌋ files, and so on until only one file *D.x.las* remains. Even big projects involve less than 625 blocks, so typically only one or at most two merge levels are required.

For extraordinarily large projects the computation can be performed in stages as new data is added to the database, thus amortizing the cost of finding alignments over the interval of data production. For example, suppose you have a really big project going and that thus far the DB has 78 blocks in its current partition. Executing the script produced by "`HPCdaligner DB 1-77`" will compare blocks 1 to 77 versus each other producing files *DB.1.las* through *DB.77.las*. Note carefully that the last block, 78, was not included, because it is a partial block. Later on suppose you've added more data and now have 144 blocks. Then executing the script produced by "`HPCdaligner DB 78-143`" will compare blocks 78 to 143 against each other and against the earlier blocks 1 through 77, updating *DB.1.las* through *DB.77.las* and creating *DB.78.las* through *DB.144.las*. And so on. The only restrictions are that (a) one must add the blocks in sequential order once only, and (b) one should never add the last, partial block until the very last increment. By submitting each new complete block as it appears, I figure one could keep up with data production for say a 100X shotgun of *D. melanogaster* on a decent laptop.

The command **LAshow** produces an ASCII display of all or a selected subset of the alignments encoded in a given *.las* file, sorted or unsorted. One can view just the read indices and their aligned intervals, one per line, or a line rendering of the relationship between the two reads, or a complete BLAST-style alignment of each local alignment where one has several options for customizing the display. In addition to permitting one to browse local alignments, the

ASCII output can also be fed into a python or pearl script that converts the information to a from that can be input to an external program of your choosing, such as the read correction step of the HGAP assembler.

The **LAcat** and **LAsplit** commands allow one to effectively change the partition of the *.las* files for a project should it ever be necessary. Suppose the *.las* files *D.1.las*, *D.2.las*, …, *D.N.las* have been computed for a database *D.db* that has been partitioned into *N* blocks. "`LAcat D.# >E.las`" will find all the *.las* files of the form *D.#.las* and stream the conceptual concatenation of all the *.las* files in numeric order to the standard output, in this case to the file *E.las*. In the other direction, "`LAsplit F.# N <E.las`" will split *E.las* as evenly as possible into *N* block files, *F.1.las*, *F.2.las*, …, *F.N.las* subject to the restriction that the alignments involving a given *a*-read are all in the same file. Note that *F.x.las* does not necessarily equal the original *D.x.las*, as the *a*-reads in *F.x.las* may not be the same as the reads in a given block, *D.x.db,* because the splitting criteria for **LAsplit** and **DBsplit** are not the same. To get exactly the same files back, one should instead utter "`LAsplit F.#.las D.db <E.las`" or more tersely "`LAsplit F.# D <E.las`" that splits the piped input according to the partitioning of database *D*! Finally, observe that if you want to repartition the DB *D* and also all the *D.#.las* files, first call **DBsplit** on *D*, and afterwords call "`LAcat D.# | LAsplit D.# D`" to redistribute the alignment records into *.las* files according to the new partitioning.

Lastly, the command **LAcheck** reads through the *.las* files given to it and checks them for structural integrity, i.e. do they reasonably encode a properly formatted *.las* file? We find this command useful in ensuring that every stage of a large **HPCdaligner** script has been properly executed. In processes involving thousands of jobs, it is not unusual for one or two to go "haywire". So while we didn't build it into the **HPCdaligner** scripts, we strongly recommend that you run **LAcheck** on *.las* files throughout the process to make sure all is well. Doing so is standard operating procedure for our assembly group.

A precise, detailed, and up-to-date command reference can be found here.

Intrinsic Quality Values

Posted on **November 6, 2015** by **Gene Myers**

One of the most vexing problems with Pacbio data is that it is very difficult to understand the quality of a given small segment or region of a read. The Q-scores that come out of the instrument are not particularly informative in this regard, their value seems to be mostly in indicating if a given base is better or worse than the bases neighboring it (and this *is* of importance to multi-aligners and consensus callers like Quiver). I tried every way I could think of to aggregate these Q-scores over a say 100bp interval so as to arrive at an informative statistic about the overall quality of that 100bp stretch and failed. But having this information is very important because over the course of a long 10-40Kbp read, the quality of the base calls varies significantly, where there are even long internal stretches that are essentially junk. I illustrate a typical Pacbio read error profile to help you further understand why I think it is extremely important to understand the "regional" quality of a read.

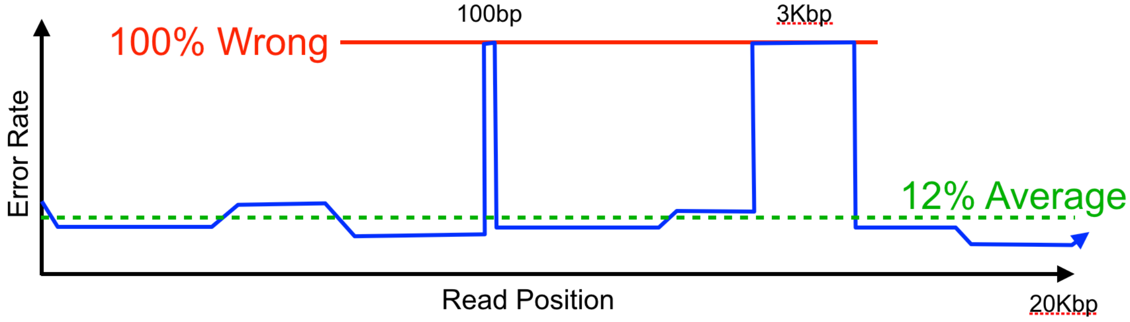

My solution to determining read quality begins with the idea of a **pile**, which is the set of all alignments that involve a given read, say a, as the A-read in an alignment pair (a,b). Recall that the **HPCdaligner** script arranges to sort all the alignments between reads found by **daligner** so that they are in order of A-read, then B-read, then B-orientation, etc. In particular, this means that for a given read, say 103, one encounters all the alignments with 103 as the A-read in consecutive order in a *.las* file. That is the 103-pile and in fact every pile can be collected and processed consecutively as one scans a *.las* file! All post-overlap and pre-assembly analyses in the Dazzler suite are based on looking at read piles.

The figure below shows a pile in a graphical form I call a **pile-ogram**. The long yellow bar at the top represents the A-read and every white bar below it represents the A-interval of an alignment with another read. The brown tips indicate that the B-read continues but does not align with the A-read (note that some white bars have brown tips, others do not).

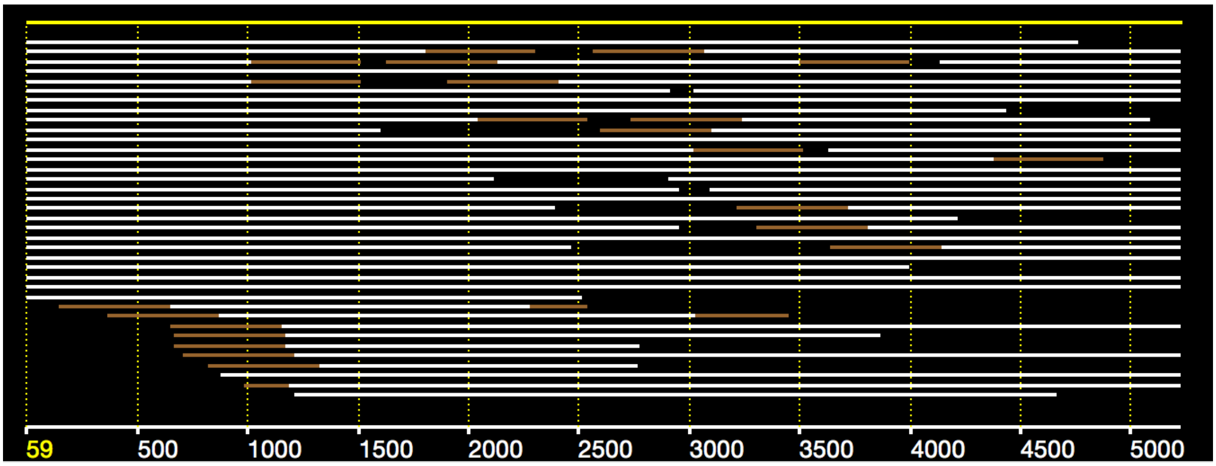

Recall from the previous post on **trace points**, that for each alignment we record the number of differences in the alignment for each section between tick marks spaced Δ base pairs apart in A. **Daligner** uses a default of Δ = 100, so the number of differences is also the percentage mismatch between the two reads over each 100bp stretch of the A-read (save possibly at the very beginning and very end of an alignment that spans only a part of a 100bp stretch). In the pile-ogram below, we have color-coded each 100bp interval of each alignment according to the number of difference in the segment, where the heat map scale is given just above the pile-ogram.

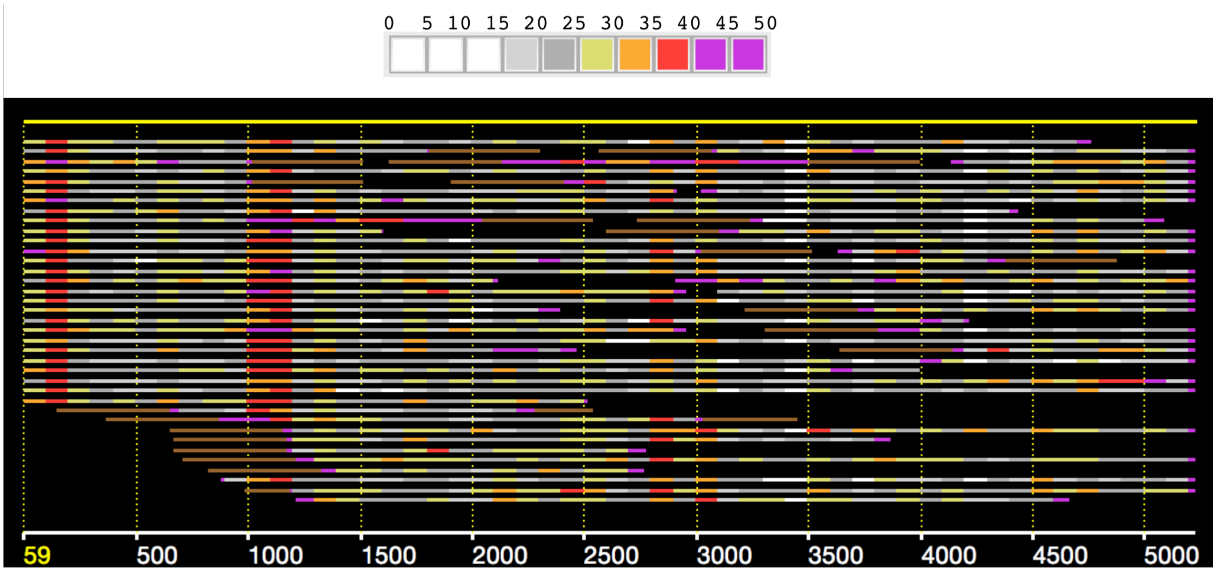

It should immediately be obvious that in the 100bp "columns" that are all orange, red, and purple, every B-read disagrees with the corresponding segment of the A-read and the clear inference is that the quality of the A-read is very low in that segment. Going a little further, if a column has say 40 alignments covering it and the average error rate of these is 12%, then it is very unlikely that 50% or more of the 40 B-reads are worse than average in their portions of the alignment spanning the column and indeed we expect the average quality of this 50% to be quite stable, perhaps from 8-12%. The variable part that mostly determines the color code must then be the quality of the A segment so a good proxy statistic for the quality of each 100bp segment of the A-read is the average match fidelity of the best say 25-50% of the alignments covering the segment. In the figure below we now color code the segments of the A-read according to their **intrinsic quality value**. We use the adjective "intrinsic" to emphasize that this statistic was derived solely from the read data set itself.

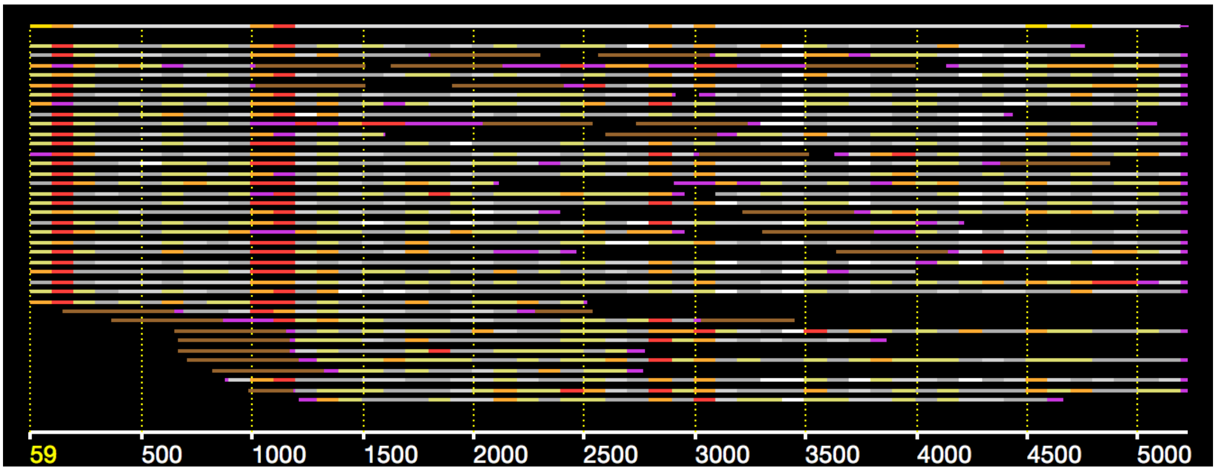

In the case that there is a large drop out in the quality of an A-read, this tends to "break" local alignments as the alignment software cannot find a good correspondence with any B-read in this region. The pile below illustrates this effect where it is clear all alignments are interrupted over the interval [2100,2200].

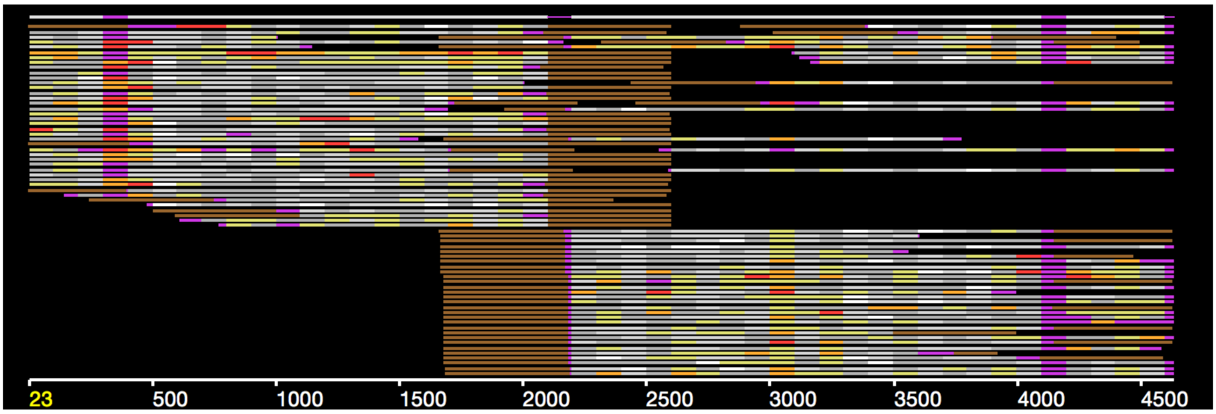

The pile below shows an even larger low quality region that is over 4000bp long. However, one cannot immediately conclude that the segments in question are low quality, as another possibility is that the read is a chimer, and the drop out is due to the presence of the chimer junction. Typically such breaks are small as in the pile above. To distinguish the two possibilities, one needs to see if the B-reads are the same on both sides of the gap and that the gap distance is consistent with the gaps implied in the B-reads. In the pile below, alignment pairs that are so consistent, are indicated by dashed lines across the gap. One sees that about 1/2 the pairs are consistent across the gap. For the others, a careful analysis reveals that the B-reads are not long enough to actually span the 4Kbp gap, terminating before reaching the other side.

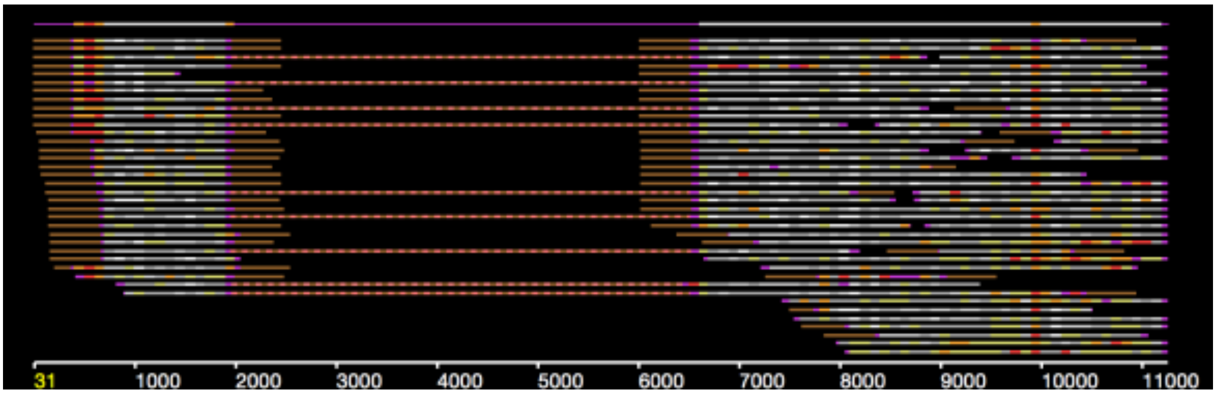

Hopefully, the examples and exposition above make it clear that analyzing read piles is pretty interesting and informative. We have built a complete research prototype pipeline that uses the intrinsic quality values and the piles to trim poor prefixes and suffixes of each read, to detect and break all chimeric reads, to remove all adaptamers missed by the Pacbio software, and to identify and patch low quality gaps. I am still not completely happy with elements of the current scrubbing pipeline and so will not release all of it at this time. However, the intrinsic quality value caller is stable and I am releasing it now, so that at least you can have an error profile for your reads at this time. Perhaps you can build a better scrubber.

The command "`DASqv -c40 Project.db Project.las`" will produce an intrinsic quality track, "qual" in the files `.Project.qual.anno` and `.Project.qual.data`. In this example I was assuming that `Project` entailed 40X sequencing of a target genome and set the parameter -c accordingly. While I could have tried to estimate the coverage of your project by looking at the piles, the assumption was that the user invariably knows this information and so can easily supply it. For a given read, its quality vector has length $\lfloor n/\Delta \rfloor + 1$ where Δ is the trace spacing (default 100), and it consists of one byte numbers between 0 and 50 where

I capped the values at 50 as any segment with worse than an average 50% difference to its B-reads is obviously a hideously bad segment (two *random* DNA string will align with 52% differences). As stated carefully previously, intrinsic quality values are statistics that correlate with the underlying quality, and not the actual error rate of the segment. To help you calibrate this to the actual quality, **DASqv** can be asked to output a histogram of the match values and quality values with the -v option. An example output, might be:

```
DASqv -c40 Project Project
Input:   28,347reads,   213,832,622 bases
Histogram of q-values (average 10 best)
         Input                 QV
50:     124591    0.2%       369417   17.2%
49:              30831            0.0%                1177            0.1%
48:              45052            0.1%                1304            0.1%
47:              55249            0.2%                1318            0.2%
46:              70125            0.3%                1576            0.3%
45:              88451            0.4%                1823            0.4%
44:             109586            0.6%                2019            0.5%
43:             138026            0.8%                2404            0.7%
42:             167315            1.0%                2891            0.8%
41:             206812            1.3%                3271            1.0%
40:             276479            1.7%                3718            1.2%
39:             273300            2.1%                4164            1.4%
38:             363039            2.7%                4507            1.7%
37:             439018            3.3%                5130            2.0%
36:             520598            4.1%                5792            2.3%
35:             629455            5.0%                6352            2.7%
34:             760599            6.1%                6980            3.1%
33:             893687            7.4%                8118            3.5%
32:            1109495            9.0%                9013            4.0%
31:            1309997           10.9%               10322            4.6%
30:            1599547           13.2%               12054            5.3%
29:            1881921           16.0%               14483            6.1%
28:            2230686           19.2%               17300            7.1%
27:            2659619           23.1%               21917            8.3%
26:            3071431           27.6%               27422            9.8%
25:            3660064           32.9%               34941           11.8%
24:            3751121           38.4%               45721           14.3%
23:            4299877           44.7%               58711           17.6%
22:            4550533           51.3%               75977           21.9%
21:            4729179           58.2%               96397           27.3%
20:            4818604           65.2%              118736           34.0%
19:            4445302           71.7%              142094           41.9%
18:            4232805           77.8%              160940           51.0%
17:            3771886           83.3%              172833           60.7%
```

16:	3209555	88.0%	173724	70.4%
15:	2585374	91.8%	161052	79.4%
14:	1974279	94.7%	135985	87.1%
13:	1416910	96.7%	100996	92.7%
12:	958661	98.1%	66090	96.4%
11:	599259	99.0%	37087	98.5%
10:	350258	99.5%	17349	99.5%
9:	185194	99.8%	6601	99.9%
8:	91231	99.9%	1966	100.0%
7:	39498	100.0%	518	100.0%
6:	15779	100.0%	114	100.0%
5:	5154	100.0%	23	100.0%
4:	1630	100.0%	3	100.0%

It is a matter of experience, but my general finding is that of the QV's not capped at 50, about 80% of the data is definitely usable and 5-7% is definitely bad, leaving the rest in a "grey" zone. From the histogram above anything under 22 is definitely usable, and anything over 28 is definitely bad. Future scrubbing commands that trim and patch reads take these two user-selected thresholds as input (a -g and -b parameters) to control their function. In this way, you can scrub harder or softer by adjusting these two thresholds.

To end, the routine **DBdump** now takes a -i option that asks it to output the quality vector for reads, provided, of course, the "qual" track exists.

A developing command guide for the scrubber module is available here.

Scrubbing Reads for Better Assembly

Posted on April 22, 2017 by Gene Myers

In an earlier **post**, I introduced the concept of **intrinsic quality values** — a statistic indicating the quality of every say TP=100bp interval of a read — that could be computed from the **pile** of local alignments for each read computed by **daligner** with the -t option set to TP. This idea is important because of the complex and variable error profile of PacBio long reads which I illustrated with the following hypothetical example:

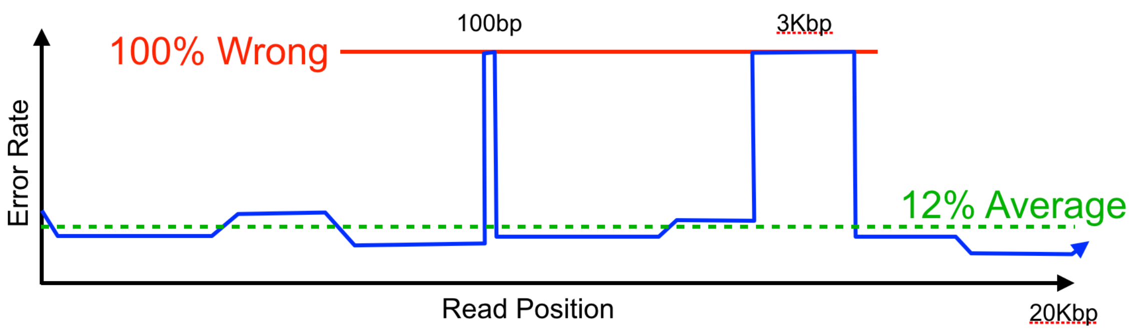

Error profile of a typical long read. The average error rate is say 12% but it varies and occasionally is pure junk.

The quality values together with the pattern of alignments in a read's pile allow one to detect these, sometimes long, low-quality segments of a read, as well as detect when the read is a chimer or contains undetected adaptamer sequence that should have been excised. These artifacts are quite frequent in PacBio data sets and apart from repeats are the major cause of poor assembly. We call the process of repairing the low-quality sequence and removing the chimers and missed adaptamers, **scrubbing**, and formally the goal of a scrubber is to edit a set of reads with as little loss of data as possible so that:

1. every edited read is a contiguous segment of the underlying genome being sequenced, and
2. every portion of a read is of reasonable quality, i.e. <20% error or Q8.

The conditions above are exactly those *assumed* by a string graph construction algorithm and any data that doesn't meet these conditions introduces breaks and false branches in the string graph. For this reason I choose to scrub hard, i.e. my scrubber will over call chimers and adaptamers rather than miss them, so that condition 1 above is meet by all the data after scrubbing.

The scrubber module consists of a pipeline of 4-5 modules as opposed to a single program that performs the end-to-end task. We structured it this way so that one can run each pass in a block-parallel manner on an HPC cluster, and secondarily, so that a creative user can potentially customize or improve aspects of the overall process. The figure below, shows the pipeline:

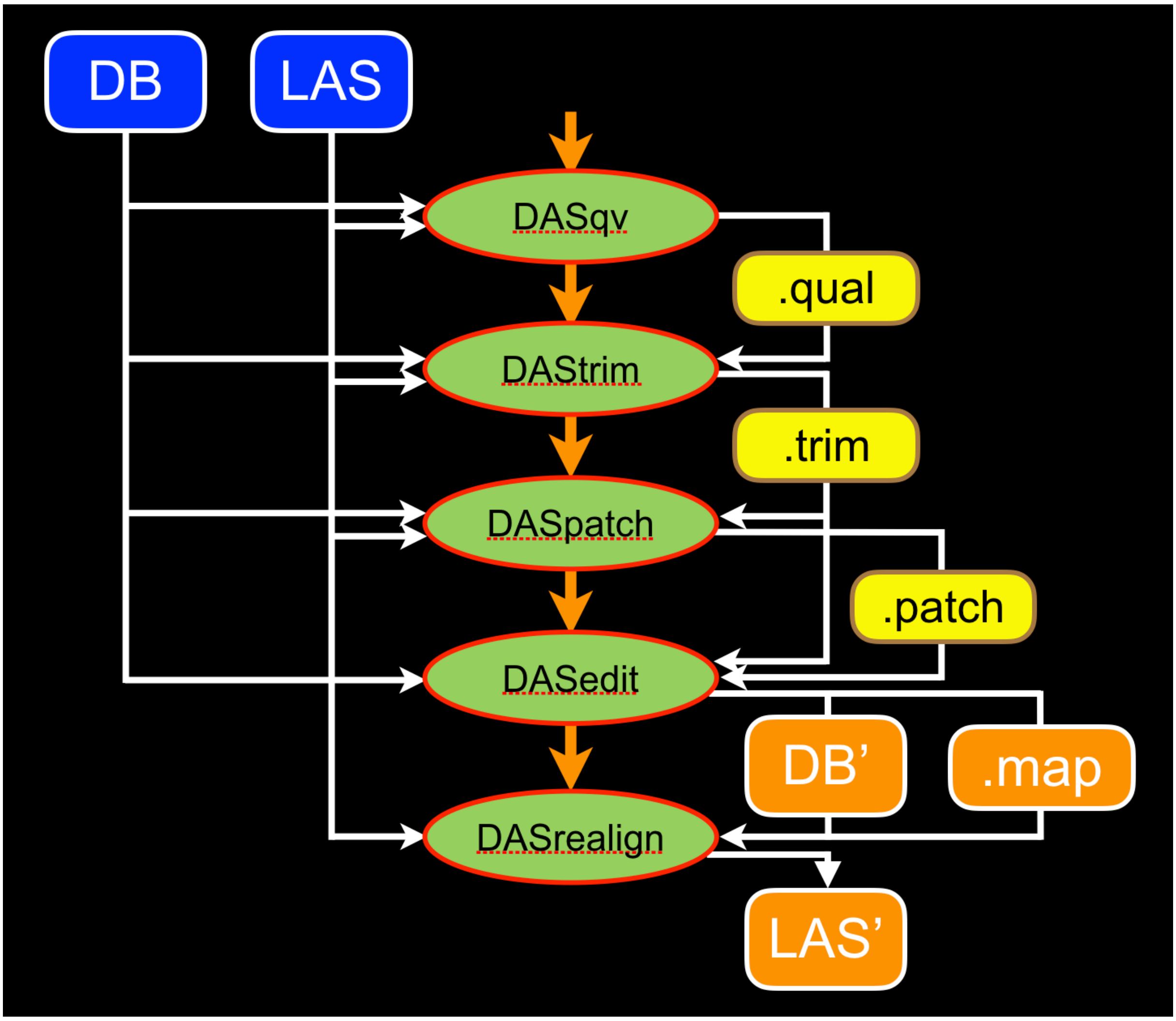

Data flow, including tracks, of the scrubbing pipeline

Each program in the pipeline can be run on the overlap piles for a block of the underlying DB in parallel, allowing a simple HPC parallel scheme. By doing so each program per force outputs only the portion of a track for the intput block (i.e.. *.qual*, *.trim*, *.patch*), and these **block tracks** must be concatenated into a single track for the entire database with **Catrack** before the next step in the pipeline can be performed. Fortunately, this serial step is very fast, basically amounting to concatenating a number of files of total size *O(R)* where *R* is the number of reads in the database.

The first step in scrubbing is to compute the quality values for every read segment and record them in a ***qual*** track with **DASqv** as described in an earlier **post**. Recall that with the -v option **DASqv** displays a histogram of the QV's over the dataset. Using this histogram one should select a threshold ***GOOD*** for which 80% of the QV's are not greater than this value, and another ***BAD*** for which 5-7% of the QV's are not less than this value. In the next two steps of the pipeline, QV's ≤ ***GOOD*** will be considered "(definitley) good", and QV's ≥ ***BAD*** will be considered "(definitely) bad".

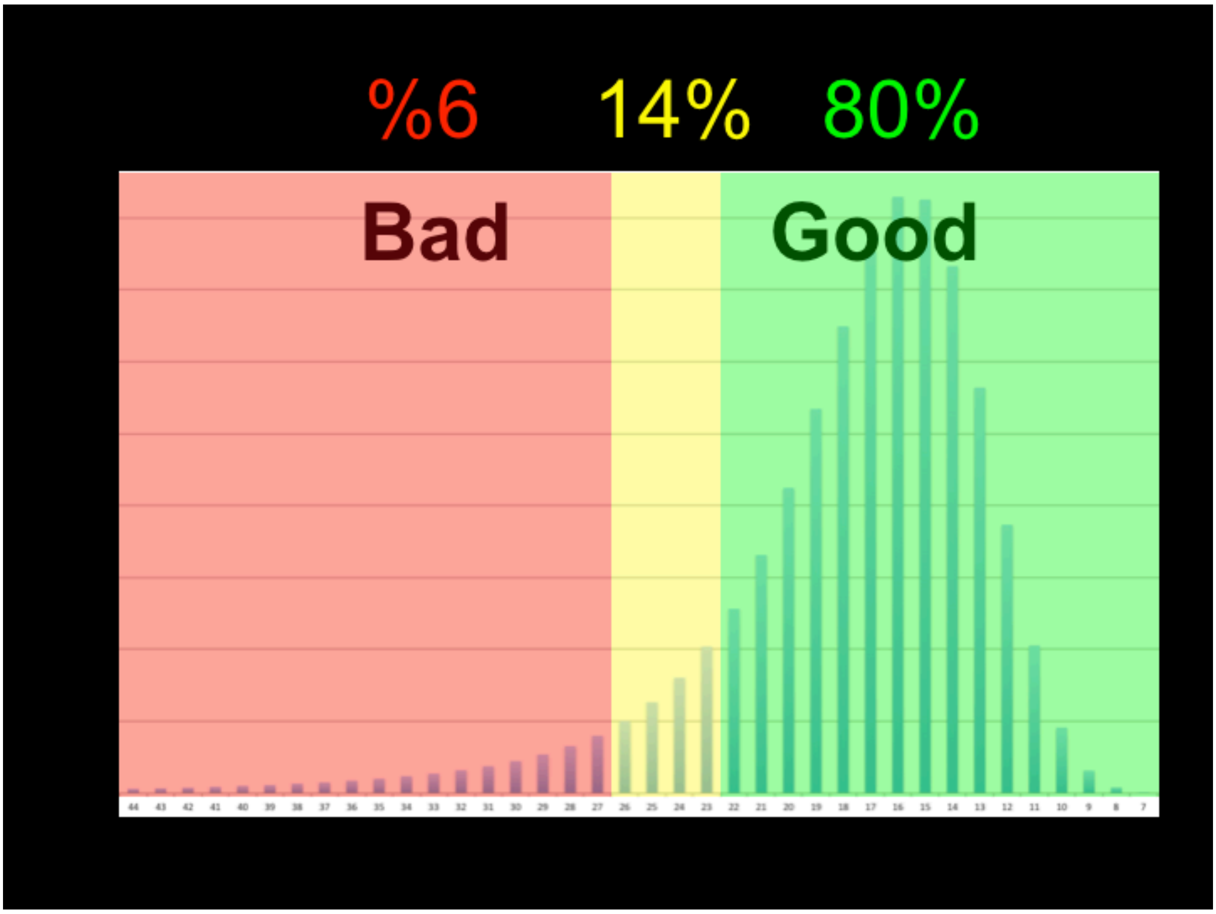

Any QV's between the two thresholds will be considered of unknown quality. Decreasing these values, increases the amount of data that will be scrubbed away as not of sufficient quality, and increasing these thresholds retains more data but at a sacrifice in quality. The percentile guidelines above represent good settings for typical PacBio data (that is about 80% of the data actually is good and 5% of it is actually crap).

The ***GOOD/BAD*** thresholds must be supplied as the **-g** and **-b** parameters to the next phase of the pipeline realized by **DAStrim**. **DAStrim** first uses the quality values and the supplied **-g** and **-b** thresholds to determine a sequence of high-quality (HQ) segments for the A-read of each pile. Formally, an **HQ segment** begins and ends with a good trace point interval interval, contains no bad intervals, and is at least 400bp long. Any intervals before the first HQ segment and after the last HQ segment, are clipped/trimmed from the beginning and end of the read, respectively. The main task of **DAStrim**, is to then determine what to do with the gaps between the HQ segments of a read. There are 4 cases:

1. The gap is completely covered/spanned by lots oflocal alignments with other reads – then the gap is simply low quality sequence and deemed ***LOW-Q***. This is illustrated in the example at right by the leftmost low-quality gap.

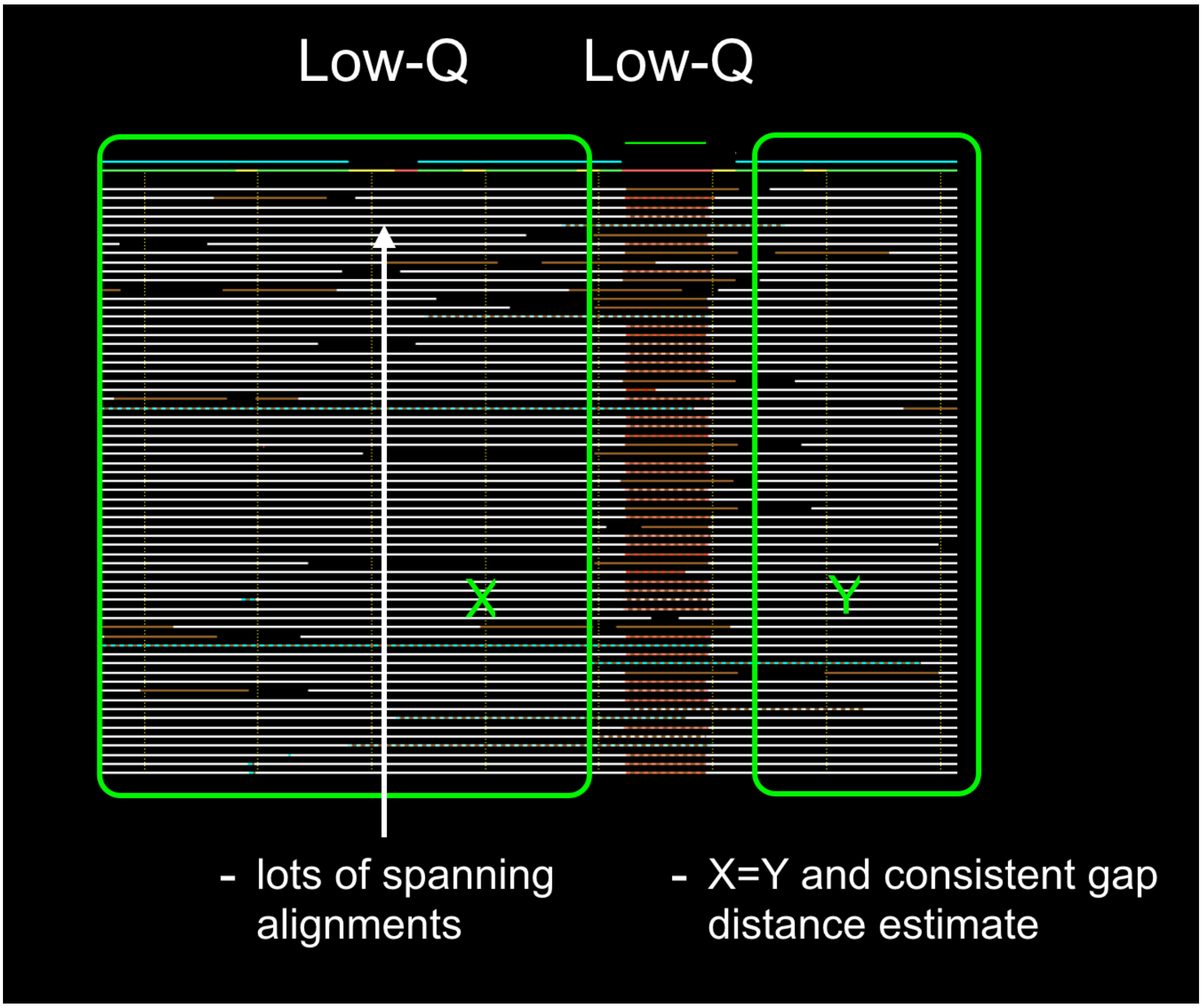

2. The gap is not spanned by local alignments with other reads, but most of the local alignments to the left (X) and right (Y) of the gap are for the same B-read and the distance between the two B-read segments in the relevant local alignments are consistent with the size of the gap – then the gap is a (very) low quality (and often long) drop out in the A-read and the gap is deemed ***LOW-Q***.
3. the gap is not spanned but the left (X) and right (Y) alignment sets have the relevant B-reads in the complement orientation with respect to each other – the gap is due to an ***ADAPTAMER***. Typically, the gap is also very sharp with the gap between paired local alignments being very narrow as in the example below left. In the situation where one or more adatamer gaps is found for a read, all but the longest subread between two of the adaptamers is thrown away.

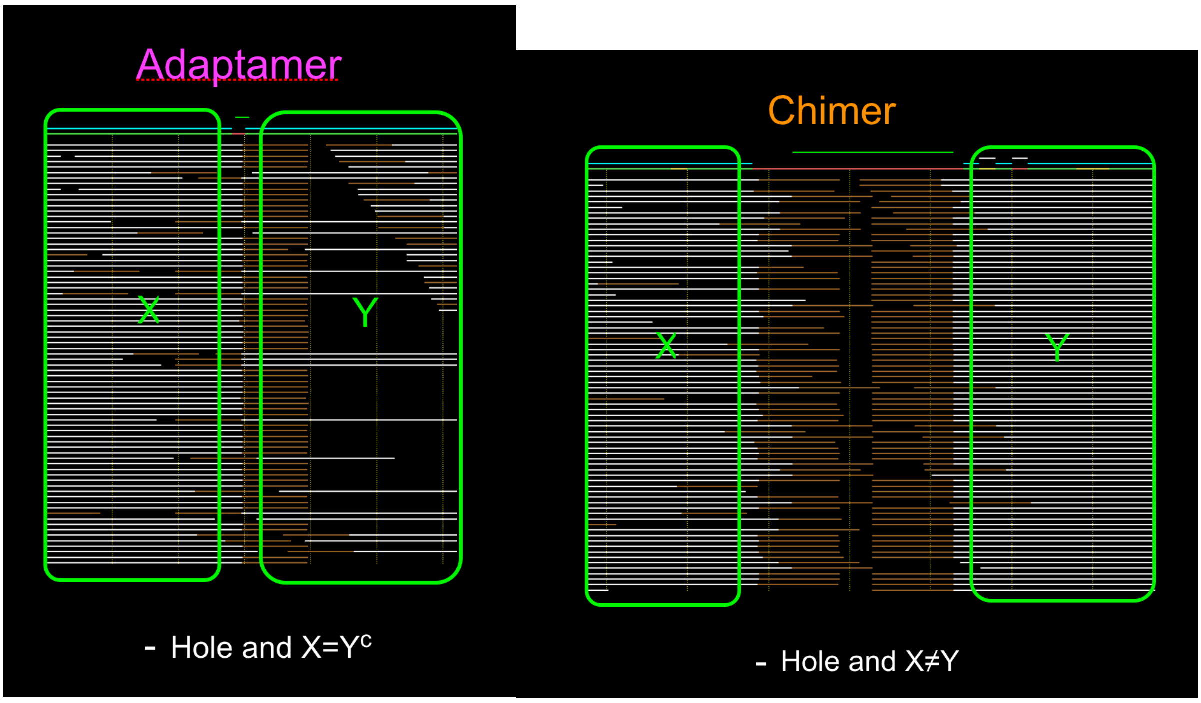

4. the gap is not spanned and there is no correspondence between the reads in the local alignments to the left and right – the gap is due to a chimeric join in the A-read and the gap is designated ***CHIMER*** as illustrated in the example above right. Every chimeric gap splits the A-read into two new reads.

DAStrim outputs a track (or track block) called ***trim*** that for each read encodes the begin/end coordinates for each HQ segment and the classification of each intervening gap (either ***LOW-Q*** or ***CHIMER***). With the -v option it also outputs a report describing how much data was trimmed, designated LOW-Q, etc. In the example below, one sees that the left column is the number of reads or items, and the right column is the total number of bases in those items. 3,117 reads totalling 11.6% of the bases in the data set were found to be either garbage or were totally masked by the **DAMASKER** module and thrown out. Of the remaining reads, 5.7% of the bases were trimmed from the beginning of reads, and 2.5% from the end of reads. 37 missed adaptamer sites were found and 117.8Kbp was trimmed away leaving only the longest sub-read between adaptamers.

```
Input:     22,403 (100.0%) reads      250,012,393 (100.0%) bases
Discard:    3,117 ( 13.9%) reads       29,036,136 ( 11.6%) bases
5' trim:   13,749 ( 61.4%) reads       14,137,800 (  5.7%) bases
3' trim:    7,919 ( 35.3%) reads        6,316,157 (  2.5%) bases
Adapter:       37 (  0.2%) reads          117,816 (  0.0%) bases
Gaps:      35,253 (157.4%) gaps        15,205,800 (  6.1%) bases
Low QV:    30,903 (137.9%) gaps         8,834,200 (  3.5%) bases
Span'd:     3,346 ( 14.9%) gaps         4,441,700 (  1.8%) bases
Chimer:     1,004 (  4.5%) gaps         1,929,900 (  0.8%) bases
Clipped:   25,826 clips                49,607,909 ( 20.6%) bases
Patched:   34,249 patches              13,275,900 (  5.3%) bases
```

In the adaptamer-free reads that were retained, there were 35,253 gaps between HQ segments. 30,903 were deemed ***LOW-Q*** by virtue of being spanned by alignments (Low QV) and 3,346 were deemed ***LOW-Q*** by virture of consistent alignment pairs (Span'd). The remaining 1,004 where deemed to be ***CHIMER***s and will be split creating 1,004 new reads. The last two lines tell one that a total of 20.6% of the bases were lost altogether because of trimming, adapatmers, and chimers, and that 5.3% of the bases were in low-quality gaps that will be patched with better sequence in the ensuing scrubbing passes.

The 3rd phase of scrubbing is realized by **DASpatch** which selects a good B-read segment to replace each ***LOW-Q*** gap with. We call this high-quality sequence a **patch**. The program also reviews all the gap calls in light of the fact that the trimming of reads affects their B-read alignment intervals in the piles of other reads, occasionally implying that a gap should have been called a ***CHIMER*** and not ***LOW-Q***. **DASpatch** further changes a ***LOW-Q*** gap to a ***CHIMER*** call if it cannot find what it thinks is a good B-read segment to patch the gap. The number of these calls is generally very small, often 0, and their number is reported when the -v option is set. **DASpatch** outputs a track (or track block) called ***patch*** that encodes the sequence of B-read segments to use for patching each low quality gap in a read.

The final phase of scrubbing is to perform the patching/editing of reads that is encoded in the ***trim*** and ***patch*** tracks produced in the last two phases, and produce a new data base of all the resulting scrubbed reads. This is accomplished by **DASedit** that takes the name of the original database and the name you would like for the new database as input. This phase runs sequentially and can can be quite slow due to the random access pattern of read sequences required to patch, so you might want to go do something else while it runs. But when its finished you will have a database of reads that are typically 99.99% chimer and adatamer free, and that contain almost no stretches of very low-quality base calls.

The new database does not have a *.qvs* or *.arr* component, that is it is a a sequence or S-database (see the original Dazzler DB post). Very importantly, the new database has exactly the same block divisions as the original. That is, all patched subreads in a block of the new database have been derived from reads of the same block in the original database, and only from those reads. **DASedit** also produces a ***map*** track that for each read encodes the original read in the source DB that was patched and the segments of that read that were high-quality (i.e. not patched). A program **DASmap** output this information in either an easy-to-read or an easy-to-parse format. A small example of a snippet output by **DASmap** is as follows:

```
55 -> 57(2946) [400,2946]
56 -> 58(11256) [700,1900]
57 -> 58(11256) [6600,9900] 83 [10000,11256]
58 -> 59(12282) [400,4100] 88 [4200,9400] 97 [9500,12282]
```

The first line indicates that read 55 in the patched database was derived from read 57 in the original database and is the segment from [400,2946] of that read. Reads 56 and 57 were both derived from read 58 in the original DB, and read 57 consists of segments [6600,9900] and [10000,11256] of read 58 with a patch between them of 83bp (but the source of the patch data is *not* given). The read length of each original read is given for convenience. The purpose of this map is to allow you to map back to the original reads in the final consensus phase of an assembly where one will want to use the original data along with its Quiver or Arrow data encoded in the .qvs/.arr component of the DB.

A database of scrubbed reads can be used in three ways as illustrated in the figure at the end of this paragraph. In the first use-case, a user can simply take the database of scrubbed reads and use them as input to their favorite long-read assembler (e.g. **Falcon** or **Canu**). In our experience doing so *improves* the assemblies produced by these third party systems.

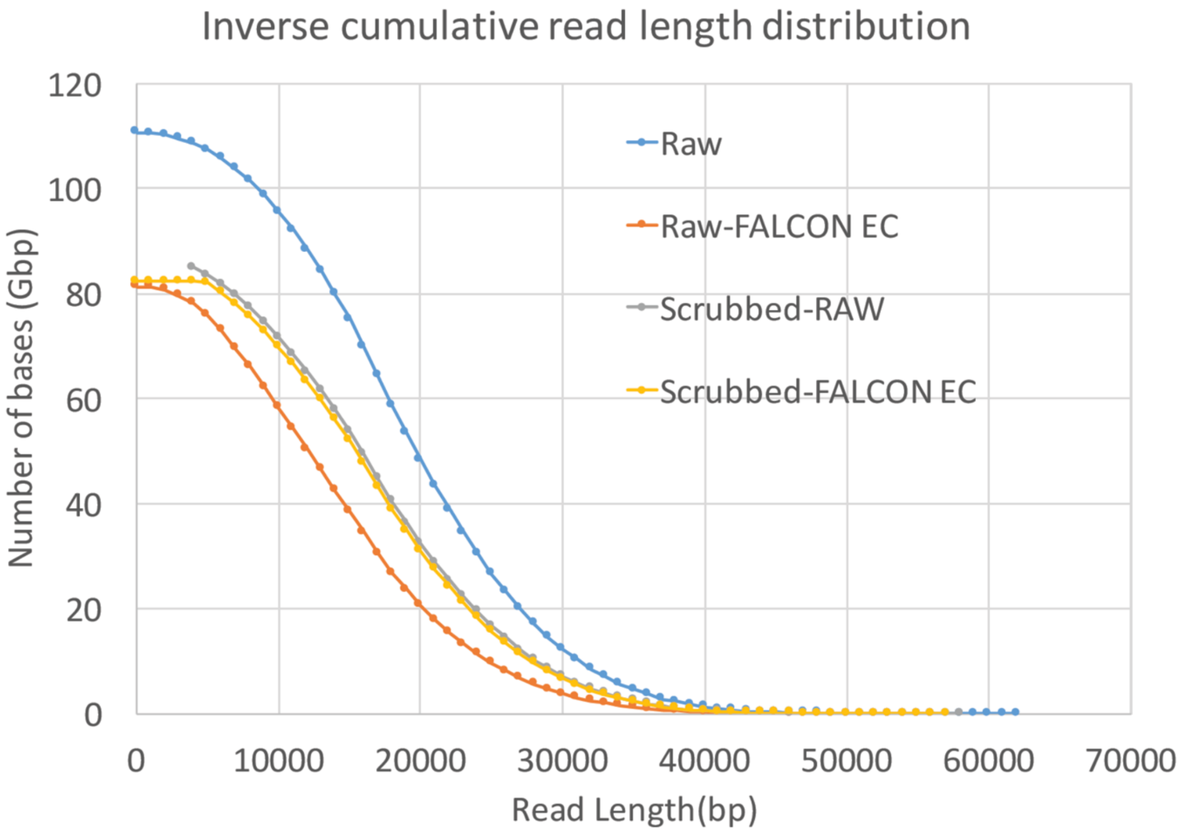

For example, in the plot at right, Falcon's error corrector (EC) tends to break reads at long low quality gaps producing the red line read-length profile given a data set with the blue read-length distribution. When Falcon starts on our scrubbed reads with the grey read-length profile, (which is significantly better than the EC profile), its own error correct leaves these longer scrubbed reads intact. Longer reads into the string graph phase of Falcon's

assembler implies a more coherent assembly, i.e. longer contigs.

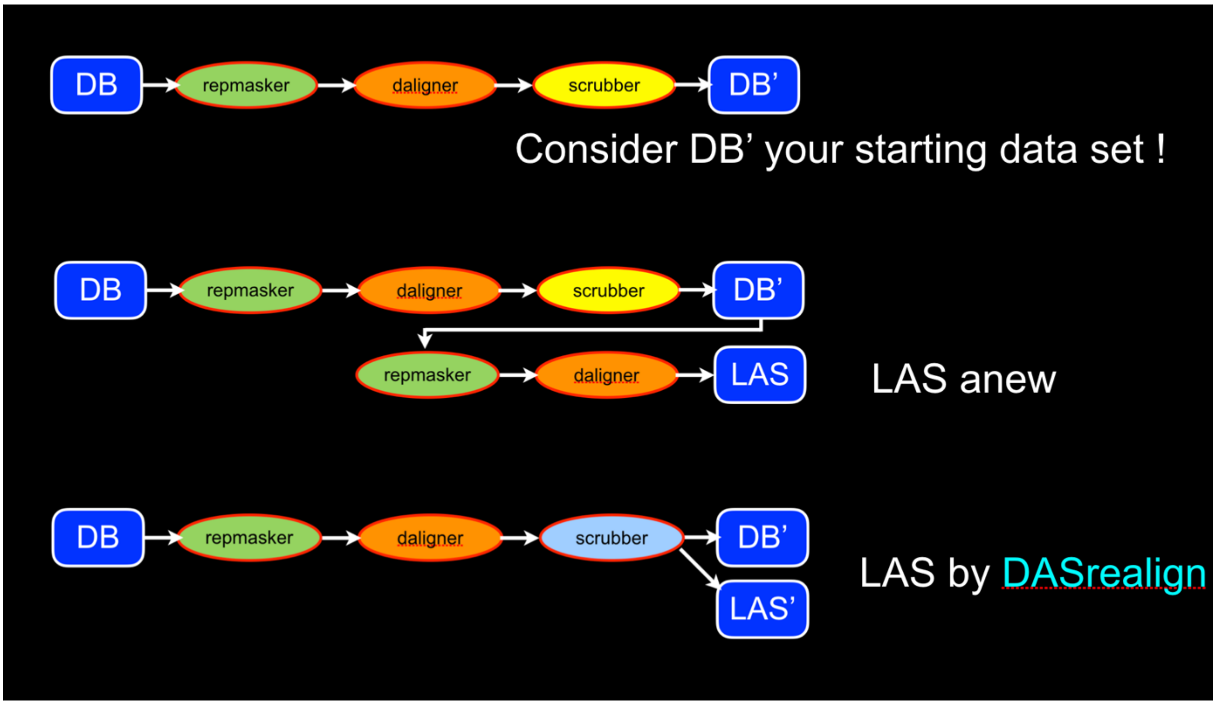

The second use-case, is to rerun the **daligner** on this set of reads to produce *.las* files anew over the scrubbed reads. The down side is that this takes considerable compute time. However, because the reads are scrubbed, one should expect that reads properly *overlap* and a purely local alignments can now be ignored as repeat-induced. Giving the result set of overlaps to a string graph algorithm for assembly should result in better assembly as many repeat-induced correspondences have been removed.

The final third use-case, is rather than re-run daligner, to take advantage of the fact that the piles computed the first time already contain all the pairs of reads that align, and so in fact all that needs to be done is to "re-align" an local alignment found between original reads in terms of the derived reads. This includes extending the local alignment when the introduction of a patch allows it to now extend through a formerly low-quality region. To this end I produced a program **DASrealign** that takes a pair of blocks of the scrubbed DB and the original *.las* file for the block pair (in the original DB), and produces the realigned overlap piles in a *.las* file for the scrubbed DB blocks. Not carefully that this requires (1) that you have ***kept*** the block pair *.las* files produced by the first phase of the **HPC.daligner** process and not removed them, and (2) that after realigning every block pair, you must then merge these to produce the overlap blocks for the scrubbed DB. This process is roughly 40x faster than running **daligner** from scratch. The trade off is that some alignments might be missed that would otherwise be found if **daligner** were rerun on the scrubbed reads, and the trace point encoding of a patched alignment is no longer uniform in the A-read and so one could not scrub the patched reads a second time with the piles so produced. On the otherhand, one can take just the overlaps that are produced this way and perform a string graph assembly.

The pipeline above careful identifies and removes read artifacts such as chimers and adapatamers, and repairs high error-rate read segments, providing a set of reads that will assemble well (without correction). However, ultimately we believe good assembly will further require correction in order to carefully separate haplotypes and low copy number repeats prior to the string graph phase. Therefore, our ultimate ongoing development plan is

to replace **DASedit** with a program that produces error corrected A-reads based on the analysis of **DASqv-DAStrim-DASpatch** and to then realign (at much higher stringency / lower error rate) the resulting reads. We expect this process to take more time than simply applying **DASrealign**, but with the advantage that the reads will also be error corrected. Never the less, just scrubbing reads and feeding them into another assembler is proving to have sufficient value that I decided to release the current pipeline. Best, Gene

Supplementary Text 2

The rationale behind the all-vs-all read alignment step to detect and mask repeats is described in the dazzlerblog (https://dazzlerblog.wordpress.com/2016/04/01/detecting-and-soft-masking-repeats/). For the potential case that this page becomes unavailable in future, we copied the description in this Supplementary Text 2.

Detecting and Masking Repeats

Posted on **April 1, 2016** by **Gene Myers**

The daligner was specifically designed to find local alignments (LAs) between reads as opposed to overlaps where in each end of the alignment reaches an end of one or the other read. This is a necessity because raw PacBio reads can have very low quality or even random segments at any position, and one cannot therefore expect an alignment through these regions. Finding LAs instead of overlaps, allows the Dazzler suite to then analyze **pile-ograms** in order to detect and address these regions (recall that a **pile** for a read *x* is the set of all LAs where *x* is the A-read and a **pile-ogram** is a depiction of all the A-intervals covering *x*). Computing LAs also implies that if a read *x* is from a region of DNA that has a relatively conserved repetitive element in it, then every copy of the element in any read of the shotgun data set will align with the relevant interval of x. For example, given a 50X (=*c*) data set and a repeat that occurs 20 (=*r*) times in the genome, one should expect every occurrence of the repeat in a read to be covered on average by 1,000 (=*rc*) LA's in its pile. The pile-ogram below depicts such a **repeat stack**:

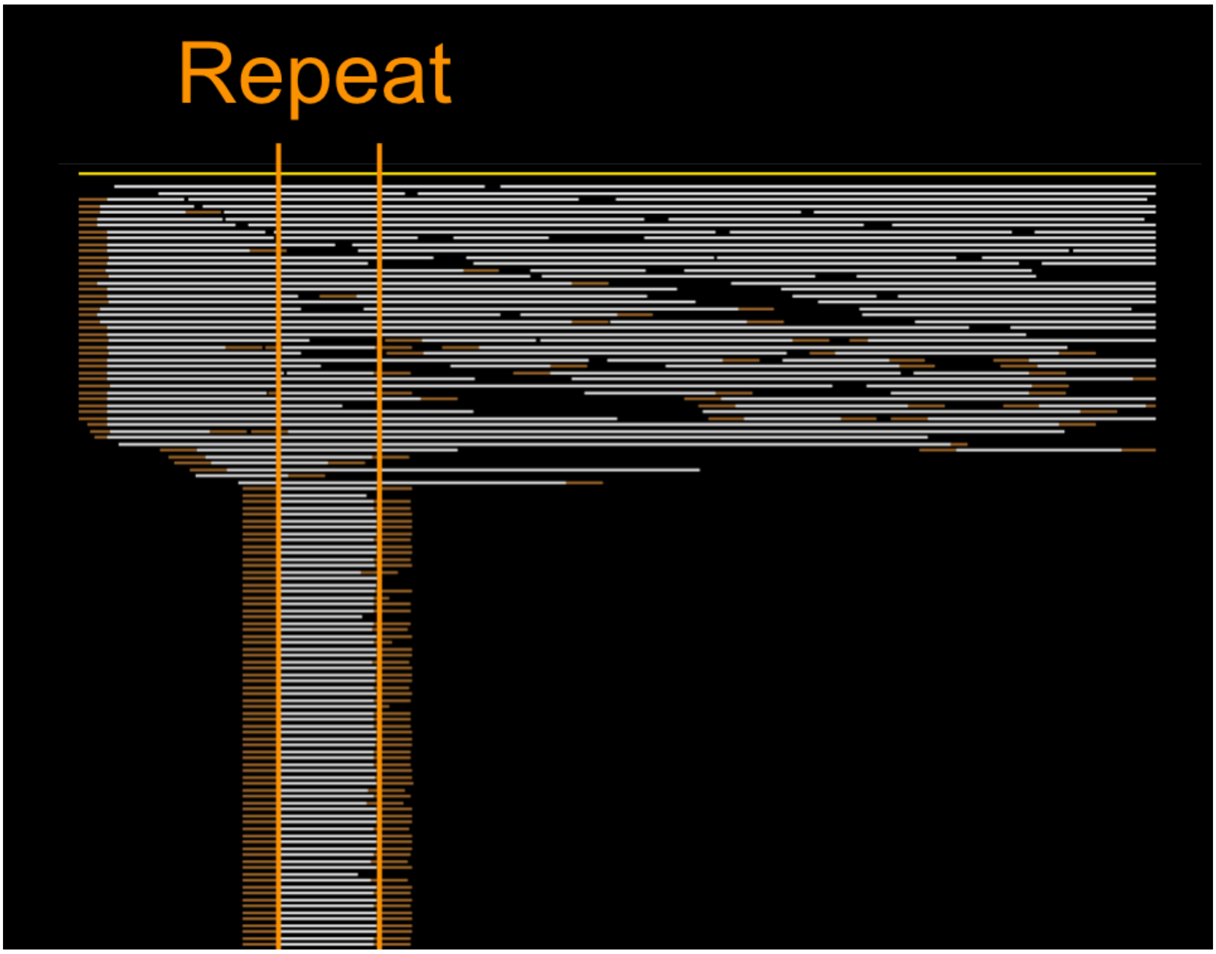

What's good about this is that the excessive depth of "stacked" LA's clearly signals the repetitive element, its approximate copy number, and its approximate boundaries. What's bad about this is that the **daligner** can spend 95% or more of its time finding all the local alignments in these repeat stacks. This observation raises two questions: (a) Is it worth finding them all, and (b) If not, how can we avoid doing so?

The answer to the first question is "no" (i.e., it is not worth finding them all) and our argument is as follows. First recall, that **daligner** can accept any number of [interval tracks] that it uses to **soft mask** the reads. That is, it will only seed/seek alignments in regions that are not masked, but will extend an alignment through a masked region if the the two sequences involved continue to be similar. Next observe, that if a repetitive element is flanked by unique sequence and it is short enough that with high probability there is one or more **spanning reads** that cover the repeat and at least say 1Kbp of the unique flank on either side, then there is no consequence to soft masking this repeat as the spanning reads will provide a coherent assembly across it as shown in the figure below as the left (small) scenario. For Pacbio reads this is generally true of any interspersed repeat of 10Kb or less. If a repeat is compound or so large that there are no spanning reads for it (as shown in the right (large) scenario), then all current assemblers frankly fail to assemble the region involving it, and so in some sense

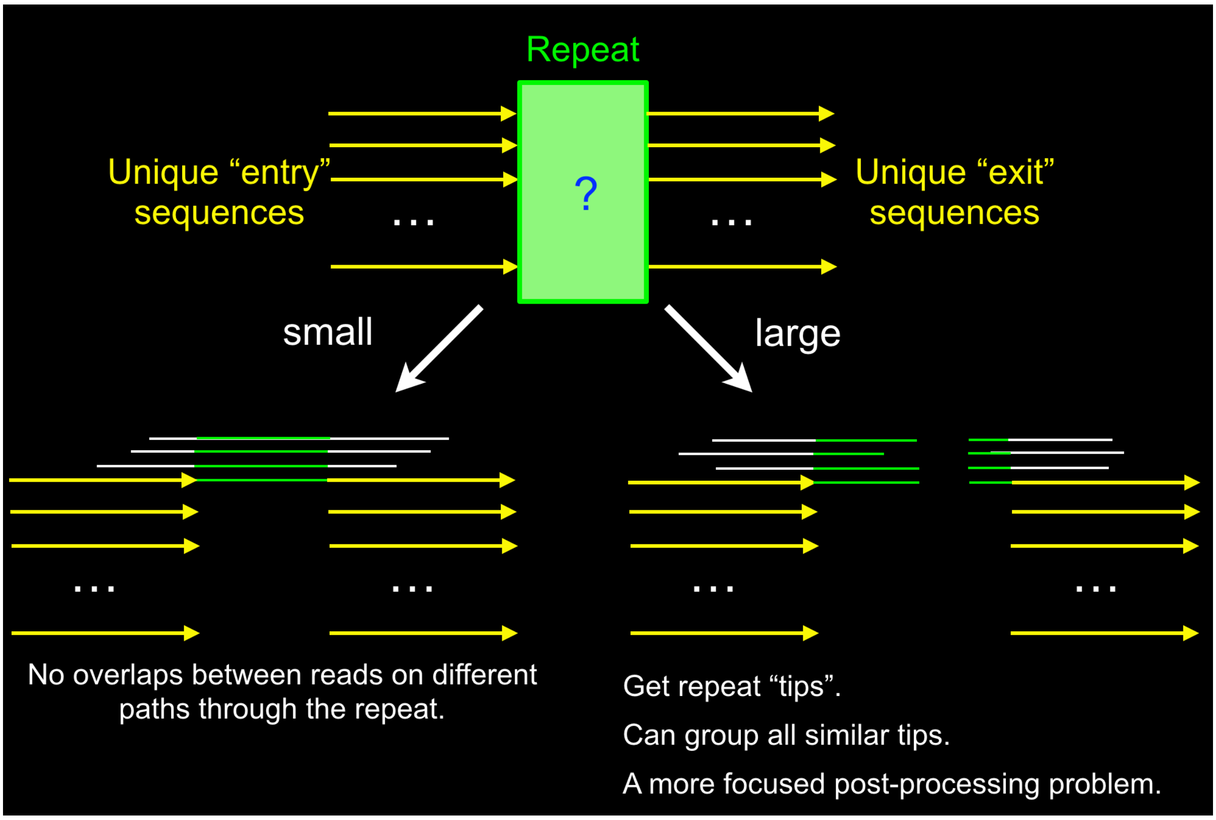

no harm is done by soft-masking the long repeats too. We therefore think that **soft-masking** the repetitive parts of reads is a good initial strategy for the down stream assembly problem: most of the genome will come together with long reads, the "overlap"/**daligner** stage of assembly will be 10 to 20 times faster, and the big repeats can still be solved in a subsequent post-process by analyzing the much smaller number of reads that were completely masked as repetitive.

REPORT THIS AD

So on to the second question, which is how does one efficiently build the needed repeat masks? Consider taking the hypothetical 50X data set above and comparing every 1X block of this data set against itself. First observe that doing so is 2% of all the comparisons that would normally be performed by **daligner**. Second, observe that unique parts of a read will have on average about 1 LA covering them, whereas a segment from a repeat that occurs, say, 10 times in the genome will already have have 10 LA's covering it and will appear as a repeat stack in a read's pile-ogram. The probability of having 10 or more LA's covering a unique segment of a read is exceedingly low, so simply specifying a threshold, such as 10, above which a region is labelled repetitive is an effective strategy for identifying high-copy number repetitive elements. This mask can then be used by the **daligner** when performing the remaining 98% of its comparisons. The resulting suppression of purely repeat-induced LAs will speed things up 10-20 times at almost no degradation in the performance of a downstream assembler like Falcon or the Celera Assembler as argued earlier.

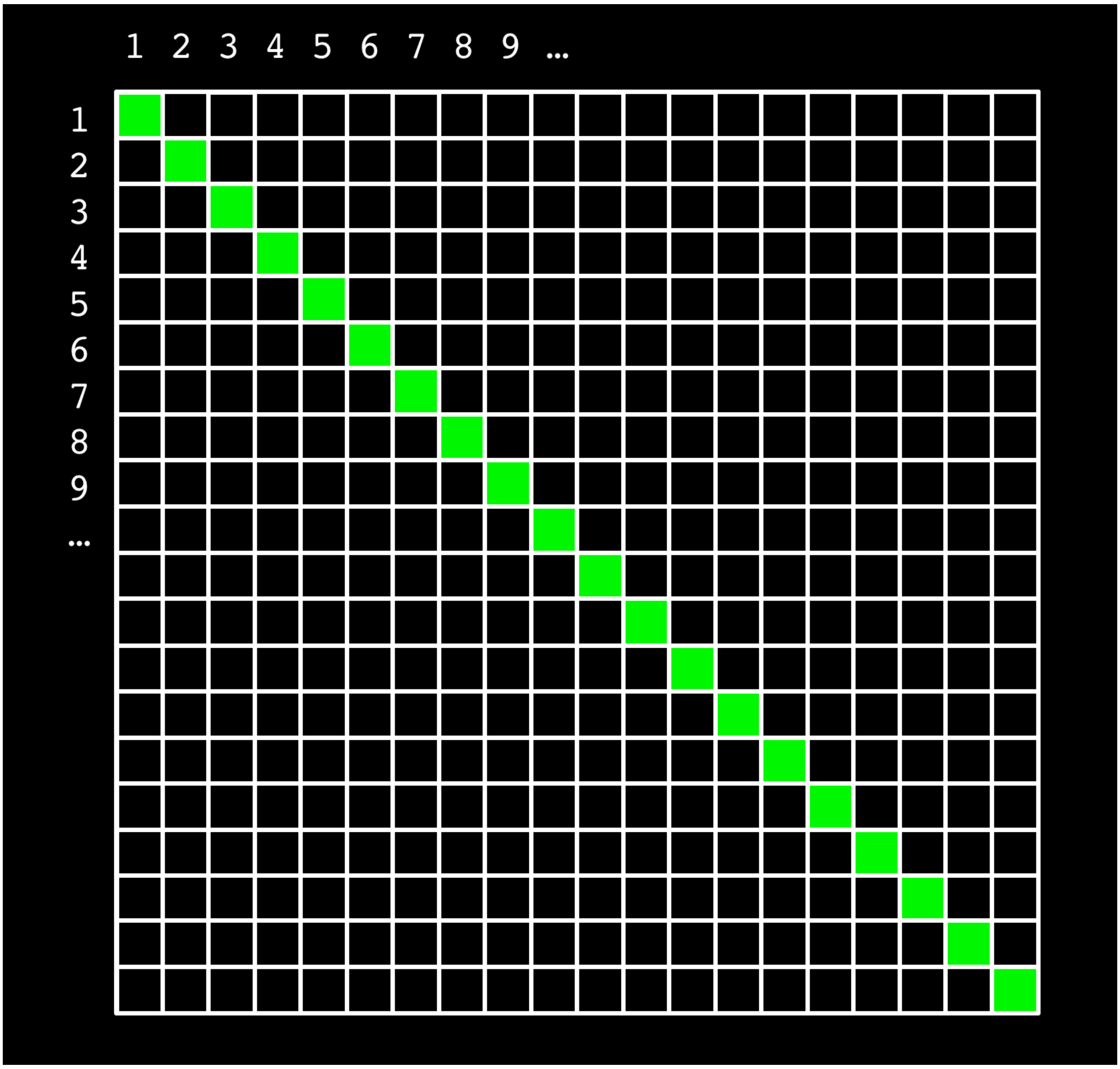

For a very large data set one may want to repeat this process at different coverage levels and with different thresholds in order to achieve greater efficiency. For example, we have a 30X data set of a 30Gbp genome, that requires dividing the Dazzler DB into 3,600 250Mbp blocks. We first run each block against itself (.008X vs .008X) and create a mask *.rep1* with a threshold of 20, that detects only super-repetitive elements. Next we compare every group of 10 consecutive blocks (.08X) against themselves soft-masking with *.rep1*, and create a mask *.rep10* with a threshold of 15 to detect fairly abundant repetitive elements. Then we compare every consecutive group of 100 blocks (.8X) against themselves soft-masking with *.rep1* and *.rep10*, and create a mask *.rep100* with threshold 10 used to further soft-mask the final all-against-all of the 3,600 blocks in the entire data set. The figures interspersed within this paragraph illustrate a 1×1 and 5×5 schema for a hypothetical set of blocks.

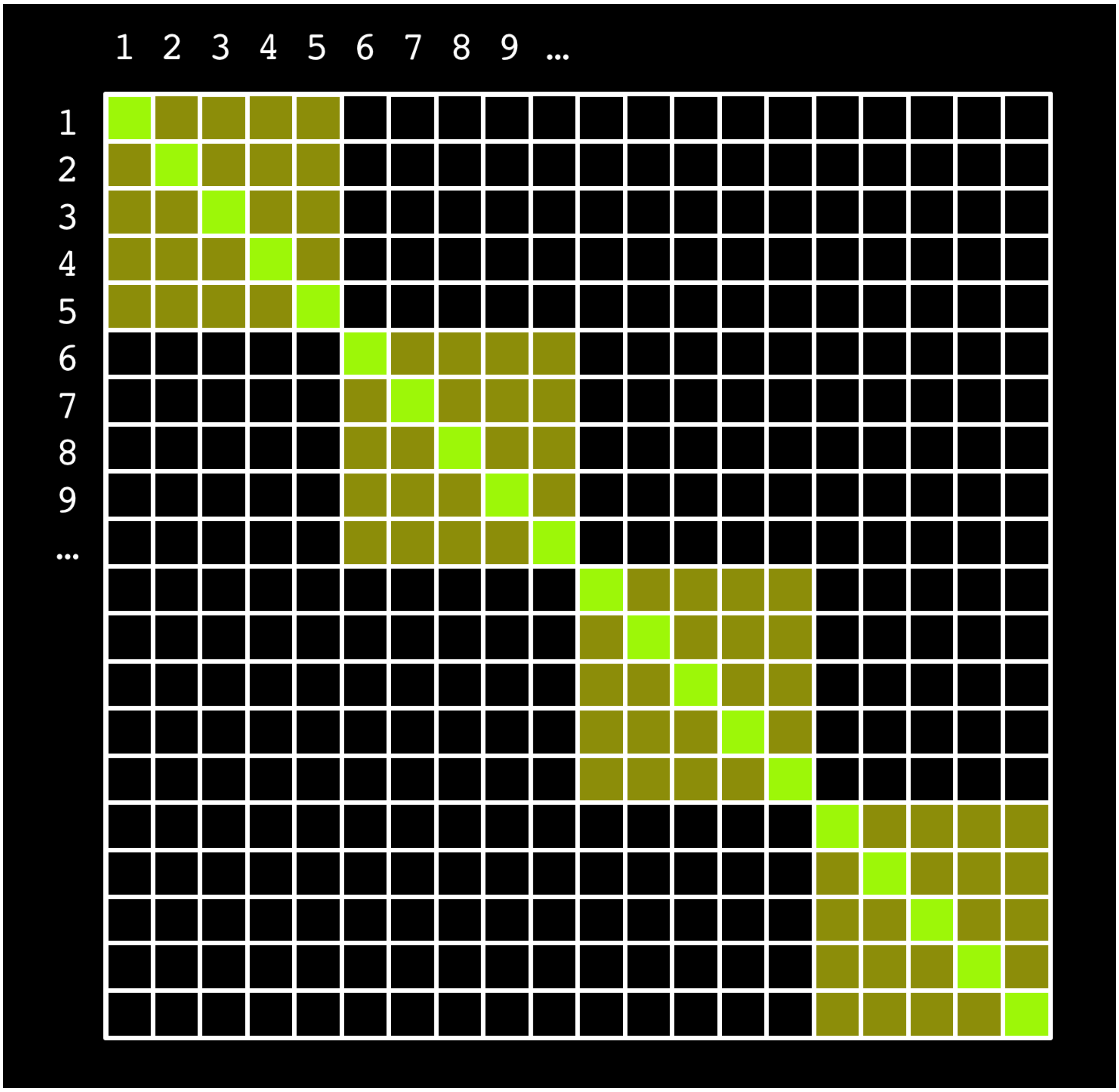

To implement this strategy we have created the programs **HPC.REPmask** and **REPmask** as part of a new **DAMASKER** module. “HPC.REPmask -g$\#_1$ -c$\#_2$” produces a job-based UNIX script suitable for an HPC cluster that:

1. compares each consecutive $\#_1$ blocks against themselves,
2. sorts and merges these into *.las* files for each block, and then
3. calls “REPmask -c$\#_2$ -mrep$\#_1$” on each block and associated *.las* file to produce a block **interval track** or mask, with the name *.rep$\#_1$*, of any read region covered $\#_2$ or more times.

As a running example consider a hypothetical Dazzler data base D that is divided into 150 blocks and contains an estimated 50X coverage of the target genome. Then 3 blocks of data contains 1X of the genome, and to realize a 1X versus 1X repeat mask, we need to compare every consecutive group of 3 blocks against each other and then produce a mask of all reads covered more than say 10 deep. Calling “HPC.REPmask -g3 -c10 D” will produce a shell script to do this, where the script has the following form:

```
# Daligner jobs (150)
daligner D.1 D.1
daligner D.2 D.1 D.2
daligner D.3 D.1 D.2 D.3
daligner D.4 D.4
daligner D.5 D.4 D.5
daligner D.6 D.4 D.5 D.6
…
daligner D.148 D.148
daligner D.149 D.148 D.149
daligner D.150 D.148 D.149 D.150
# Initial sort & merge jobs (450)
LAsort D.1.D.1.*.las && LAmerge L1.1.1 D.1.D.1.*.S.las
LAsort D.1.D.2.*.las && LAmerge L1.1.2 D.1.D.2.*.S.las
LAsort D.1.D.3.*.las && LAmerge L1.1.3 D.1.D.3.*.S.las
LAsort D.2.D.1.*.las && LAmerge L1.2.1 D.2.D.1.*.S.las
LAsort D.2.D.2.*.las && LAmerge L1.2.2 D.2.D.2.*.S.las
LAsort D.2.D.3.*.las && LAmerge L1.2.3 D.2.D.3.*.S.las
…
LAsort D.150.D.150.*.las && LAmerge L1.150.150 D.150.D.150.*.S.las
# Level 1 merge jobs (150)
LAmerge D.R3.1 L1.1.1 L1.1.2 L1.1.3
LAmerge D.R3.2 L1.2.1 L1.2.2 L1.2.3
LAmerge D.R3.3 L1.3.1 L1.3.2 L1.3.3
LAmerge D.R3.4 L1.4.1 L1.4.2 L1.4.3
LAmerge D.R3.5 L1.5.1 L1.5.2 L1.5.3
LAmerge D.R3.6 L1.6.1 L1.6.2 L1.6.3
…
# REPmask jobs (50)
REPmask -c10 -mrep3 D D.R3.1 D.R3.2 D.R3.3
REPmask -c10 -mrep3 D D.R3.4 D.R3.5 D.R3.6
REPmask -c10 -mrep3 D D.R3.7 D.R3.8 D.R3.9
…
REPmask -c10 -mrep3 D D.R3.148 D.R3.149 D.R3.150
# Produce final mask for entirety of D
Catrack D rep3
```

REPORT THIS AD

So the above would pretty much be the end of this post if it were not for one other kind of repetitive element that haunts single-molecule data sets that sample the entire genome including the centromeres and telomeres, namely tandem satellites. Back in the days of Sanger sequencing, such repeats were rarely seen as they were unclonable, but with single molecule reads we see and sample all the genome including these regions. Tandem repeats

are even nastier than interspersed repeats at creating deep stacks in a read's pile-ogram because they align with themselves at every offset of the tandem element as well as with each other! So our masking strategy actually starts by detecting and building a mask for tandem repeats in the data set.

A tandem repeat can be detected in a read by comparing the read against itself, with the slight augmentation of not allowing the underlying alignment algorithm to use the main diagonal of the edit matrix/graph. When a tandem is present, it induces off-diagonal alignments at intervals equal to the tandem element length.

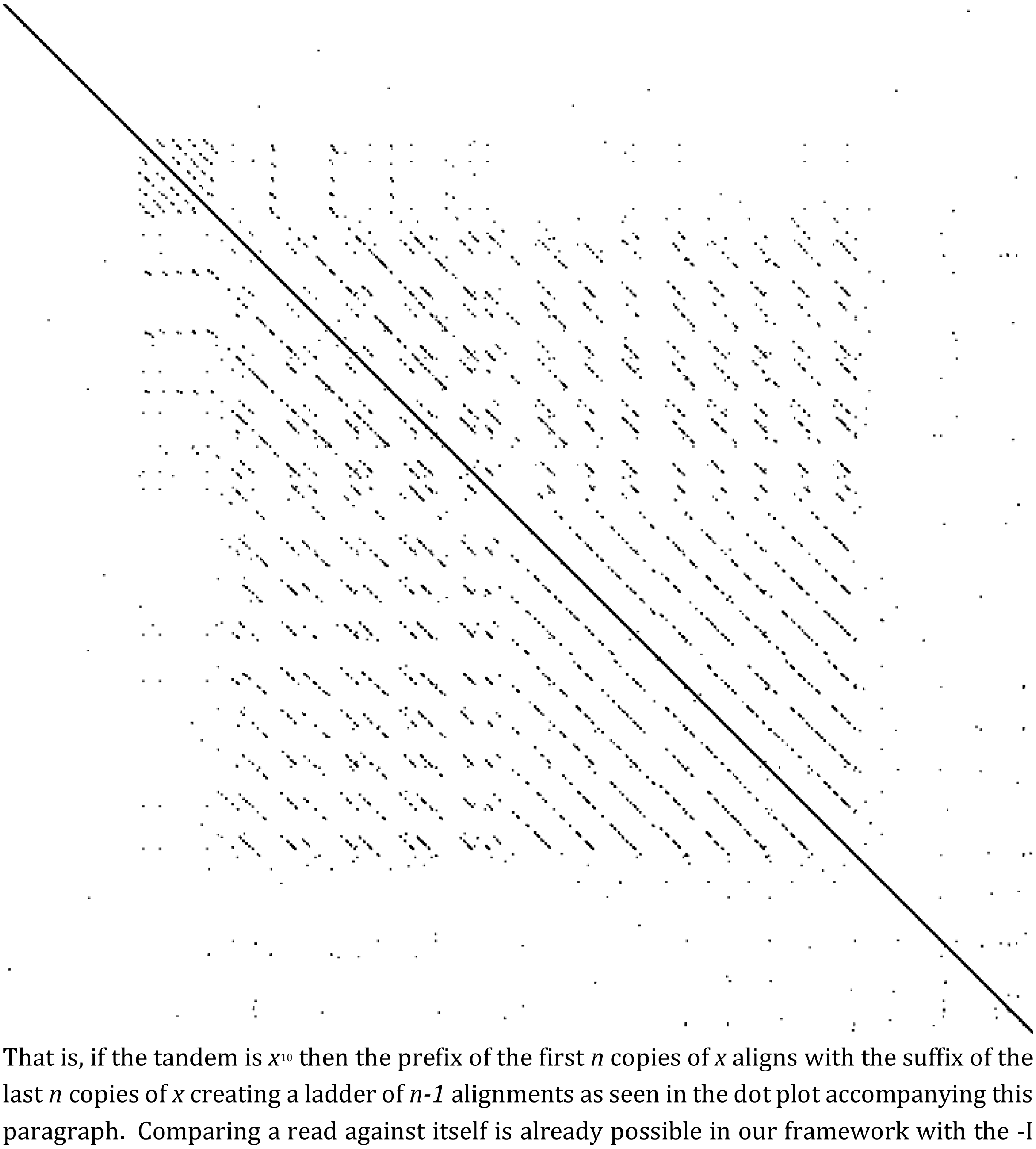

That is, if the tandem is x^{10} then the prefix of the first *n* copies of *x* aligns with the suffix of the last *n* copies of *x* creating a ladder of *n-1* alignments as seen in the dot plot accompanying this paragraph. Comparing a read against itself is already possible in our framework with the -I option to **daligner**. To detect tandems, we built a special version of **daligner** that we call **datander** that given a DB block, compares each read in the block only against itself and outputs these alignments. We further developed the program **TANmask** to use these self-alignments to detect tandem regions to mask: namely, when the two aligned segments of a self-LA overlap, the union of the two segments marks a tandem region. Lastly, the

program **HPC.TANmask** generates a script generator to call **datander**, sort and merge the resulting self-LAs, and finally call **TANmask** on the results. For example, "`HPC.TANmask D`" for the 50X database `D` introduced earlier produces a script of the form:

```
# Datander jobs (38)
datander D.1 D.2 D.3 D.4
datander D.5 D.6 D.7 D.8
…
datander D.149 D.150
# Sort & merge jobs (150)
LAsort D.1.T*.las && LAmerge D.T.1 D.1.T*.S.las
LAsort D.2.T*.las && LAmerge D.T.2 D.2.T*.S.las
…
LAsort D.150.T*.las && LAmerge D.T.150 D.150.T*.S.las
# TANmask jobs (38)
TANmask D D.T.1 D.T.2 D.T.3 D.T.4
TANmask D D.T.5 D.T.6 D.T.7 D.T.8
…
TANmask D D.T.149 D.T.150
# Produce final mask for entirety of D
Catrack D tan
```

One should note carefully, that tandem repeat masking should be performed before the interspersed repeat masking discussed at the beginning. To conclude, we close with the example of the super large 30X database of a 30Gbp genome consisting of 3,600 blocks. Assuming the DB is called, say `Big`, all the scripts for performing the repeat-masked "overlap" phase of assembly would be produced by invoking the commands:

```
HPC.TANmask Big
HPC.REPmask -g1 -c20 -mtan Big
HPC.REPmask -g10 -c15 -mtan -mrep1 Big
HPC.REPmask -g100 -c10 -mtan -mrep1 -mrep10 Big
HPC.daligner -mtan -mrep1 -mrep10 -mrep100 Big
```

REPORT THIS AD

The scripts produced are quite huge for a task of this scale and include a variety of integrity checks and cleanup commands not mentioned here. A newcomer would best be served by gaining experience on a small data set with small blocks, and only proceeding to large production runs with these "HPC.<x>" scripts when one has confidence that they can manage such a pipeline. In a subsequent post on performance/results I will present some timing studies of the impact of repeat masking on the overlap phase of assembly.
