# Supplementary material for: A highly contiguous genome assembly of the bat hawkmoth Hyles vespertilio (Lepidoptera: Sphingidae)
Source: Gigascience. 2020 Jan 23;9(1):giaa001. doi: 10.1093/gigascience/giaa001 (PMC6977585; doi:10.1093/gigascience/giaa001)
Supplement: giaa001_Response_to_Reviewer_Comments_Original_Submission [file giaa001_response_to_reviewer_comments_original_submission.pdf]

Author's Response To Reviewer Comments

Close

We have uploaded a word file that provides a point-by-point response "PointByPointResponse".
The file is labeled as Supplementary Material.

A cover letter to the editor is also uploaded.

Close
