# Supplementary material for: A highly contiguous genome assembly of the bat hawkmoth Hyles vespertilio (Lepidoptera: Sphingidae)
Source: Gigascience. 2020 Jan 23;9(1):giaa001. doi: 10.1093/gigascience/giaa001 (PMC6977585; doi:10.1093/gigascience/giaa001)
Supplement: giaa001_Reviewer_1_Report_Original_Submission — Reuben William Nowell, Ph.D. -- 11/8/2019 Reviewed [file giaa001_reviewer_1_report_original_submission.pdf]

Reviewer Report

Title: A highly contiguous genome assembly of the bat hawkmoth Hyles vespertilio (Lepidoptera: Sphingidae)

Version: Original Submission **Date:** 11/8/2019

Reviewer name: Reuben William Nowell, Ph.D.

Reviewer Comments to Author:

The manuscript of Pippel et al. is clear and concise, with particularly good detail on their gene prediction/annotation pipeline which I think could be very helpful to the community. The genome of the bat hawkmoth they describe seems to me to be well constructed, and, aside from some minor suggestions, I have only two general comments to improve the manuscript.

General comments:

- A general comment that touches on the observed increased genome span of their species relative to the two other Bombycoidea genomes that are available: what exactly are we looking at with the 530 scaffolds spanning 651 Mb? There is no indication that the authors checked that the total span is not influenced by either (a) possible contamination from non-target DNA present in the sample (e.g. cosymbionts, commensals etc) and/or (b) the presence of separately assembled allelic regions caused by uncollapsed heterozygosity in the sequenced individual. Presumably the B. mori and M. sexta assemblies are fully haploid, and so it would be important to compare like with like. A contamination check on the final scaffolds is easy (e.g. blobtools, see comments below). Is it possible to generate a sensible Kmer plot from the sequencing reads that have been filtered during the Dazzler "read patching" process? This would provide at least a feel for what the genome-wide heterozygosity looked like. Alternatively/additionally, an analysis of read coverage of the final scaffolds would also be informative: a fully haploid assembly would show a unimodal coverage distribution; a mosaic assembly comprised of haploid and diploid (uncollapsed) regions would be bimodal.
- I would like to see a table of BUSCO scores in full, for both the genome and the proteome they produce. The duplication score would be informative regarding the first comment above (if there is a large % uncollapsed heterozygosity, the duplication score might reflect this). This could easily be added as another Supp Table.

Minor comments (some of them are encompassed by the general comments):

- Line 61: sentence is too long, suggest to break it somewhere after "excellent flying abilities."
- Line 111: include the DAZZLER discussion/pipeline at https://dazzlerblog.wordpress.com/ as an Supp Mat in gigaDB somehow? It seems like a potentially useful resource, that could go missing in the future
- Line 112: "of" -> "is"
- Line 115: "substantially larger by 191 and 232 Mb" - are we sure the increased length is not contributed by contamination of DNA from non-target organisms present in the sample? I am sure this is not the case, but a simple analysis of the final scaffolds (e.g. blobtools, https://github.com/DRL/blobtools) would be informative
- Line 115: alternatively, the larger increase could be driven by the presence of allelic regions that are

separately assembled and both present in the genome assembly. Is it possible to show a distribution of coverage of the scaffolds? Collapsed regions will show 2X coverage but uncollapsed regions will show 1X coverage. The presence of uncollapsed regions might also affect the Duplication score of BUSCO analysis, which is not provided (see below). Overall, the authors could do a tiny bit more to convince me the increase in genome span is not driven by either (a) contamination and/or (b) the presence of uncollapsed allelic regions. A Kmer distribution from the raw reads would also be useful to ascertain a general level of heterozygosity of this organism

- Line 138: "much" -> "many"
- Line 167: please include the complete BUSCO scores, e.g. C,[S,D],F,M, as there is some important information particularly with potential duplication of BUSCO genes
- Line 167: in addition, what are the BUSCO scores for the genome, i.e. run using "-mode genome" vs the genome scaffolds?
- Line 281: again, I suggest the wordpress HTML or text therein be deposited as a Supp Mat for the paper, in case the blog post is lost in the future
- Line 361 and others: what is the rationale for the different Evidence Modeler weightings provided by the various alternative methods? E.g. gene projections are given 8, protein homology 2, etc… why? It would be useful for others if the justification was explained
- Line 372: what proportion of the RNA-seq reads from the "closely related" H. euphorbiae mapped to the H. vespertilio genome? The gene annotation of this species is very thorough and so I don't suggest that the authors should have to do this, but it would be interesting to know what the annotation result is using the BRAKER2 pipeline (https://github.com/Gaius-Augustus/BRAKER), if the % RNA-seq reads mapping across the two species is reasonably high
- Line 419: see above for general comment on BUSCO - e.g., what is the size of the Endopterygota gene set?

Level of Interest

Please indicate how interesting you found the manuscript: Choose an item.

Quality of Written English

Please indicate the quality of language in the manuscript: Choose an item.

Declaration of Competing Interests

Please complete a declaration of competing interests, considering the following questions:

- Have you in the past five years received reimbursements, fees, funding, or salary from an organisation that may in any way gain or lose financially from the publication of this manuscript, either now or in the future?
- Do you hold any stocks or shares in an organisation that may in any way gain or lose financially from the publication of this manuscript, either now or in the future?

- Do you hold or are you currently applying for any patents relating to the content of the manuscript?
- Have you received reimbursements, fees, funding, or salary from an organization that holds or has applied for patents relating to the content of the manuscript?
- Do you have any other financial competing interests?
- Do you have any non-financial competing interests in relation to this paper?

If you can answer no to all of the above, write 'I declare that I have no competing interests' below. If your reply is yes to any, please give details below.

I declare that I have no competing interests

I agree to the open peer review policy of the journal. I understand that my name will be included on my report to the authors and, if the manuscript is accepted for publication, my named report including any attachments I upload will be posted on the website along with the authors' responses. I agree for my report to be made available under an Open Access Creative Commons CC-BY license (http://creativecommons.org/licenses/by/4.0/). I understand that any comments which I do not wish to be included in my named report can be included as confidential comments to the editors, which will not be published.

Choose an item.

To further support our reviewers, we have joined with Publons, where you can gain additional credit to further highlight your hard work (see: https://publons.com/journal/530/gigascience). On publication of this paper, your review will be automatically added to Publons, you can then choose whether or not to claim your Publons credit. I understand this statement.

Yes Choose an item.
