# Supplementary material for: A highly contiguous genome assembly of the bat hawkmoth Hyles vespertilio (Lepidoptera: Sphingidae)
Source: Gigascience. 2020 Jan 23;9(1):giaa001. doi: 10.1093/gigascience/giaa001 (PMC6977585; doi:10.1093/gigascience/giaa001)
Supplement: giaa001_Reviewer_2_Report_Original_Submission — Simon Baxter -- 11/23/2019 Reviewed [file giaa001_reviewer_2_report_original_submission.pdf]

Reviewer Report

Title: A highly contiguous genome assembly of the bat hawkmoth Hyles vespertilio (Lepidoptera: Sphingidae)

Version: Original Submission **Date:** 11/23/2019

Reviewer name: Simon Baxter

Reviewer Comments to Author:

Nuclear and mitochondrial genomes of the bat hawkmoth were sequenced using PacBio technology and assembled using "a customized assembler called DAmar". This produces impressive assembly statistics [651.4 Mb assembly, 530 contigs, N50 = 7.5 Mb]. In summary, this manuscript is well written and presents an important genome with a very useful accompanying browser.

Concerns and comments

1. Chromosomal scaffolding was not performed, which is very useful for comparative genomics.
2. If this is the first time DAmar has been used in publication, I would recommend a brief explanation in the results and a comparison against one or more available assemblers (e.g. Flye). Is there an advantage using DAmar to assemble lepidopteran genomes over other assemblers? It is important to justify.
3. "11,721 and 19,760 gene predictions that were projected from B. mori and M. sexta, respectively". I'm interested to know how many of the gene predictions were shared between the two.
4. RNAseq data from Hyles euphorbiae was used in one of the steps to develop gene models. It wasn't clear why H. vespertilio mRNA sequence wasn't used? Although an extensive and detailed approach was used to develop gene models, it is not clear if this is better than using RNAseq from H. vespertilio to begin with.
5. Line 164. There were 94.5% of BUSCO genes identified, but it should be made clear not all were single copy.
6. Discussion lines 183-204. This is largely a repetition of the results. I would prefer to see some discussion explaining why 11,721 and 19,760 â€¨CESAR predicted genes were identified from B. mori and M. sexta, and then 24567 and 7102 were identified with the GenomeThreader Lepbase gene set. Why are there more M. sexta in the CDS? Why are there 154299 Bicyclus hits and what is the repeat?
7. Figure 2B. This presents a treemap comparison of the H. vespertilio MARVEL assembly - do you mean the DAmar assembly?

Level of Interest

Please indicate how interesting you found the manuscript: Choose an item.

Quality of Written English

Please indicate the quality of language in the manuscript: Choose an item.

Declaration of Competing Interests

Please complete a declaration of competing interests, considering the following questions:

- Have you in the past five years received reimbursements, fees, funding, or salary from an organisation that may in any way gain or lose financially from the publication of this manuscript, either now or in the future?
- Do you hold any stocks or shares in an organisation that may in any way gain or lose financially from the publication of this manuscript, either now or in the future?
- Do you hold or are you currently applying for any patents relating to the content of the manuscript?
- Have you received reimbursements, fees, funding, or salary from an organization that holds or has applied for patents relating to the content of the manuscript?
- Do you have any other financial competing interests?
- Do you have any non-financial competing interests in relation to this paper?

If you can answer no to all of the above, write 'I declare that I have no competing interests' below. If your reply is yes to any, please give details below.

I declare that I have no competing interests

I agree to the open peer review policy of the journal. I understand that my name will be included on my report to the authors and, if the manuscript is accepted for publication, my named report including any attachments I upload will be posted on the website along with the authors' responses. I agree for my report to be made available under an Open Access Creative Commons CC-BY license (http://creativecommons.org/licenses/by/4.0/). I understand that any comments which I do not wish to be included in my named report can be included as confidential comments to the editors, which will not be published.

Choose an item.

To further support our reviewers, we have joined with Publons, where you can gain additional credit to further highlight your hard work (see: https://publons.com/journal/530/gigascience). On publication of this paper, your review will be automatically added to Publons, you can then choose whether or not to claim your Publons credit. I understand this statement.

Yes Choose an item.
