# Supplementary material for: A highly contiguous genome assembly of the bat hawkmoth Hyles vespertilio (Lepidoptera: Sphingidae)
Source: Gigascience. 2020 Jan 23;9(1):giaa001. doi: 10.1093/gigascience/giaa001 (PMC6977585; doi:10.1093/gigascience/giaa001)
Supplement: giaa001_Supplemental_Files [file CoverLetter.pdf › CoverLetter.pdf]

Dr. Michael Hiller
Senior Research Group Leader
Max Planck Institute of Molecular Cell Biology and Genetics
Pfotenhauerstr. 108, Dresden, Germany
Email: hiller@mpi-cbg.de
Phone: +49 351 210-2781
https://www.mpi-cbg.de/hiller

Dec 20th, 2019

Dear Dr. Nogoy,

Thank you very much for considering a revised version of our manuscript "A highly contiguous genome assembly of the bat hawkmoth *Hyles vespertilio* (Lepidoptera: Sphingidae)".

We would like to thank all both reviewers for their helpful comments. We have now addressed all points raised and revised the manuscript accordingly. In particular, we

- show that neither contamination nor alternative haplotypes can explain why the Hyles assembly is substantially larger compared to other Bombycoidea (instead transposon expansions explain a substantial part of this genome size expansion),
- provide a full BUSCO analysis, computed now both for genome and proteome mode,
- show that the majority of gene projections from *B.mori* and *M. sexta* overlap, supporting the validity of these gene evidences.

We added new Supplementary Tables 1, 4, 5 and a Supplementary Figure 3. Text changes are highlighted in red font in the manuscript. We also made smaller text changes and made minor improvements to Figure 4 to present the four types of gene evidences used in our annotation in a consistent order throughout the text.
Our point-by-point response to the comments raised by the reviewers is uploaded as a separate Word document.

We hope that our revised manuscript is now acceptable for publication. We look forward to hearing from you.

Happy Holidays.
Sincerely,

Michael Hiller
