# Supplementary material for: A highly contiguous genome assembly of the bat hawkmoth Hyles vespertilio (Lepidoptera: Sphingidae)
Source: Gigascience. 2020 Jan 23;9(1):giaa001. doi: 10.1093/gigascience/giaa001 (PMC6977585; doi:10.1093/gigascience/giaa001)
Supplement: giaa001_Supplemental_Files [file giaa001_supplemental_files.zip › PointbyPointResponse.docx]

**Point by point response**

**Reviewer #1:**

The manuscript of Pippel et al. is clear and concise, with particularly good detail on their gene prediction/annotation pipeline which I think could be very helpful to the community. The genome of the bat hawkmoth they describe seems to me to be well constructed, and, aside from some minor suggestions, I have only two general comments to improve the manuscript.

We thank the reviewer for these kind words.

**General comments:**

- A general comment that touches on the observed increased genome span of their species relative to the two other Bombycoidea genomes that are available: what exactly are we looking at with the 530 scaffolds spanning 651 Mb? There is no indication that the authors checked that the total span is not influenced by either (a) possible contamination from non-target DNA present in the sample (e.g. cosymbionts, commensals etc) and/or (b) the presence of separately assembled allelic regions caused by uncollapsed heterozygosity in the sequenced individual. Presumably the B. mori and M. sexta assemblies are fully haploid, and so it would be important to compare like with like. A contamination check on the final scaffolds is easy (e.g. blobtools, see comments below). Is it possible to generate a sensible Kmer plot from the sequencing reads that have been filtered during the Dazzler "read patching" process? This would provide at least a feel for what the genome-wide heterozygosity looked like. Alternatively/additionally, an analysis of read coverage of the final scaffolds would also be informative: a fully haploid assembly would show a unimodal coverage distribution; a mosaic assembly comprised of haploid and diploid (uncollapsed) regions would be bimodal.

To address whether larger size of the Hyles assembly can be explained by contamination, we first used mash (<https://www.ncbi.nlm.nih.gov/pubmed/31690338>). Mash is a k-mer based toolkit and estimates the resemblance between genomes and metagenomes. Applying mash to the read data of the six PacBio SMRT cells gives the following heatmap that provides no evidence for any major contamination (sample or library contamination in one SMRT cell would show up as white color compared to the other SMRT cells).

As suggested, we also performed a contamination analysis with BlobTools. This analysis is shown in a new Supplementary Table 4 and we added the following to the text:

“To assess potential contamination, we used BlobTools [29] with default parameters except “max_target_seqs 10” in the blastn step and the NCBI nt database (2019/10/31) for the taxonomy classification step. As shown in Supplementary Table 4, BlobTools classified only 2.17 Mb (0.33% of the of 651.4 Mb assembly) as contamination, showing that contamination is not a major issue that could explain the genome size expansion compared to other Bombycoidea. “

Thus, contamination cannot explain why the *Hyles* assembly is 191 Mb larger than *B. mori*.

A direct k-mer based approach (e.g. using GenomeScope) to estimate genome size and heterozygosity rate from the set of reads is to the best of our knowledge not possible with raw PacBio reads, as the raw reads contain too many base errors. While the DAmar read patching step removes larger sequencing artefacts that can cause premature alignment breaks, such as chimers, missed adapters and long noisy read segments, the resulting reads still have roughly a 12% error rate on average.

As suggested, we performed a read coverage analysis of the final scaffolds. Using the tool purge_dups (<https://github.com/dfguan/purge_dups>), which is also part of the VGP assembly pipeline, we obtained a coverage plot where the major peak is consistent with our sequencing coverage (new Supplementary Figure 3). We also observed a small ‘hump’ at 20X coverage, suggesting that some contigs may be alternative haplotypes. Therefore, we used purge_dups to detect putative alternative haplotypes based on read coverage. This analysis is described in the text:

“To investigate whether some contigs may represent alternative haplotypes, we determined and plotted the per base read coverage. As shown in Supplementary Figure 3, this revealed a large peak around 40X, which is consistent with our sequencing coverage, and a small hump around 20X, indicating that some contigs may be alternative haplotypes. Therefore, we used purge_dups [30] to detect alternative haplotypes based on read coverage. This tool assigned 622.7 Mb (95.6% of the 651.4 Mb assembly) as the purged primary assembly and assigned 28.7 Mb (4.4% of the assembly) as alternative haplotypes (Supplementary Table 5). Haplotig contigs mostly small and often repeat-rich, which complicates accurate read coverage determination as unique read mappings are harder to obtain. Furthermore, repeating the BUSCO analysis on the purged primary assembly resulted in a smaller percentage of complete but duplicated genes (0.3% vs. 2.8%) but also a 0.5% decrease in the total number of complete BUSCO genes. This suggests that while some contigs are indeed alternative haplotypes, others contain unique genes and should not be classified as alternative haplotypes. Overall, this analysis shows that alternative haplotypes cannot explain the larger genome size of *H. vespertilio* compared to other Bombycoidea.”

In summary, neither contamination nor alternative haplotypes can together explain why our *Hyles* assembly is 191 and 232 Mb larger than the *B. mori* and *M. sexta* assembly, respectively. Instead, as described below, an expansion of repeats and other transposons explains a substantial part of this genome size expansion.

- I would like to see a table of BUSCO scores in full, for both the genome and the proteome they produce. The duplication score would be informative regarding the first comment above (if there is a large % uncollapsed heterozygosity, the duplication score might reflect this). This could easily be added as another Supp Table.

The 94.43% complete BUSCO genes for the gene annotation (proteome) comprise 91.97% that are single copy and 2.46% that are duplicate genes. As suggested, we have created a new Supplementary Table 1, which lists the full BUSCO scores for *Hyles*, and for comparison also for *Manduca* and *Bombyx*. While the BUSCO completeness for *Hyles* and *Bombyx* indicates a low level of duplicated genes, for *Manduca* BUSCO finds 21.95% complete but duplicated genes.

We have now also run BUSCO in whole-genome mode and added these results to Supplementary Table 1. They confirm the high completeness of the *Hyles* genome.

**Minor comments (some of them are encompassed by the general comments):**

- Line 61: sentence is too long, suggest to break it somewhere after "excellent flying abilities."

We split the long sentence, as suggested, and simplified it to:

“With wingspans of 4 to 10 cm, Sphingids are large pollinators with excellent flying abilities. They are important prey for bats …”

- Line 111: include the DAZZLER discussion/pipeline at https://dazzlerblog.wordpress.com/ as an Supp Mat in gigaDB somehow? It seems like a potentially useful resource, that could go missing in the future

Thank you. This is a valid concern. We have now copied the parts of the blog that are relevant for Dazzler as a new Supplementary Text 1 file, which is now part of the Supplement.

- Line 112: "of" -> "is"

Corrected.

- Line 115: "substantially larger by 191 and 232 Mb" - are we sure the increased length is not contributed by contamination of DNA from non-target organisms present in the sample? I am sure this is not the case, but a simple analysis of the final scaffolds (e.g. blobtools, https://github.com/DRL/blobtools) would be informative

Again, we thank the reviewer for raising this valid concern. As described above, application of BlobTools showed a very low level of contamination, which cannot explain the substantially larger genome size.

Rather, we think that expansions of transposons and other repeats contributed to the larger genome size. For example, *Hyles* has 328 Mb in repetitive elements detected by RepeatMasker, which is 117 Mb more than for *Bombyx* (210.5 Mb in repeats). Thus, given that that the *Hyles* genome is 191 Mb larger than the *Bombyx* genome, these 117 Mb of additional repeats explain >60% of the 191 Mb. We added to the text:

“This analysis also suggests that expansions of transposons and other repeats contributed to the larger genome size of *H. vespertilio*, as the *H. vespertilio* assembly comprises 117 Mb more in repetitive elements compared to B. mori (328 vs. 210.5 Mb).”

- Line 115: alternatively, the larger increase could be driven by the presence of allelic regions that are separately assembled and both present in the genome assembly. Is it possible to show a distribution of coverage of the scaffolds? Collapsed regions will show 2X coverage but uncollapsed regions will show 1X coverage. The presence of uncollapsed regions might also affect the Duplication score of BUSCO analysis, which is not provided (see below). Overall, the authors could do a tiny bit more to convince me the increase in genome span is not driven by either (a) contamination and/or (b) the presence of uncollapsed allelic regions. A Kmer distribution from the raw reads would also be useful to ascertain a general level of heterozygosity of this organism

As described above, our read coverage analysis indicates that alternative haplotypes comprise at most 28.7 Mb and thus also cannot solely explain the genome size expansion. A read coverage plot has been added as a new Supplementary Figure 3.

As explained above, while the DAmar assembler includes a read patching step that removes large artifacts in PacBio reads, the assembler does not correct base errors in individual reads. Since PacBio reads have a high base error rate, we cannot compute a Kmer distribution, as it would be possible for Illumina reads.

- Line 138: "much" -> "many"

We have removed ‘much’ from the sentence, as this was not necessary. The sentence now reads:

“This is similar to *B. mori* and *M. sexta*, except that the latter exhibits fewer LINEs.”

- Line 167: please include the complete BUSCO scores, e.g. C,[S,D],F,M, as there is some important information particularly with potential duplication of BUSCO genes

Complete BUSCO scores are now included in a new Supplementary Table 1.

- Line 167: in addition, what are the BUSCO scores for the genome, i.e. run using "-mode genome" vs the genome scaffolds?

We have added BUSCO results for genome mode to Supplementary Table 1 and write now in the main text:

“To assess and compare genome completeness, we used BUSCO [13] and the set of 2,442 conserved, single-copy Endopterygota genes. As shown in Supplementary Table 1, the *H. vespertilio* assembly (95.58% complete genes) shows a similar level of completeness as *B. mori* (96.48%) and *M. sexta* (95.21%).”

- Line 281: again, I suggest the wordpress HTML or text therein be deposited as a Supp Mat for the paper, in case the blog post is lost in the future

We now write: “Third, we used a read alignment step to detect repeats (Supplementary Text 2). To this end, we first split all reads into groups representing 1X read coverage. …”

and provide the rationale behind this step, which was explained in the dazzlerblog, in a new Supplementary Text 2.

- Line 361 and others: what is the rationale for the different Evidence Modeler weightings provided by the various alternative methods? E.g. gene projections are given 8, protein homology 2, etc… why? It would be useful for others if the justification was explained

The weights provided to EVidenceModeler reflect the confidence the user has in the various evidences. The EVidenceModeler user manual (<https://evidencemodeler.github.io/>) provides general guidelines, for example how to weight *de novo* predictions. Following these guidelines, we ranked our evidences and gave the lowest confidence a weight of 1 and the highest, gene projections, a weight of 8.

This is now explained in the text:

“Evidences were ranked and weighted following the guidelines of the EVidenceModeler manual (<https://evidencemodeler.github.io/>). The *ab initio* predictors were given the lowest rank, followed by the spliced alignments. As transcript assembly was done using data from another species, this was ranked second after the gene projections.”

- Line 372: what proportion of the RNA-seq reads from the "closely related" H. euphorbiae mapped to the H. vespertilio genome? The gene annotation of this species is very thorough and so I don't suggest that the authors should have to do this, but it would be interesting to know what the annotation result is using the BRAKER2 pipeline (https://github.com/Gaius-Augustus/BRAKER), if the % RNA-seq reads mapping across the two species is reasonably high

We determined the overall mapping rates for the *H. euphorbiae* read data, made the following additions to the text:

“Reads were mapped to the *H. vespertilio* assembly using hisat2 (v 2.0.0) with parameters “--dta --no-unal --mp 4,1 --score-min L,0,-0.125”, which resulted in mapping 65.82% of the reads.”

and

“To run Augustus [22], we first mapped the RNAseq data again to the genome with using hisat2 and strict mapping parameters (--no-mixed --no-discordant --dta --no-unal --n-ceil L,0,0.05; read mapping rate of 46.79%) to generate hints.”

While this RNA-seq data was from a different species, explaining the lower mapping rates, we found it nevertheless very helpful for annotating genes in our assembly.

We would expect the BRAKER pipeline to produce similar results, as both approaches integrate RNA-seq data, Genemark and training Augustus on high quality models.

- Line 419: see above for general comment on BUSCO - e.g., what is the size of the Endopterygota gene set?

We revised the text at several places:

“To assess and compare genome completeness, we used BUSCO [13] and the set of 2,442 conserved, single-copy Endopterygota genes.”

“…we used BUSCO [13] and the set of 2,442 conserved, single-copy Endopterygota genes. We found that our annotation is highly complete with 94.5% (2,306 of 2,442) of these BUSCO genes being completely present (91.97% single copy, 2.46% duplicated genes).”

**Reviewer #2:**

Nuclear and mitochondrial genomes of the bat hawkmoth were sequenced using PacBio technology and assembled using "a customized assembler called DAmar". This produces impressive assembly statistics [651.4 Mb assembly, 530 contigs, N50 = 7.5 Mb]. In summary, this manuscript is well written and presents an important genome with a very useful accompanying browser.

We thank the reviewer for these kind words.

**Concerns and comments**

1. Chromosomal scaffolding was not performed, which is very useful for comparative genomics.

We agree that chromosomal scaffolding would be very useful. We attempted to produce an optical map using the BioNano Saphyr instrument, but this was not successful, even with help from the company's staff. Nevertheless, our assembly is very contiguous and complete, and therefore we believe that it already represents a useful resource for the community.

2. If this is the first time DAmar has been used in publication, I would recommend a brief explanation in the results and a comparison against one or more available assemblers (e.g. Flye). Is there an advantage using DAmar to assemble lepidopteran genomes over other assemblers? It is important to justify.

We apologize for not making it clearer in the text that DAmar is largely based on Marvel, which is mentioned in the methods:

“This assembler is based on an improved MARVEL assembler [11, 12] and integrates parts from Dazzler suite (Supplementary Text 1) and DACCORD [26, 27].“

Marvel is the assembler that was used to assemble the Axolotl (with 32 GB the largest genome ever assembled so far; Nature 554(7690):50-55, 2018 [Marvel is described in detail in the Supplement of this paper]), *Schmidtea mediterranea* (Nature 554(7690):56-61, 2018), and recently 6 reference-quality bat genomes within the Bat1K project (<https://www.biorxiv.org/content/10.1101/836874v1>). One important feature of Marvel (kept in DAmar) is that this assembler does not break long reads at problematic regions. Instead, problematic regions are repaired using other reads, which preserves long range read contiguity.

We agree that Flye is also a very powerful assembler that could be used in future. However, Flye was published in April 2019, at a time where we were already in the process of annotating our *Hyles* assembly, and we would prefer to not reassemble the genome as every analysis would have to be repeated.

3. "11,721 and 19,760 gene predictions that were projected from B. mori and M. sexta, respectively". I'm interested to know how many of the gene predictions were shared between the two.

We added the following to the text:

“Using a 10% overlap, 15,169 (77%) of the 19,760 *M. sexta* projections overlap a *B. mori* projection and 9,996 (85%) of the 11,721 *B. mori* projections overlap a *M. sexta* projection. 3,336 of the *M. sexta* and *B. mori* projections are identical.”

4. RNAseq data from Hyles euphorbiae was used in one of the steps to develop gene models. It wasn't clear why H. vespertilio mRNA sequence wasn't used? Although an extensive and detailed approach was used to develop gene models, it is not clear if this is better than using RNAseq from H. vespertilio to begin with.

We used available RNA-seq data that was generated from a close relative, as RNA-seq data for *H. vespertilio* was not available. We now clarified this in the text:

“As a third evidence, we used short read RNA sequencing data that was generated for the closely related *H. euphorbiae*, since RNA sequencing data of *H. vespertilio* was not available. “

Nevertheless, we found that this expression data of a close family member was very useful for annotating genes.

5. Line 164. There were 94.5% of BUSCO genes identified, but it should be made clear not all were single copy.

Of the 94.43% complete BUSCO genes, 91.97% are single copy and 2.46% are duplicate genes. We have added this information (full BUSCO scores) to a new Supplementary Table 1 (this was also requested by reviewer 1) and to the text:

“We found that our annotation is highly complete with 94.5% (2,306 of 2,442) of these BUSCO genes being completely present (91.97% single copy, 2.46% duplicated genes).”

In addition, we have now also run BUSCO in whole-genome mode and added these results to Supplementary Table 1. We added to the main text:

“To assess and compare genome completeness, we used BUSCO [13] and the set of 2,442 conserved, single-copy Endopterygota genes. As shown in Supplementary Table 1, the *H. vespertilio* assembly (95.58% complete genes) shows a similar level of completeness as *B. mori* (96.48%) and *M. sexta* (95.21%).”

6. Discussion lines 183-204. This is largely a repetition of the results. I would prefer to see some discussion explaining why 11,721 and 19,760 CESAR predicted genes were identified from B. mori and M. sexta, and then 24567 and 7102 were identified with the GenomeThreader Lepbase gene set. Why are there more M. sexta in the CDS? Why are there 154299 Bicyclus hits and what is the repeat?

We have made many changes in the first Discussion paragraph to shorten it and to reduce repetition. We would like to keep the second discussion paragraph since the strategy to annotate genes is generally applicable to other genomes and we would like to discuss the factors that influence the number of projected genes.

The difference in the number of projected genes or mapped proteins (19760 and 24567 for *M. sexta*; 11721 and 7102 for *B. mori*) is related to the greater evolutionary distance between *Hyles* and *Bombyx*. This is explained in the discussion:

“Evolutionary distance is another factor influencing the completeness of results obtained from gene projections, with closely-related species generally allowing for more complete projections [16], consistent with our result that substantially more genes were projected using *M. sexta* as a reference.“

The large number of hits for Bicyclus is likely explained by including several transposon-derived ORFs in the gene set. We added this to the Supplementary Table 3 legend:

“* The exceptionally large number of 154299 alignments for *Bicyclus anynana* is most likely explained by the inclusion of transposon-derived ORFs in the gene set of this species. For example, the top 3 proteins with the most alignments to the *Hyles* genome align 10970, 10226 and 10079 times, respectively. A BLAST search against NCBI RefSeq revealed that these three peptides are fragments of reverse transcriptases. “

7. Figure 2B. This presents a treemap comparison of the H. vespertilio MARVEL assembly - do you mean the DAmar assembly?

We thank the reviewer for noticing this mistake. We corrected the legend:

“Treemap comparison between the DAmar *H. vespertilio* assembly and the assemblies of B. mori [6] and M. sexta [7].”
